# Macrophage mitochondrial bioenergetics and tissue invasion are boosted by an Atossa‐Porthos axis in Drosophila

**DOI:** 10.15252/embj.2021109049

**Published:** 2022-03-23

**Authors:** Shamsi Emtenani, Elliot T Martin, Attila Gyoergy, Julia Bicher, Jakob‐Wendelin Genger, Thomas Köcher, Maria Akhmanova, Mariana Guarda, Marko Roblek, Andreas Bergthaler, Thomas R Hurd, Prashanth Rangan, Daria E Siekhaus

**Affiliations:** ^1^ Institute of Science and Technology Austria Klosterneuburg Austria; ^2^ Department of Biological Sciences RNA Institute University at Albany Albany NY USA; ^3^ CeMM Research Center for Molecular Medicine of the Austrian Academy of Sciences Vienna Austria; ^4^ Vienna BioCenter Core Facilities Vienna Austria; ^5^ Department of Molecular Genetics University of Toronto Toronto ON Canada

**Keywords:** immune cell infiltration, mitochondrial bioenergetics, oxidative phosphorylation, protein translation, transcription factor, Immunology, Membranes & Trafficking, Metabolism

## Abstract

Cellular metabolism must adapt to changing demands to enable homeostasis. During immune responses or cancer metastasis, cells leading migration into challenging environments require an energy boost, but what controls this capacity is unclear. Here, we study a previously uncharacterized nuclear protein, Atossa (encoded by *CG9005*), which supports macrophage invasion into the germband of Drosophila by controlling cellular metabolism. First, nuclear Atossa increases mRNA levels of Porthos, a DEAD‐box protein, and of two metabolic enzymes, lysine‐α‐ketoglutarate reductase (LKR/SDH) and NADPH glyoxylate reductase (GR/HPR), thus enhancing mitochondrial bioenergetics. Then Porthos supports ribosome assembly and thereby raises the translational efficiency of a subset of mRNAs, including those affecting mitochondrial functions, the electron transport chain, and metabolism. Mitochondrial respiration measurements, metabolomics, and live imaging indicate that Atossa and Porthos power up OxPhos and energy production to promote the forging of a path into tissues by leading macrophages. Since many crucial physiological responses require increases in mitochondrial energy output, this previously undescribed genetic program may modulate a wide range of cellular behaviors.

## Graphical abstract

A new pathway couples increased metabolic enzyme expression and translation of mitochondrial OXPHOS complex proteins to boost pioneer cell invasion.

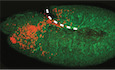

## Introduction

Charged with protecting the organism against continuously changing threats, the immune system must constantly adapt, altering the location, number, and differentiation status of its different immune cell subtypes (Nicholson, 2016). Such continuous adjustment requires high levels of energy. How immune cells satisfy these increased metabolic requirements is just beginning to be understood (O'Neill *et al*, 2016; Guak & Krawczyk, 2020). The main energy currency in the cell is ATP, produced from carbohydrates by cytoplasmic glycolysis and the mitochondrial TCA cycle that feeds electron donors into oxidative phosphorylation (OxPhos) complexes I through IV, components in the electron transport chain (ETC). Anaerobic glycolysis is quick, but respiratory OxPhos extracts considerably more ATP from a single molecule of glucose, albeit more slowly (Berg *et al*, 2002). OxPhos is most directly regulated by the activity and the amount of complexes I through V that carry it out (Hüttemann *et al*, 2007). Upregulation of OxPhos is known to be required for many important immune cell functions, such as B cell antibody production (Price *et al*, 2018), pathogenic T‐cell differentiation during autoimmunity (Shin *et al*, 2020), CD8^+^ memory T‐cell development and expansion (van der Windt *et al*, 2012), T‐reg suppressive function (Weinberg *et al*, 2019), T cell activation by macrophages (Kiritsy *et al*, 2021), and the maturation of anti‐inflammatory macrophages (Vats *et al*, 2006). However, what changes immune cells initiate to upregulate OxPhos remains unclear and how such shifts in metabolism could influence immune cell migration is unexplored.

Immune cells move within the organism to enable distribution and maturation (Kierdorf *et al*, 2015) and to respond to homeostatic challenges, injuries, tumors, or infections (Luster *et al*, 2005; Ratheesh *et al*, 2015). To migrate across unimpeded environments, cells expend energy restructuring their actin cytoskeleton, activating myosin ATPase and reorganizing their cell membrane (Cuvelier *et al*, 2007). Even greater energy requirements exist when cells must also remodel their surroundings as they move ahead against the resistance of flanking cells or extracellular matrix (Zanotelli *et al*, 2018, 2019; Kelley *et al*, 2019). Most *in vitro* or *in vivo* studies on the metabolism that enables the migration of diverse immune cell types have highlighted the importance of glycolysis (Semba *et al*, 2016; Guak *et al*, 2018; Kishore *et al*, 2018). To our knowledge, only one study has demonstrated a need for a functional ETC, to speed neutrophil migration *in vivo* potentially by enabling the polarized secretion of ATP to amplify guidance cues (Zhou *et al*, 2018). Increases in OxPhos triggered by PGC‐1’s transcriptional upregulation of mitochondrial proteins can underlie enhanced invasion and metastasis in some cancer types and suppress it in others (LeBleu *et al*, 2014; Torrano *et al*, 2016; Davis *et al*, 2020). OxPhos has been shown to be particularly required in the first cancer cell leading coordinated chains into challenging environments *in vitro* (Khalil & Friedl, 2010; Commander *et al*, 2020); these leader cells have been shown to need higher ATP levels to create a path (Zhang *et al*, 2019). Although the ability of immune cells to invade tissues or tumors also depends on movement against surrounding resistance, it is not known if immune cells similarly require enhanced levels of OxPhos for such infiltration and if they do how they achieve this energy boost.

To identify new mechanisms governing *in vivo* migration, we study *Drosophila* macrophages, also called plasmatocytes. Macrophages are the primary innate immune cell in *Drosophila* and share remarkable similarities with vertebrate macrophages in ontogeny, functions, and migratory behavior (Ratheesh *et al*, 2015; Wood & Martin, 2017). These macrophages not only resolve infections, but also influence development and homeostasis (Bunt *et al*, 2010; Buck *et al*, 2016; Caputa *et al*, 2019; Riera‐Domingo *et al*, 2020). To reach places where they are needed to enable proper development, some macrophages follow guidance cues and invade the extended germband between the closely apposed ectoderm and mesodermal tissues, moving against the resistance of surrounding tissues (Siekhaus *et al*, 2010; Ratheesh *et al*, 2018; Valoskova *et al*, 2019; Belyaeva *et al*, 2022). Importantly, the rate‐limiting step for this tissue invasion is the infiltration of the pioneer macrophage, a process affected both by the properties of the surrounding tissues (Ratheesh *et al*, 2018) as well as macrophages themselves (Valoskova *et al*, 2019; Belyaeva *et al*, 2022). Here we identify a previously uncharacterized pathway that induces concerted metabolic and mitochondrial reprogramming to support the higher energy levels needed for pioneer cell invasion through changes in translation and metabolic enzyme expression. Our data lay the foundation for mammalian studies on diverse pathological conditions, from autoimmunity to cancer, as well as those independent of migration.

## Results

### *CG9005* is required in macrophages for their early invasion into the extended germband

To find new molecular pathways potentially mediating germband invasion, we examined the BDGP *in situ* project and identified CG9005 as a previously uncharacterized gene whose mRNA is enriched in macrophages prior to and during germband tissue entry (BDGP *in situ* of CG9005 mRNA) (Tomancak *et al*, 2002, 2007). CG9005 mRNA is maternally deposited and expressed in the mesoderm, including the region in which macrophages are specified during Stage 4–6. CG9005 is further upregulated in macrophages starting at Stage 7 while its expression decreases in the remaining mesoderm. CG9005 mRNA remains expressed during Stages 9–12 in macrophages, during their ingression, dissemination, and movement toward and into the germband. After invasion, CG9005 mRNA is downregulated in macrophages to match the lower expression levels found ubiquitously in the embryo.

**Figure 1:**
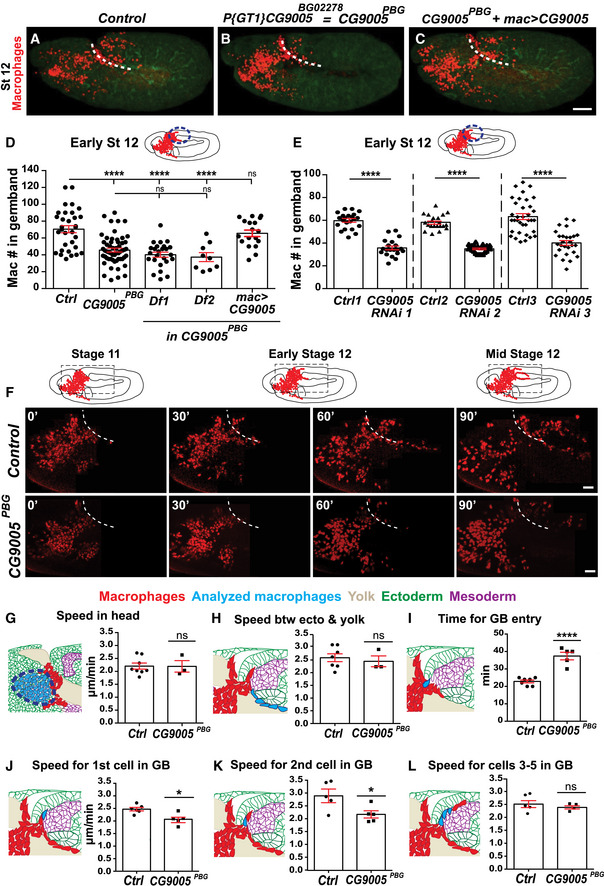
CG9005 acts in macrophages to spur pioneer cell infiltration into the germband tissue A–CConfocal images of Stage 12 embryos from control, *P{GT1}CG9005^BG02278^* P‐element mutant (*CG9005^PBG^*), and *CG9005^PBG^* with *CG9005* expression restored in macrophages. Macrophage: red. Phalloidin to visualize embryo: green. Germband edge: dotted white line.DQuantification of macrophages that have penetrated the germband from genotypes in (A‐C) and from *CG9005^PBG^* over two deficiencies (Df) that remove the gene. *n* = 35, 56, 25, 9, 18 embryos, respectively; *P* < 0.0001 for control versus *CG9005^PBG^*, Df1, or Df2; *P* = 0.98 for control versus *mac>CG9005* rescue; *P* = 0.91, 0.90 for *CG9005^PBG^* versus Df1 or Df2.EMacrophage‐specific knockdown of *CG9005* by *UAS‐RNAi* lines. *n* = 22, 20, 21, 23, 35, 28 embryos. *P* < 0.0001 for all comparisons.FStills from two‐photon movies of control and *CG9005^PBG^* mutant embryos showing macrophages (nuclei, red) migrating starting at Stage 10 from the head toward the germband and invading the germband tissue. Elapsed time indicated in minutes. Germband edge (white dotted line) detected by yolk autofluorescence. For quantification of migration parameters in movies see (G‐L).G, HMacrophage migration speed (G) in the head or (H) between the yolk sac and the germband edge. For (G): control *n* = 8 movies, *CG9005^PBG^* mutant *n* = 3; control *n* = 360 tracks, mutant *n* = 450, *P* = 0.65. For (H): control *n* = 7 movies, mutant *n* = 3; control *n* = 46 tracks, mutant *n* = 19, *P* = 0.62.IThe time required for the first macrophage nucleus to enter into the extended germband. Control *n* = 7 movies, mutant *n* = 5. Time to entry: control = 23 min, *CG9005^PBG^* = 38 min, *P* < 0.0001.J–LThe migration speed of the (J) 1^st^, (K) 2^nd^, or (L) 3^rd^‐5^th^ macrophages along the first 25–30 µm into the germband between the mesoderm and ectoderm. In schematics, analyzed macrophages—light blue, other macrophages—red, ectoderm—green, mesoderm—purple, and yolk—beige. For (J): control *n* = 6 movies, mutant *n* = 5, *P* = 0.012. For (K): control *n* = 5 movies, mutant *n* = 5, *P* = 0.03. For (L): control *n* = 5 movies, mutant *n* = 4, *P* = 0.17. Data information: Scale bars: 50 µm (A–C), 30 µm (F). Throughout paper mac> indicates GAL4 driven expression of a UAS construct specifically in macrophages by *srpHemo‐GAL4*. N for movies represents imaging from different embryos. Throughout this work, embryos with stomodeal invagination and germband retraction away from the anterior of < 29% were defined as Stage 10, 29–31% Stage 11, and 35–40% Stage 12. (D) One‐way ANOVA with Tukey. (E and G‐L) Unpaired *t*‐tests. Graphs show mean ± SEM; ns = *P* > 0.05, *P < 0.05, ****P < 0.0001. See Source Data 1 and 2 for Fig 1. Source data are available online for this figure.

**Figure EV1:**
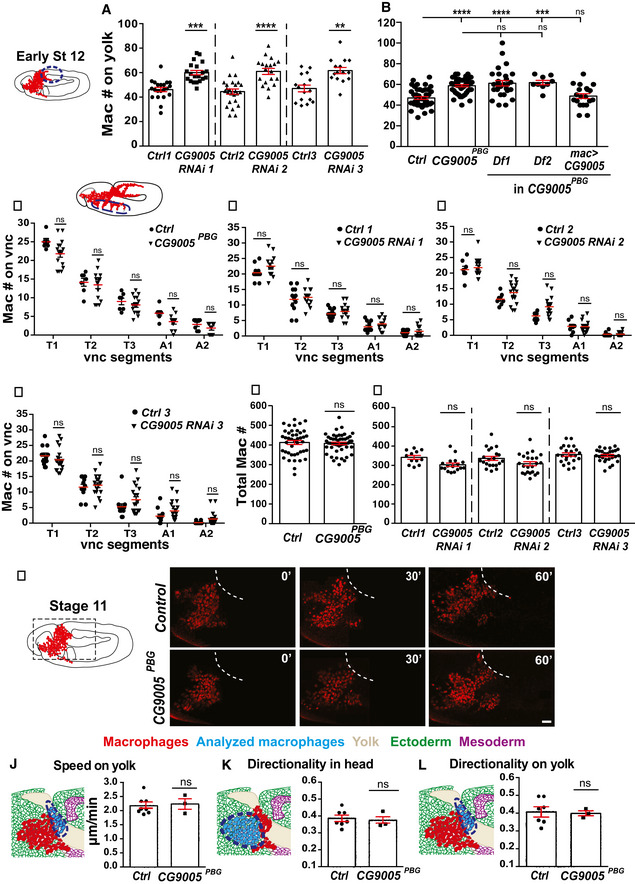
*CG9005^PBG^* mutant macrophages migrate normally within the head and along the vnc A, BQuantification of macrophages in fixed early Stage 12 embryos shows a significant increase on the yolk in (A) lines expressing each of the CG9005 RNAis specifically in macrophages (*mac>)* and in (B) the P element mutant *CG9005^PBG^* compared to the control. For (A) control (*n* = 43 embryos) versus *CG9005^PB^* mutant (*n* = 50), *CG9005^PB^* mutant/*Df1* (*n* = 28) or *CG9005^PB^* mutant/*Df2* (*n* = 9), all *P* < 0.0001. Control versus *CG9005^PB^* mutant with *mac>CG9005* rescue (*n* = 20) *P* = 0.99. *CG9005^PB^* mutant alone versus, mutant with *mac>CG9005* rescue *P* = 0.001. For (B) control 1 (*n* = 21 embryos) versus *CG9005 RNAi* 1 (*n* = 20) *P* = 0.0002; control 2 (*n* = 25) versus *CG9005 RNAi* 2 (*n* = 19) *P* < 0.0001; control 3 (*n* = 16) versus *CG9005 RNAi* 3 (*n* = 15) *P* = 0.001.C–FMacrophage quantification in ventral nerve cord (vnc) segments reveals no significant difference in macrophage migration along the vnc between (C) *CG9005^PBG^* mutant and control embryos or (D‐F) *mac>CG9005 RNAi* embryos compared to the controls. For (C) control (*n* = 7 embryos) versus *CG9005^PB^* mutant (*n* = 15) *P* > 0.05. For (D) control 1 (*n* = 8 embryos) versus *CG9005 RNAi* 1 (*n* = 13) *P* = 0.25; for (E) control 2 (*n* = 8 embryos) versus *CG9005 RNAi* 2 (*n* = 16) *P* = 0.5; for (F) control 3 (*n* = 8 embryos) versus *CG9005 RNAi* 3 (*n* = 16) *P* > 0.99.G, HQuantification of the total macrophage number reveals no significant difference between (G) the control and *CG9005^PBG^* mutant embryos, or (H) the control and *mac>CG9005 RNAi* embryos. For (G) control (*n* = 43 embryos) versus *CG9005^PBG^* mutant (*n* = 50) *P* = 0.69. For (H) control 1 (*n* = 12 embryos) versus *CG9005 RNAi* 1 (*n* = 17) *P* = 0.9; control 2 (*n* = 27) versus *CG9005 RNAi* 2 (*n* = 19) *P* = 0.84; control 3 (*n* = 23) versus *CG9005 RNAi* 3 (*n* = 27) *P* = 0.16.IStills from two‐photon movies of control and *CG9005^PBG^* mutant embryos, showing macrophages migrating starting at Stage 10 from the head toward the germband. Elapsed time indicated in minutes. The germband edge (white dotted line) was detected by yolk autofluorescence. For quantification of migration parameters from two‐photon live imaging of macrophages, see (J‐L).JMacrophages on the yolk sac in the *CG9005^PBG^* mutant reach the germband with a similar speed to control macrophages. Speed: control and mutant = 2.2 µm/min, *P* = 0.78; control *n* = 8 movies, mutant *n* = 3; control *n* = 373 tracks, mutant *n* = 124.K, LMacrophage directionality (K) in the head or (L) on the yolk sac shows no change in the *CG9005^PBG^* mutant compared to the control. For (K) head directionality: control = 0.39, mutant = 0.37, *P* = 0.74; control *n* = 7 movies, mutant *n* = 3. For (L) yolk sac directionality: control = 0.40, mutant = 0.39, *P* = 0.86; control *n* = 7 movies, mutant *n* = 3. Data information: Macrophages analyzed in (A‐L) were labeled with *srpHemo‐H2A::3xmCherry* to visualize nuclei. In schematics, macrophages are shown in red and analyzed macrophages in light blue, the ectoderm in green, the mesoderm in purple, and the yolk in beige. Throughout this work *mac>* indicates *srpHemo‐GAL4* driving UAS constructs specifically in macrophages. Mean ± SEM, ns=*P* > 0.05, **P < 0.01, ***P < 0.001, ****P < 0.0001. One‐way ANOVA with Tukey (A) and unpaired *t*‐test (B‐H) and (J‐L). Scale bar: 30 µm (I). See Source Data 1 and 2 for Fig EV1. Source data are available online for this figure.

We examined a P‐element insertion allele, *CG9005^BG02278^* (*CG9005^PBG^*), visualizing macrophages with a nuclear fluorescent marker. Quantification revealed a 36% decrease in macrophages within the germband in *CG9005^PBG^* mutant embryos compared to the control (Fig 1A, B and D), similar to *CG9005^PBG^* placed over either *Df(2R)ED2222* or *Df(2R)BSC259* that remove the gene entirely (Fig 1D), demonstrating the allele is a genetic null for invasion. Expressing CG9005 in macrophages in the mutant completely restored their capacity to invade the germband (Fig 1C and D). Driving any of three independent *CG9005* RNA interference (RNAi) lines in macrophages decreased macrophages within the germband by 37–40% compared to controls (Fig 1E) and increased macrophages sitting on the yolk near the entry site that have not yet invaded the germband (Fig EV1A) by 24–27%, a shift also seen in *CG9005^PBG^* (Fig EV1B). We counted macrophages migrating along the ventral nerve cord (vnc) in late Stage 12 embryos, a route guided by the same factors that lead into the germband (Wood & Martin, 2017) but not requiring tissue invasion (Siekhaus *et al*, 2010; Ratheesh *et al*, 2018). There was no significant difference in the *CG9005^PBG^* mutant (Fig EV1C) and the *CG9005* RNAi‐expressing macrophages (Fig EV1D–F) compared to their controls, arguing that basic migratory processes and recognition of chemotactic signals are unperturbed. Moreover, we detected no significant change in the total number of macrophages for these genotypes (Fig EV1G and H). Taken together, these results from fixed embryos indicate that CG9005 is specifically required in macrophages for the early steps of germband invasion.

### Atossa (CG9005) promotes efficient invasion of pioneer macrophages into the germband tissue

To directly assess CG9005’s role in germband invasion, we conducted two‐photon live imaging in control and *CG9005^PBG^* embryos, visualizing macrophage nuclei with *srpHemo‐H2A::3xmCherry* (Figs 1F and EV1I, Movies EV1 and EV2). We observed no significant change in *CG9005^PBG^* in macrophage speed or directionality during their migration starting at Stage 9 from the head mesoderm up to the yolk neighboring the germband entry point and beyond between the yolk and the ectoderm (Figs 1G and H, and EV1J–L) (Speed in the head and on yolk: 2.2 µm/min for control and *CG9005^PBG^*; *P* = 0.65, *P* = 0.78, respectively. Directionality: 0.39 in control, 0.37 in mutant in both regions, *P* = 0.74 for head, *P* = 0.86 for yolk. Speed along ectoderm control = 2.6, *CG9005^PBG^* = 2.5 µm/min, *P* = 0.62). However, the first macrophage in *CG9005^PBG^* required 65% more time than the control to enter into the germband tissue (time to entry: control = 23 min, *CG9005^PBG^* = 38 min, *P* < 0.0001) (Fig 1I). The speed of the first two pioneering macrophages is also significantly slower as they invade along the path between the mesoderm and ectoderm in *CG9005^PBG^* mutant embryos compared to the control (Fig 1J and K) (1^st^ cell: control = 2.5, *CG9005^PBG^* = 2 µm/min, *P* = 0.012; 2^nd^ cell: control = 2.9, *CG9005^PBG^* = 2.1 µm/min, *P* = 0.03). However, the speed of the next few cells migrating along this path was not affected (Fig 1L) (3^rd^–5^th^ cells: control = 2.5, *CG9005^PBG^* = 2.4 µm/min, *P* = 0.17). We conclude that CG9005 specifically regulates initial tissue invasion, facilitating the entry into and subsequent movement within the germband tissue of the first two pioneer macrophages. Since the macrophage stream into the germband becomes much reduced in *CG9005^PBG^*, we called the gene *atossa* (*atos*), for the powerful Persian queen whose name means trickling.

### Atossa (CG9005) is a nuclear protein whose conserved motifs and TADs are important for macrophage tissue invasion

**Figure 2:**
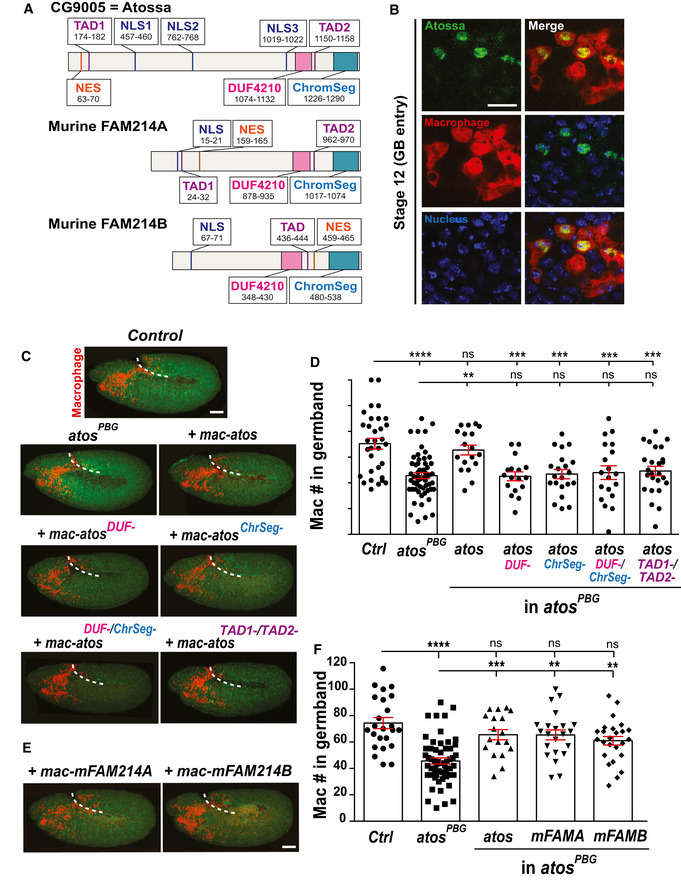
CG9005/Atossa requires conserved domains linked to transcriptional activation to enhance tissue invasion, a function maintained by its mammalian orthologs ADeduced protein structure of *Drosophila* CG9005/Atossa (Atos) and its murine orthologs, mFAM214A‐B, highlighting conserved domains. FAM214A‐B are 44–45% identical to Atos.BMacrophages (red) near the germband in Stage 11/12 embryos. Atos tagged at N terminus with HA (HA‐antibody, green) and expressed under direct control of macrophage‐specific promoter. Nucleus stained by DAPI (blue).C, DConfocal images or (D) quantification of the macrophages in germband in Stage 12 embryos from the control, *atos^PBG^*, and *atos^PBG^* expressing Atos itself or variants lacking particular domains. Transgene expression directly from macrophage‐specific promoter (*mac‐*). For control (*n* = 32 embryos) versus *atos^PBG^* mutant (*n* = 56) *P* < 0.0001; versus rescue with *mac‐atos* (*n* = 18) *P* > 0.99; versus rescue with *mac‐atos^DUF‐^* (*n* = 17) *P* = 0.0003; versus rescue with *mac‐atos* ^ChrSeg‐^(*n* = 21) *P* = 0.0003; versus rescue with *mac‐atos^DUF‐/^* ^ChrSeg‐^(*n* = 19) *P* = 0.00014; versus rescue with *mac‐atos* ^TAD1‐/ TAD2‐^ (*n* = 25) *P* = 0.0009, *atos^PBG^* mutant versus rescue with *mac‐atos P* = 0.0031.EConfocal images of *atos^PBG^* rescued by expressing Atossa’s murine orthologs, mFAM214A or B (mFAMA‐B) in macrophages,FQuantification of macrophages in the germband in Stage 12 embryos from the control, *atos^PBG^*, and *atos^PBG^* embryos expressing *mFAM214A* or *B* specifically in macrophages (*mac‐*). For control (*n* = 24 embryos) versus *atos^PBG^* mutant (*n* = 56) *P* < 0.0001; versus *mac‐atos* rescue (*n* = 18) *P* = 0.7; versus *mac‐mFAMA* rescue (*n* = 22) *P* = 0.6; versus *mac‐mFAMB* rescue (*n* = 25) *P* = 0.086. For *atos^PBG^* mutant versus *mac‐atos* rescue *P* = 0.0006; versus *mac‐mFAMA* rescue *P* = 0.0002; versus *mac‐mFAMB* rescue *P* = 0.0043. Data information: Germband edge: dotted white line. Mac indicates direct expression from the srpHemo promoter. (C, E) Macrophage nuclei (red), actin by Phalloidin staining (green). (D,F) One‐way ANOVA with Tukey. Mean ± SEM, ns=*P* > 0.05, **P < 0.01, ***P < 0.001, ****P < 0.0001. Scale bars: 5 µm (B), 50 µm (C, E). See Source Data 1 and 2 for Fig 2. Source data are available online for this figure.

**Figure EV2:**
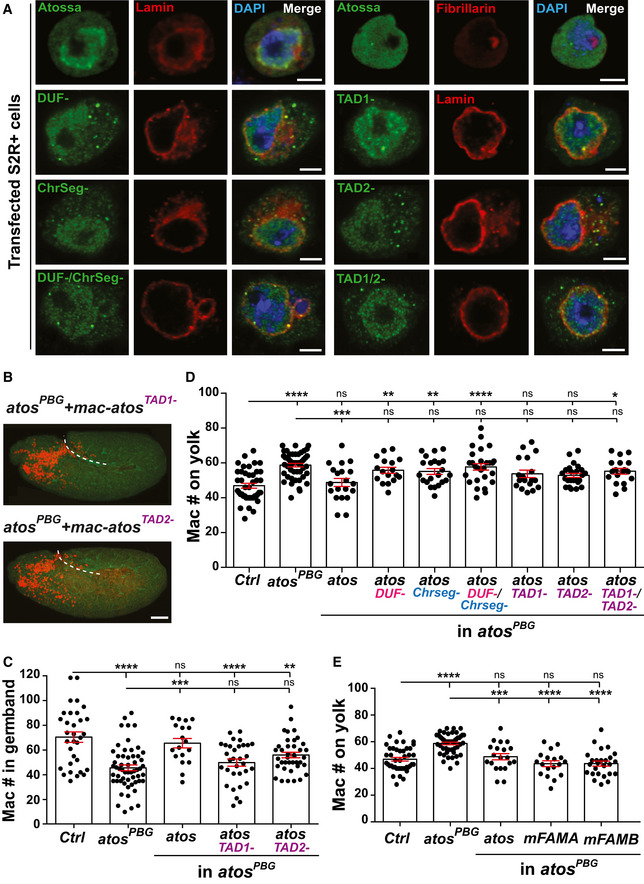
Atos’s TAD domains are essential in macrophages for their tissue infiltration AS2R^+^ cells were transfected with wild‐type Atos or forms lacking the indicated domains. N terminally HA‐tagged Atos (green), the nuclear membrane marker Lamin (red), and the nucleolar marker Fibrillarin (red) were visualized with antibodies, and nuclear DNA with DAPI (blue). All forms of Atos are expressed under direct control of the macrophage‐specific *srpHemo* promoter (*mac‐*).BRepresentative confocal images of Stage 12 embryos from *atos^PBG^* mutants expressing Atos lacking either TAD1 or 2 in macrophages. Macrophage nuclei (red) are visualized with a transgene and the embryo outlines with phalloidin staining (green). Germband edge: dotted white line.CQuantification shows that deletion of TAD1 or 2 blocks Atos’s ability to rescue the germband migration defect of Stage 12 *atos^PBG^* mutant embryos upon expression in macrophages. For control (*n* = 32 embryos) versus *atos^PBG^* mutant (*n* = 56) *P* < 0.0001; *mac‐atos* rescue (*n* = 18) *P* > 0.99; *mac‐atos^TAD1‐^* rescue (*n* = 32) *P* < 0.0001; *mac‐atos^TAD2‐^* rescue (*n* = 39) *P* = 0.008. For *atos^PBG^* mutant versus *atos* rescue *P* = 0.0006, versus *atos^TAD1‐^* rescue *P* = 0.97, versus *atos^TAD2‐^* rescue *P* = 0.06.DQuantification in fixed early Stage 12 embryos shows a significant increase compared to control embryos in the number of macrophages on the yolk in the mutant alone and in the *atos^PBG^* mutant expressing forms of *atos* lacking the DUF or Chrseg domains or both TAD domains. Significant difference was observed compared to the *atos^PBG^* mutant only upon expression of wild‐type *atos*. For control (*n* = 43 embryos) versus *atos^PBG^* mutant (*n* = 50) *P* < 0.0001; versus *atos* rescue (*n* = 20) *P* > 0.99; versus *atos^DUF‐^* rescue (*n* = 17) *P* = 0.0076; versus *atos* ^ChrSeg‐^ rescue (*n* = 22) *P* = 0.0066; versus *atos^DUF‐/^* ^ChrSeg‐^ rescue (*n* = 27) *P* < 0.0001; versus *atos^TAD1‐^* rescue (*n* = 18) *P* = 0.12; versus *mac‐atos^TAD2‐^* rescue (*n* = 24) *P* = 0.18; versus *mac‐atos* ^TAD1‐/ TAD2‐^ rescue (*n* = 18) *P* = 0.013. For *atos^PBG^* mutant versus *atos* rescue *P* = 0.0003, versus *atos^DUF‐^* rescue *P* > 0.99; versus *atos* ^ChrSeg‐^ rescue *P* > 0.99; versus *atos^DUF‐/^* ^ChrSeg‐^ rescue *P* > 0.99, versus *atos^TAD1‐^* rescue *P* > 0.99, versus *atos^TA2‐^* rescue *P* = 0.15, versus *atos^TAD1‐/TAD2‐^* rescue *P* > 0.99.EQuantification shows a similar number of macrophages on the yolk in fixed early Stage 12 *atos^PBG^* mutant embryos which express *mFAM214A* or *mFAM214B* in macrophages compared to the control. For control (*n* = 43 embryos) versus *atos^PBG^* mutant (*n* = 50) *P* < 0.0001; control versus *mac‐mFAM214A* rescue (*n* = 18) *P* = 0.65; control versus *mac‐mFAM214B* rescue (*n* = 26) *P* = 0.56; *atos^PBG^* mutant versus *mac‐atos* rescue (*n* = 20), *mac‐mFAM214A* and *mac‐mFAM214B* rescues *P* < 0.0001. Data information: Macrophages visualized with *srpHemo‐H2A::3xmCherry* expression throughout mean ± SEM, ns = *P* > 0.05, *P < 0.05, **P < 0.01, ***P < 0.001, ****P < 0.0001. One‐way ANOVA with Tukey (C‐E). Scale bars: 3 µm (A), 50 µm (B). See Source Data 1 and 2 for Fig EV2. Source data are available online for this figure.

Atossa (Atos) contains a conserved domain of unknown function (DUF4210) and a chromosome segregation domain (Chr_Seg) (Fig 2A). Atos also displays two trans‐activating domains (TADs) common among transcription factors, three nuclear localization signals (NLS), and a nuclear export signal (NES). We found FLAG::HA‐tagged Atos mainly in the nucleus in embryonic macrophages *in vivo* (Fig 2B) and in macrophage‐like S2R^+^ cells where it was also partially in the cytoplasm (Fig EV2A). Atos mutant forms lacking the conserved domains and TADs were similarly present in the nucleus (Fig EV2A) yet were unable to rescue germband invasion (Figs 2C and D, and EV2B and C). Consistent with a germband invasion defect, *atos^PBG^* embryos expressing these *atos* mutants had more macrophages sitting on the yolk at the germband entry site prior to invasion than those expressing wild‐type Atos (Fig EV2D). Atos is 40% identical to its uncharacterized murine orthologs, mFAM214A‐B, which maintain these domains (Fig 2A). Expression in macrophages of either mFAM214A or B in *atos^PBG^* rescued the germband invasion defect as efficiently as the *Drosophila* protein itself (Fig 2E and F) and restored the normal number of macrophages on the yolk neighboring the extended germband (Fig EV2E). These data clearly show that the conserved domains and TADs are critical for the primarily nuclear protein, Atos, to facilitate macrophage invasion.

### Atos raises mRNA levels of a DEAD‐box protein and metabolic enzymes, which are each required for germband invasion

**Figure EV3:**
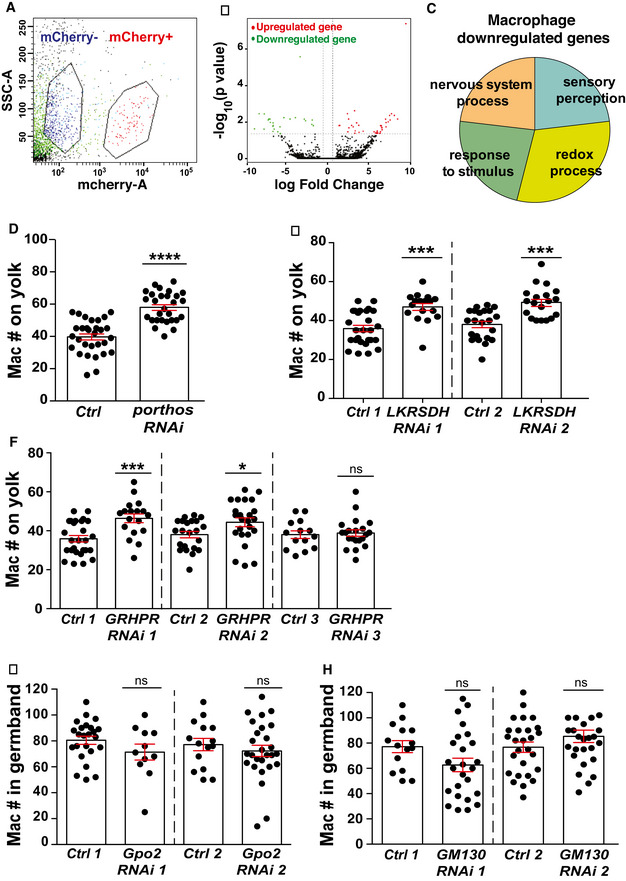
Macrophage transcriptome analysis reveals that Atos targets participate in signaling, cell communication, and ion transport AFACS plot of Side Scatter (SSC) versus mCherry fluorescence signal in macrophages obtained from embryos expressing *srpHemo‐3xmCherry*. The two populations are sorted as mCherry marker + (red) and – (blue) cells.BGenes expressed differentially in analysis of RNA sequencing data from macrophages from the *atos^PBG^* mutant compared to the control are shown in a volcano plot graphing the log_10_ of the *P*‐value against the log fold change (FC) of the mean normalized expression levels. Each point represents the average value of one gene’s expression from four replicate experiments. Dotted vertical lines indicate a log_10_ fold change ≥ 1 and the dotted horizontal line a *P*‐value of ≤ 0.05. Statistically significant up‐ and downregulated genes are reported as red and green dots, respectively.CGene ontology (GO) analysis of downregulated genes from *atos^PBG^* mutant macrophages compared to the control shows that these genes are involved in oxidation‐reduction processes, stress responses, as well as the nervous system.D–FQuantification reveals that expression of RNAis against *porthos*, *LKR/SDH* and *GR/HPR*, in macrophages leads to a significant increase in macrophage numbers on the yolk in fixed early Stage 12 embryos compared to their controls. For (D) control (*n* = 30 embryos) versus *porthos* RNAi *n* = 28, (E) control 1 (*n* = 27) versus *LKR/SDH RNAi* 1 (VDRC 51346, *n* = 17) and control 2 (*n* = 22) versus *LKR/SDH RNAi* 2 (VDRC 109650, *n* = 19), all *P* < 0.0001. For (F) control 1 (*n* = 27) versus *GR/HPR RNAi* 1 (VDRC 44653, *n* = 18) *P* = 0.0004; control 2 (*n* = 22) versus *GR/HPR RNAi* 2 (VDRC 107680, *n* = 24) *P* = 0.03; control 3 (*n* = 14) versus *GR/HPR RNAi* 3 (VDRC 64652, *n* = 21) *P* = 0.7.G, HQuantification in fixed early Stage 12 embryos reveals that knockdown by two different RNAis of (G) *Glycerophosphate oxidase* 2 (*Gpo2*, *CG2137*) or (H) *Golgi matrix protein 130 kD* (*GM130*, CG11061) did not change the macrophage number within the germband compared to their controls. For (G) control 1 (*n* = 24 embryos) versus *Gpo2 RNAi* 1 (VDRC 41234, *n* = 11) *P* = 0.26; control 2 (*n* = 15) versus *Gpo2 RNAi* 2 (VDRC 68145, *n* = 27) *P* = 0.38. For (H) control 1 (*n* = 15 embryos) versus *GM130 RNAi* 1 (VDRC 330284, *n* = 25) *P* = 0.14; control 2 (*n* = 27) versus *GM130 RNAi* 2 (VDRC 64920, *n* = 20) *P* = 0.34. Data information: Mean ± SEM, ns=*P* > 0.05, *P < 0.05, ***P < 0.001, ****P < 0.0001. Unpaired *t*‐test for (D‐H). See Source Data 1 for Fig EV3. Source data are available online for this figure.

**Figure 3:**
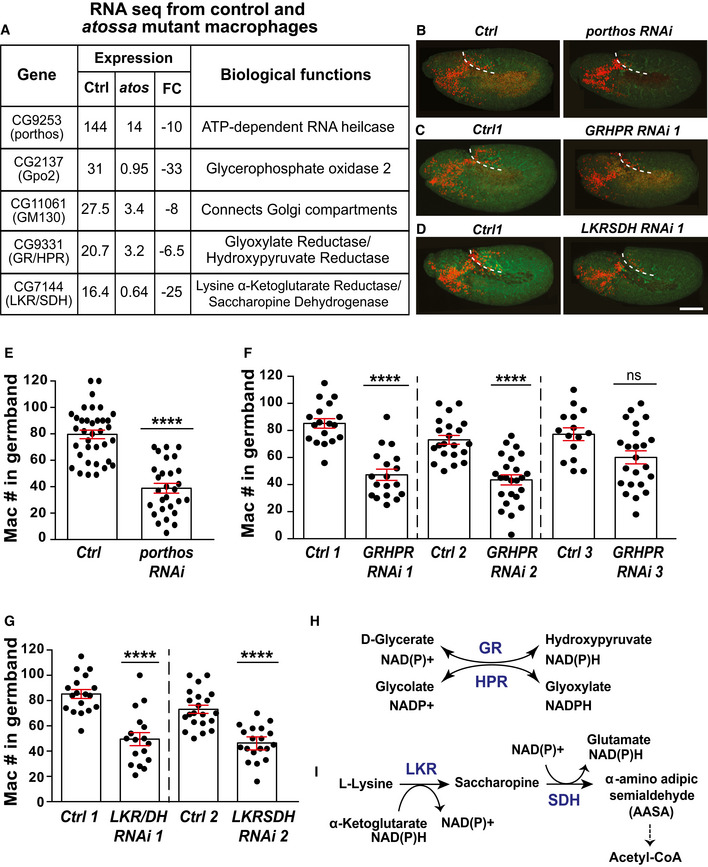
Atos leads to higher mRNA levels of a Ddx protein and metabolic enzymes required for germband invasion ASelection of genes downregulated in *atos^PBG^* mutant macrophages compared to the control, chosen for their > 5 fold change in expression as well as an identified biological function.B–DConfocal images of early Stage 12 embryos from the control and lines expressing *RNAis* against (B) *porthos*, (C) *GR/HPR*, or (D) *LKR/SDH*, specifically in macrophages (red). Germband edge: dotted white line.E–GQuantification of the number of germband macrophages in embryos from control and upon RNAi knockdown of the genes in B‐D. In (E), control (*n* = 36 embryos) versus *porthos RNAi* (*n* = 28) *P* < 0.0001. In (F), control 1 (*n* = 18 embryos) versus *GR/HPR RNAi 1* (*n* = 18) and control 2 (*n* = 21) versus *GR/HPR RNAi* 2 (*n* = 24), both *P* < 0.0001; control 3 (*n* = 15 embryos) versus *GR/HPR RNAi 3* (*n* = 23) *P* = 0.08. In (G) control 1 (*n* = 18 embryos) versus *LKR/SDH RNAi 1* (*n* = 17) and control 2 (*n* = 21) versus *LKR/SDH RNAi 2* (*n* = 23), both *P* < 0.0001.HSchematic: Glyoxlate reductase/hydroxypyruvate reductase (GR/HPR) catalyzes the reduction of glyoxylate into glycolate and converts hydroxypyruvate into D‐glycerate by oxidation of the cofactor NAD(P)H.ISchematic: Lysine α‐ketoglutarate reductase/saccharopine dehydrogenase (LKR/SDH) catalyzes the first two steps of the lysine catabolism pathway, resulting in the production of glutamate and acetyl CoA, a TCA substrate, through several downstream enzymatic reactions. Data information: Glu: Glutamate, α‐KG: α‐ketoglutarate, AASA: α‐aminoadipate δ‐semialdehyde. Mean ± SEM and ns = *P* > 0.05, ****P < 0.0001. One‐way ANOVA with Tukey (E‐G). Scale bar: 50 µm (B‐D). See Source Data 1 for Fig 3. Source data are available online for this figure.

To identify Atos’ potential transcriptional targets, we performed RNA sequencing on FACS‐isolated macrophages from wild‐type and *atos^PBG^* embryos during early germband invasion (Fig EV3A, Source data for Fig 3). Twenty‐five genes displayed reduced mRNA levels and 39 higher ones in the absence of Atos with a *P* < 0.05 (Fig EV3B). Gene ontology analysis (GO term) indicates that the significantly downregulated genes affect oxidation‐reduction (redox), stress responses, as well as the nervous system (Fig EV3C). We focused on the five genes that had at least a > 5‐fold decrease in expression, and were enriched in embryonic macrophages or had an identified molecular function (Fig 3A). Expressing RNAi constructs in macrophages against three of these produced a significant reduction in macrophage numbers within the germband (Fig 3B–G), along with an increase on the yolk next to the germband, consistent with a specific defect in germband entry (Fig EV3D–F). These were a DEAD‐box (Ddx) protein (CG9253) we name Porthos (Pths) (preprint: Martin *et al*, 2021) (Fig 3B and E), and two metabolic enzymes, Glyoxylate Reductase/Hydroxypyruvate Reductase (GR/HPR, CG9331) (Fig 3C and F) and Lysine α‐Ketoglutarate Reductase/Saccharopine Dehydrogenase (LKR/SDH, CG7144) (Fig 3E and G). RNAis against the two others (Fig EV3G and H) produced no invasion defect. Porthos’ yeast and human orthologs are required for ribosomal RNA processing (O'Day *et al*, 1996; Sekiguchi *et al*, 2006). GR/HPR is highly conserved from bacteria to mammals, and the *Drosophila* form shows 48% identity to its human ortholog (NCBI BLAST). GR/HPR catalyzes the reduction of glyoxylate into glycolate and the conversion of hydroxypyruvate into D‐glycerate (Fig 3H) (Booth *et al*, 2006), contributing to glucose and urea synthesis. dLKR/SDH shows 71% identity to its human counterpart (identified by NCBI BLAST), catalyzing the first two steps of lysine catabolism and thus aiding acetyl CoA production (Fig 3I) (Bhattacharjee, 1985). We therefore conclude that Atos specifically enhances macrophage tissue invasion by increasing the levels of the metabolic enzymes dLKR/SDH and dGR/HPR and the Ddx protein Pths.

### The Ddx protein, Pths, functions downstream of Atos to promote pioneer macrophage germband invasion

**Figure EV4:**
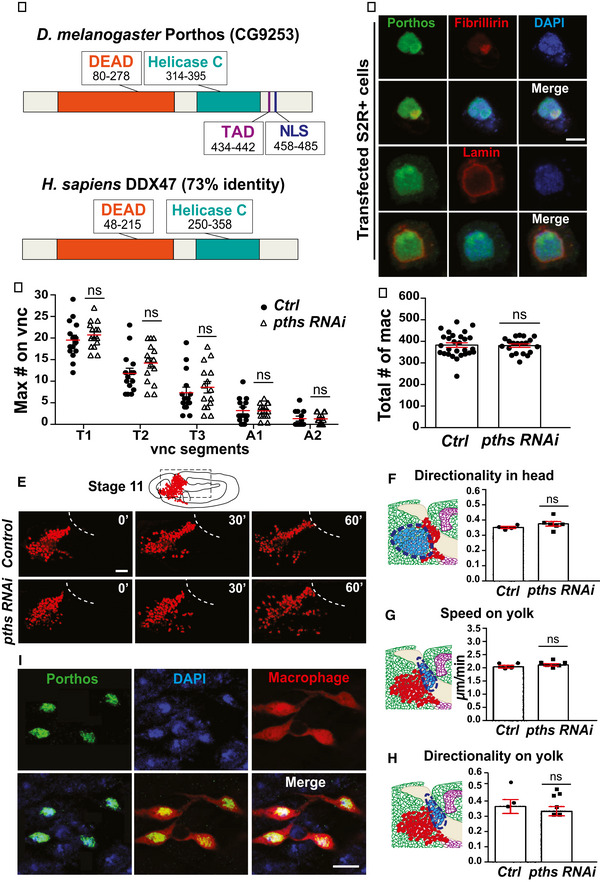
Downregulation of *porthos* recapitulates the *atos* mutant phenotype ADeduced protein structure of Porthos (CG9253). Porthos contains two conserved motifs, a DEAD motif (Asp‐Glu‐Ala‐Asp) and a Helicase C domain, as well as a predicted transactivation domain (TAD). *Drosophila* Porthos shows 71% identity and 84% similarity to its human ortholog, DDX47.BPorthos (green) in S2R^+^ cells transfected with *UAS‐pths::HA* and *srpHemo‐Gal4* and stained for the nuclear membrane marker Lamin (red), colocalizes with the staining for the nucleolar marker Fibrillarin (red), and DAPI (blue).C, DQuantification of macrophage numbers in fixed Stage 12 embryos. Expression of *porthos* (*pths*) *RNAi* in macrophages has no effect in their numbers on (C) the vnc or (D) in the whole embryo compared to the control. For (C) control *n* = 15 embryos, *pths RNAi n* = 15, *P* > 0.35. For (D) control *n* = 28 embryos, *pths RNAi n* = 20, *P* = 0.85.EStills from two‐photon movies of the migration of macrophages labeled with *srpHemo‐H2A::3xmCherry* in control embryos and in those expressing *porthos RNAi* in macrophages.F–HMacrophages from both genotypes have a similar (F) directionality in the head, and (G) speed and (H) directionality on the yolk sac, to control macrophages. For (F) directionality in head: control = 0.35, *pths RNAi* = 0.37; *P* = 0.27; control *n* = 4 movies, *pths RNAi n* = 6. For (G) speed on yolk sac: control = 2.10 µm/min, *pths RNAi* = 2.15; *P* = 0.35; control *n* = 4 movies, *pths RNAi n* = 6; control *n* = 104 tracks, *pths RNAi n* = 168. For (H) directionality on yolk: control = 0.42, *pths RNAi* = 0.39; *P* = 0.58; control *n* = 3 movies, *pths RNAi n* = 6.IMacrophages (cytoplasm, red) expressing pths::FLAG::HA near the germband in Stage 11/12 *atos^PBG^* embryos show partial colocalization of the HA‐antibody labeling Pths (green) with the nucleus (DAPI, blue). Pths expressed under *srpHemo‐GAL4 UAS* control. Data information: Mean ± SEM, ns=*P* > 0.05, Unpaired *t*‐test for (C‐D), and (F‐H). Scale bars: 5 µm (B), 30 µm (E), and 50 µm (I). See Source Data 1 and 2 for Fig EV4. Source data are available online for this figure.

**Figure 4:**
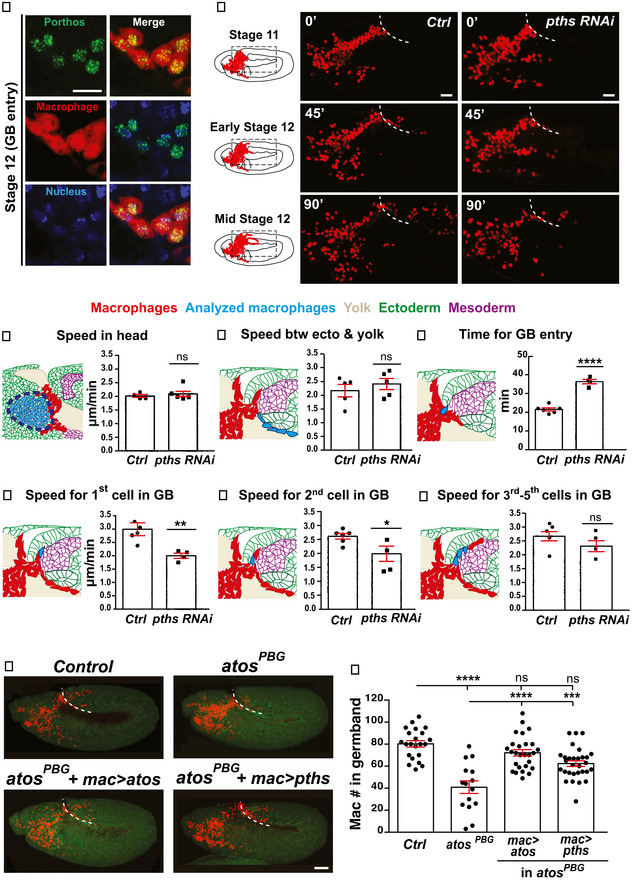
The nucleolar Ddx protein, Pths, acts as a key downstream target of Atos to promote pioneer macrophage germband invasion AMacrophages (cytoplasm, red) near the germband in Stage 11/12 embryos show partial colocalization of the HA‐antibody labeling Porthos (green) with the nucleus (DAPI, blue).BStills starting at Stage 11 from two‐photon movies of control embryos and those expressing *porthos (pths)‐RNAi* in macrophages (nuclei, red), migrating from the head mesoderm toward and into the germband at the indicated time points. White dotted line: germband edge.C–HQuantification of macrophage migration parameters from movies as in (B). Macrophage migration speed (C) in the head or (D) between the yolk sac and the germband edge. (C) control *n* = 4 movies, *pths RNAi n* = 6; control *n* = 507 tracks, *pths RNAi n* = 859; *P* = 0.56. (D) control *n* = 5 movies, *pths RNAi n* = 5; control *n* = 40 tracks, *pths RNAi n* = 51; *P* = 0.45. (E) The time required for the first macrophage nucleus to enter into the extended germband: control = 23 min, *CG9005^PBG^* = 38 min, *P* < 0.0001. The migration speed of the (F) 1^st^, (G) 2^nd^, or (H) 3^rd^‐5^th^ macrophages along the first 25–30 µm into the germband between the mesoderm and ectoderm: control = 21.5 min, *n* = 6, *pths RNAi* = 36.2 min, *n* = 4, *P* < 0.0001.IConfocal images of early Stage 12 embryos from control, *atos^PBG^*, and *atos^PBG^* expressing *atos::FLAG::HA* (*mac>atos*) or *pths::FLAG::HA (mac>pths)* in macrophages (red). Embryo detected by phalloidin staining (green). White dotted line: germband edge.JQuantification of macrophages in the germband of an *atos^PBG^* mutant rescued by expressing *pths::FLAG::HA* in macrophages. For control (*n* = 15 embryos) versus *atos^PBG^* mutant (*n* = 22) and *mac>atos* rescue (*n* = 27) both *P* < 0.0001, *versus mac>pths* rescue (*n* = 30) *P* = 0.0007. Data information: Movies in each analysis set are from independent embryos. Mean ± SEM, ns = *P* > 0.05, *P < 0.05, **P < 0.01, ***P < 0.001, ****P < 0.0001. Unpaired *t*‐test (C‐H); one‐way ANOVA with Tukey (J). Scale bars: 50 µm (A,I), 30 µm (B). See Source Data 1 for Fig 4. Source data are available online for this figure.

Pths is a member of the conserved DEAD‐box family (Fig EV4A), 71% identical and 84% similar to its human ortholog, DDX47, and shares the conserved DEAD‐motif and domain which interacts with RNA structures. *pths* (CG9253) mRNA is expressed in the embryo by *in situ* analysis in a pattern similar to *atos* (CG9005) mRNA but a few stages later, being enriched in macrophages during Stages 9–12 (BDGP *in situ* of porthos (CG9253) mRNA). In S2R^+^ cells, HA‐tagged Pths colocalized with markers for the nucleus (DAPI) and the nucleolus (Fibrillarin), where ribosome assembly and rRNA processing occur (Fig EV4B). In embryonic macrophages, HA‐tagged Pths also localized to the nucleus (Fig 4A). We examined macrophages in control embryos and those expressing *pths RNAi*. In fixed embryos, we observed no change in their migration along the noninvasive route of the vnc (Fig EV4C) or in their total number compared to the control (Fig EV4D), arguing that Pths is specifically required for migration into or within the tissues of the germband. 2‐Photon imaging and macrophage tracking in live embryos (Movies EV3 and EV4, Fig EV4E) detected no significant change in speed or directionality in the head, on the yolk or beneath the germband (Figs 4C and D, and EV4F–H) (Speed: in head 2 µm/min for control and *pths RNAi*, *P* = 0.56; on yolk, control = 2.1, *pths RNAi* = 2.2 µm/min, *P* = 0.35; beneath germband, control = 2.2, *pths RNAi* = 2.4 µm/min, *P* = 0.45. Directionality: in head, control = 0.35, *pths RNAi* = 0.37, *P* = 0.27; on yolk, control = 0.42, *pths RNAi* = 0.39, *P* = 0.58). However, as in the *atos* knockdown, *pths RNAi* macrophages waited 69% longer than the control to enter the germband tissue (control = 21.5, *pths RNAi* = 36.3 min, *P* < 0.0001) (Fig 4B and E). Once within the germband, the first two macrophages progressed significantly slower than the control (Fig 4F and G) (1^st^ cell: control = 3.0, *pths RNAi* = 2.0 µm/min, *P* = 0.009, 2^nd^ cell: control = 2.6, *pths RNAi* = 2.0 µm/min, *P* = 0.037). In contrast, the speed of the subsequent macrophages was not significantly altered by *pths RNAi* (Fig 4H) (3^rd^‐5^th^ cells: control = 2.7, *pths RNAi* = 2.3 µm/min *P* = 0.21). Thus, *pths RNAi* phenocopies *atos*’s migration defect. Finally, we expressed Pths in *atos^PBG^* to restore its higher levels in macrophages (Fig EV4I). This strongly improves the *atos* mutant phenotype (87% rescue) (Fig 4I and J). We conclude that Pths is a key player downstream of Atos, exerting an essential role in pioneer macrophages to specifically allow their initiation of germband invasion.

### Pths alters translation of a subset of mRNAs

**Figure 5:**
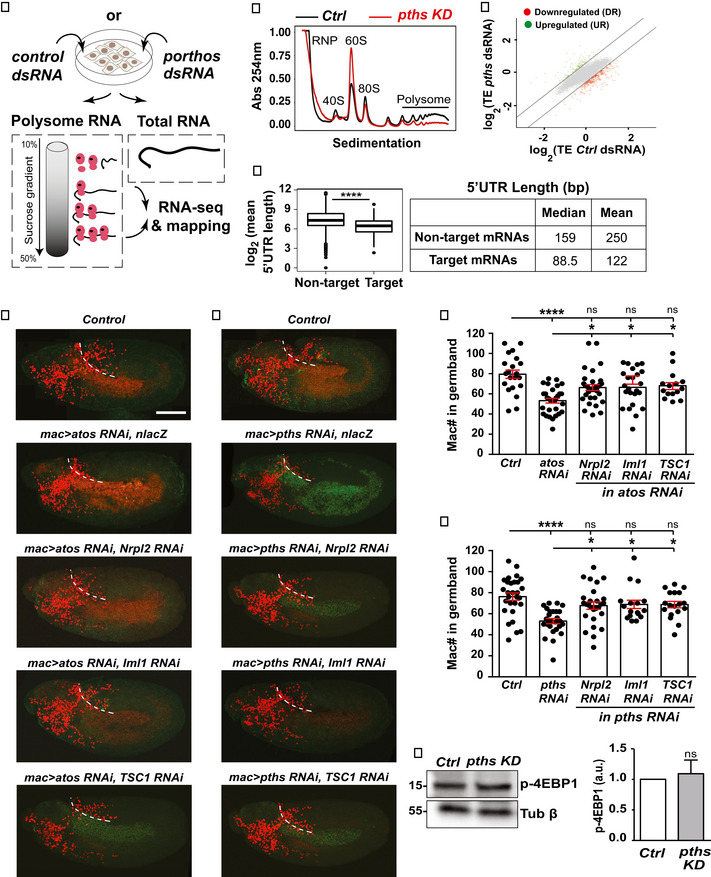
Pths increases assembled ribosome levels to stimulate the translation of an mRNA subset with short 5′UTRs ASucrose density gradient fractionation allowed purification of ribosome subunits and polysomes. Polysomal or total cellular mRNA fractions were isolated following dsRNA treatment and RNA sequencing libraries were prepared.BSedimentation analysis showing the relative abundance of 40S, 60S, and 80S ribosomes in cells treated with a *gfp‐dsRNA* (control) or *porthos (pths)*‐*dsRNA*. Profiles were aligned by the 40S ribosome peak’s position. Black: control, Red: *pths*‐*KD*. *n* = 3 independent biological replicates.CScatter plot of translational efficiency (TE) from *pths‐dsRNA* S2R^+^ versus control *gfp‐dsRNA* cells. Red (downregulated, DR) and green (upregulated, UP) dots represent genes with log_2_TE changes that meet the 2 standard deviation cutoff.DBioinformatic analysis shows that mRNAs whose TE is increased by Porthos have significantly shorter 5′UTRs than non‐targets (*P* = 1.69e‐35). Box and whisker plot of log2(Mean 5′UTR length) of each genotype. Middle line of box represents median; upper and lower bounds of box represent 1st and 3rd quartiles of data. Whiskers represent 1.5*IQR (inner quartile range), points outside of 1.5*IQR are plotted explicitly. The corresponding table shows median/mean 5′UTR lengths in base pairs (bp) for all mRNAs expressed in S2 cells (non‐targets) and for the subset whose TE is enhanced by Porthos (Porthos targets). Targets were defined using *n* = 3 biological replicates of polysome‐seq. 5′UTR length comparison is between *n* = 8,451 non‐target genes and *n* = 204 Porthos targets.E, FConfocal images of early Stage 12 embryos from the controls, from lines expressing nlacZ and RNAis against *atos* or *pths* in macrophages (mac>) as a second control, and lines expressing RNAis targeting *atos* or *pths* along with individual components of the dTORC1 pathway in macrophages, including *Nrpl2*, *Iml1*, and *TSC1*. Germband edge: dotted white line.G, HQuantification of the number of germband macrophages in embryos from the controls and upon RNAi knockdown of the genes in E‐F. Expression of different RNAis against *Nrpl2*, *Iml1*, and *TSC1* in *atos* or *pths*‐depleted macrophages rescues macrophage invasion into the germband while expression of lacZ does not. In (G) control (*n* = 21 embryos) versus *atos RNAi* (*n* = 30) *P* < 0.0001; control versus *atos RNAi* rescued with *Nrpl2 RNAi* (*n* = 32) *P* = 0.019, with *Iml1 RNAi* (*n* = 24) *P* = 0.03, and with *TSC1 RNAi* (*n* = 15) *P* = 0.04. In (H) control (*n* = 29 embryos) versus *pths RNAi* (*n* = 26) *P* < 0.0001; control versus *pths RNAi* rescued with *Nrpl2 RNAi* (*n* = 27) *P* = 0.01, with *Iml1 RNAi* (*n* = 17) *P* = 0.02, and with *TSC1 RNAi* (*n* = 18) *P* = 0.02.IWestern blot of protein extracts from control and *pths KD* S2R^+^ probed with p‐4EBP1 antibody. Tubulin serves as a loading control and quantification normalized to loading control. *N* = 3 biological replicates for both control and *pths KD*, *P* = 0.69. Data information: Mean ± SEM, ns = *P* > 0.05, *P < 0.05, ****P < 0.0001. Welch's *t*‐test (D), unpaired *t*‐test (I), one‐way ANOVA with Tukey (G‐H). Scale bar: 30 µm (E‐F). See Source Data 1 and 2 for Fig 5. Source data are available online for this figure.

Given Pths’s nucleolar localization and domains that are known to interact with RNA, we hypothesized that it might modulate translation. We conducted sucrose density gradient fractionation of S2R^+^ cells (Fig 5A) and observed a reduction in polysomes, the 40S small subunit, and 80S ribosome fraction (Fig 5B) along with an increase in the large 60S subunit peak in cells treated with *pths‐dsRNA* compared to the control. These data support the idea that the higher levels of Pths triggered by Atos could affect mRNA translation.

To identify mRNA transcripts dependent on Pths for their efficient translation, we performed polysome‐profiling, sequencing transcripts associated with highly translationally active polysomes, as well as all the transcripts in the S2R^+^ cells (Fig 5A and B). We calculated translational efficiency (TE) as the ratio of the normalized reads present for each gene in the mRNAs from the polysome fraction to those in the total mRNA sample; this ratio was determined for the data from both the control *GFP‐dsRNA* and *pths‐dsRNA* cells. We plotted the mean TE values for control and *pths‐dsRNA* replicates and calculated the mean change in TE (ΔTE) for each gene as the ratio of TEs between control and *pths*‐*dsRNA* replicates (Fig 5C). Targets were defined as genes falling 2 standard deviations from the median ΔTE as previously described (Flora *et al*, 2018). We identified 204 annotated coding genes that were less efficiently translated and 102 that were more efficiently translated in *pths*‐*dsRNA* cells (Source data for Fig 5).

An unbiased search revealed no sequence motifs statistically enriched in the 5′ untranslated regions (5′UTRs) of mRNAs dependent on Pths for their polysomal enrichment, neither TOP motifs (Meyuhas, 2000; preprint: Martin *et al*, 2021) nor TISU elements (Elfakess & Dikstein, 2008). Transcripts with short 5′UTRs in human blood cells are less well translated under ribosome limiting conditions (Mills & Green, 2017; Khajuria *et al*, 2018). We thus analyzed the length of the 5′UTRs of mRNAs that require Pths for enhanced TE in S2 cells and found that they are significantly shorter than nontarget mRNAs (Fig 5D) (*P* = 1.69e‐35). Our data suggest that Pths leads to sufficient amounts of assembled ribosomes to help efficiently translate mRNA targets with shorter 5′UTRs.

We wished to further investigate if Pths affects invasion through changes in ribosomal assembly and translation. The first step for gaining access to the nucleolus where ribosome assembly occurs is nuclear entry. To examine if this is crucial for Pths function, we made a form lacking the NLS. We found FLAG::HA‐tagged Pths^nls‐^ mainly in the cytoplasm in macrophage‐like S2R^+^ cells (Appendix Fig S1A). This form showed a reduced capacity to rescue macrophage germband invasion in *atos^PBG^* embryos compared to those expressing wild‐type Pths (Appendix Fig S1B and C). Thus, Porthos’ nuclear localization is important to facilitate macrophage invasion.

Activation of the TORC1 pathway is known to increase ribosome biogenesis and translational initiation (Iadevaia *et al*, 2014; Liu & Sabatini, 2020). We reasoned that if the invasion defects caused by the absence of Pths were due to decreases in either of these cellular functions, activating the TORC1 pathway could overcome this problem. We depleted TORC1’s inhibitory regulators, including Nrpl2, Iml1, and TSC1, using RNAis (Fig 5E and F), which significantly rescued the germband invasion defect of *atos‐KD* or *pths‐KD* macrophages (Fig 5G and H). We then assessed if Pths might regulate the TORC1 pathway itself, using specific antibodies against the phosphorylated form of 4E‐BP1. We observed no significant change in p‐4EBP1 levels in *pths*‐*KD* cells compared to the control (*P* = 0.69) (Fig 5I). In sum, our data support the conclusion that Pths does not affect the phosphorylation state of the TORC1 target 4EBP1 and thus general translation, but acts through ribosome biogenesis and translation of a specific cohort of mRNAs.

### Nuclear Pths increases the levels of mitochondrial OxPhos complexes

**Figure 6:**
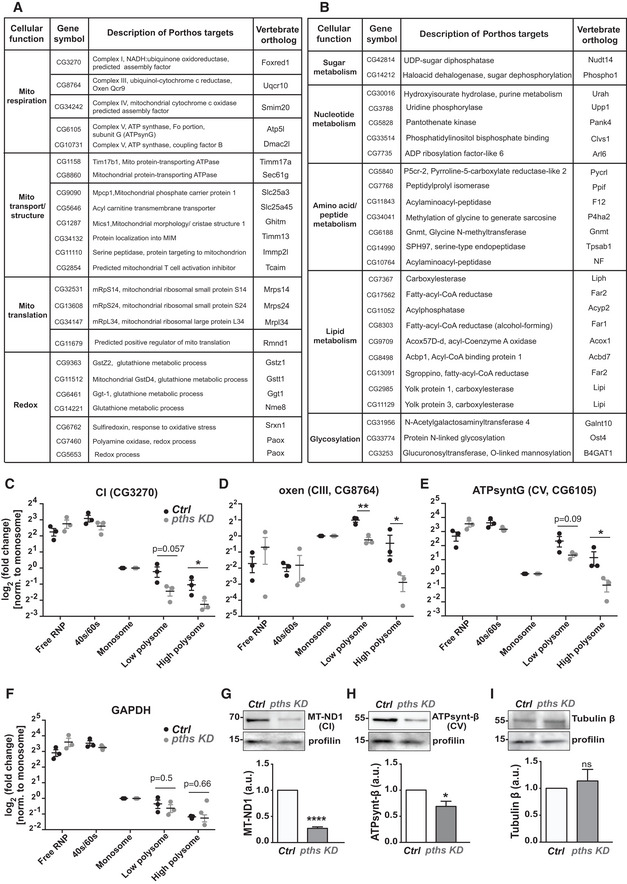
Pths increases the translational efficiency of nuclear‐encoded mitochondrial proteins A, BPths increases the translation of RNAs involved in (A) mitochondrial function and (B) metabolic pathways, along with transcription, translation, signal transduction, immune responses, as well as redox processes as shown in Appendix Fig S1. See also Dataset EV1.C–FRT‐qPCR analysis of mRNA from ribosomal protein fractions for control (black) and *pths KD* (gray) transcripts from S2R^+^ cells. RNA was isolated individually from fractions and pooled into five categories: RNP, 40S/60S, monosome, low polysome (di‐ and trisome), and high polysome (remaining fractions). The data were plotted relative to the amount present in the monosome fraction for each transcript. The mRNA levels of subunits of mitochondrial complexes (C) I, (D) III, and (E) V were reduced in low and high polysome fractions in *pths KD* cells compared to the control cells, while (F) mRNA levels of GAPDH in the same fractions, used as an internal control, were not changed (*n* = 3 independent biological replicates for control and *pths KD*). For (C,E‐F) low polysome fraction for control versus *pths KD*: (C) *P* = 0.057, (D) *P* = 0.0039, (E) *P* = 0.09, (F) *P* = 0.5. For (C,E‐F), high polysome fraction for control versus *pths KD*: (C) *P* = 0.039, (D) *P* = 0.046, (E) *P* = 0.041, (F) *P* = 0.66.G–IWestern blots and their quantifications of protein extracts from control and *pths KD* S2R^+^ probed with (G) MT‐ND1 (for mitochondrial complex I) *n* = 4, *P* < 0.0001, (H) ATPsynt‐β (for mitochondrial complex V) *n* = 3 *P* = 0.034, and (I) tubulin β antibodies *n = 3* *P* = 0.56. Profilin serves as loading control. *N*=biological replicates. Data information: Mean ± SEM, ns = *P* > 0.05, *P < 0.05, **P < 0.01, ****P < 0.0001. Unpaired *t*‐tests (C‐F, G‐I). See Source Data 1 and 2 for Fig 6, Appendix Fig S1. Source data are available online for this figure.

The mRNA targets whose TE Pths enhanced in the RNA sequencing analysis of polysomes are involved in respiration, transport, and translation in mitochondria, metabolic processes, transcription, translation, signal transduction, immune responses, as well as redox processes (Fig 6A and B, Appendix Fig S2A–C). The targets include several components of mitochondrial OxPhos, namely ubiquinol cytochrome C reductase (complex III, UQCR‐Q), ATP synthase subunit G and coupling factor F(o) (complex V), predicted assembly factors for complex I and IV, and proteins involved in mitochondrial translation and transport (Fig 6A) as well as other metabolic pathways (Fig 6B).

To validate the results further, we conducted RT‐qPCR across the fractions in the sucrose gradient of control and *pths*‐KD S2R^+^ cell extracts for a number of transcripts. We examined five different polysome profile fractions: the RNP, 40S/60S, monosome, low polysome (di‐ and trisome), and high polysome (remaining fractions) (Fig 5B). Given that our data had shown that, along with Pths, Atossa increases mRNA levels of two metabolic enzymes upstream of substrates for mitochondrial OxPhos, we focused on components of this energy producing process. The data were plotted relative to the amount present in the monosome fraction for each transcript. We observed that the mRNA levels detected by RT‐qPCR on high polysome fractions for subunits of mitochondrial OxPhos complexes I, III, and V (Fig 6C–E) were reduced in *pths*‐*KD* S2R^+^ cells compared to the control (*P* = 0.039, 0.046 and 0.041, respectively). GAPDH which had shown no reduction upon *pths*‐KD in occupancy on polysomes in the RNA seq analysis displayed none by RT‐qPCR as well (*P* = 0.66) (Fig 6F). These data confirm that Pths shifts the mRNAs of multiple OxPhos components into a higher gear of translation.

To determine if the reduced occupancy of mRNAs encoding mitochondrial proteins on the polysomes resulted in reductions in protein levels, we performed western blots on extracts from control and *porthos‐KD* S2R^+^ cells. We were unable to obtain antibodies corresponding to the targets identified in the RNA sequencing. However, lower levels of the mammalian ortholog of the predicted complex I assembly factor we identified as a target in the RNA sequencing lead to reduced levels of other complex I proteins (Formosa *et al*, 2015), including MT‐ND1 for which there is an available antibody. Similarly, in humans, the absence of subunit g of complex V, one of our targets, has been shown to lead to lower protein levels of multiple other subunits including ATP synt‐β (He *et al*, 2018) for which we could obtain an antibody. Western blots reveal 73 and 31% lower levels of these CI and CV proteins, respectively, in *pths‐KD* S2R^+^ cells compared to the control (CI MT‐ND1, *P* < 0.0001; CV ATP synt‐β, *P* = 0.03) (Fig 6G and H). To test for a possible general deficiency in protein translation, we examined the nontarget proteins profilin and tubulin β and found no significant change in levels (Fig 6G–I) (profilin, *P* = 0.26; β tubulin, *P* = 0.55). In sum, our results argue that Pths does not affect protein translation generally, but is required for the enhanced levels of a subset of proteins, many of which are involved in mitochondrial and metabolic function.

Mitochondrial protein levels can also be regulated by CLUH and PGC‐1; CLUH enhances the stability and translation of mRNAs encoding mitochondrial proteins (Schatton *et al*, 2017; Pla‐Martín *et al*, 2020) and PGC‐1 stimulates transcription of components required for mitochondrial biogenesis (Lin *et al*, 2005), as does its *Drosophila* ortholog, Spargel (Tiefenböck *et al*, 2010). To examine if Atossa might coordinate with either of these two pathways, we examined the overlap in targets. We found only five proteins from various CLUH target datasets whose orthologs overlapped with Pths targets which did not reach significance (*P* = 0.59, *P* = 0.20). However, *Drosophila* PGC‐1, Spargel, and Pths did display significant overlap (*P* < 0.036), with 12 shared targets (Appendix Table S1). Spargel mRNA also shows a similar expression pattern in the BDGP *in situ* database to that of Atos and Pths during the time of macrophage invasion (BDGP *in situ* of spargel mRNA); it is found in the midgut, salivary gland, and macrophages, which we confirmed also in our RNA seq analysis from sorted macrophages (Source Data 2 for Fig 3). Thus, Atossa and PGC‐1 may synergize to stimulate mitochondrial function, with PGC‐1 increasing the transcription of proteins whose translation Atossa then enhances.

### Pths is required for mitochondrial oxidative respiration and energy production

**Figure EV5:**
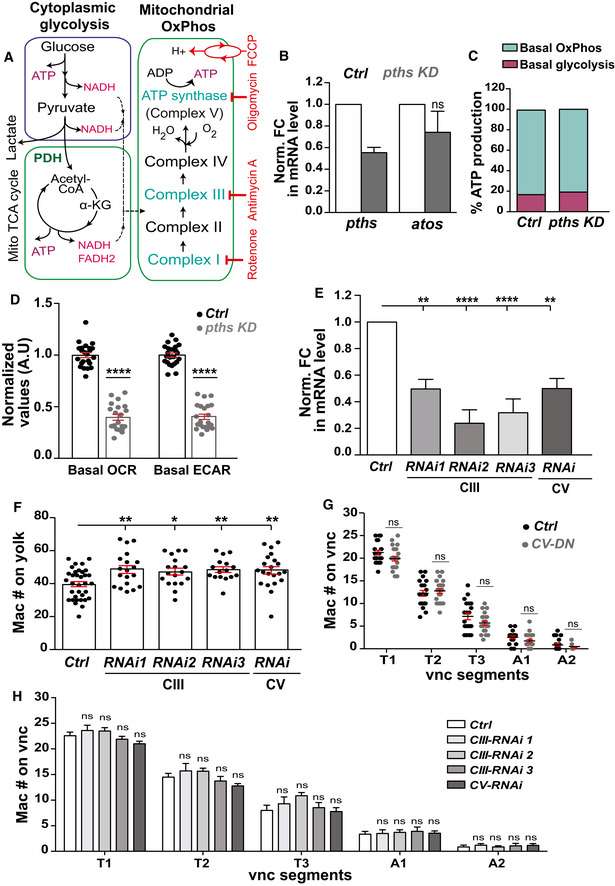
Depletion of Atossa or Porthos mRNA causes impairment in mitochondrial metabolic activity, reduced ATP production, and a deficiency in macrophage tissue invasion ASchematic indicating the specific inhibitors (in red at right) used to block the function of mitochondrial OxPhos components. The glycolysis, TCA cycle, and mitochondrial respiratory chain in eukaryotic cells are shown.BGraph shows relative *porthos* (*pths*) and *atos* mRNA levels in *pths KD* S2R^+^ cells measured by qPCR from at least three independent experiments. The data are normalized to results for the internal control gene RpS20. *pths KD* S2R^+^ cells contain 56% of normal *pths* mRNA levels and display a statistically insignificant decrease in *atos* mRNA levels. Control (*n* = 6 biological replicates) versus *pths* (*n* = 6), *P* = 0.0002, versus *atos* (*n* = 3), *P* = 0.09.CThe contribution of basal OxPhos ATP production rate and glycolytic ATP production rate were calculated. The plot shows that both wild‐type and *pths KD* S2R^+^ cells utilize OxPhos respiration as the predominant bioenergetic pathway to produce ATP in these cells; we observe no increase in the relative utilization of glycolysis.DThe relative basal values of the Oxygen Consumption rate (OCR), as a marker of OxPhos, and Extracellular Acidification Rate (ECAR), as an indication of glycolysis, in control and *pths KD* S2R^+^ cells are plotted. Basal respiration rate is calculated before the addition of antimycin A. For OCR and ECAR assay, analysis is from values obtained in *n* ≥ 3 independent biological experiments each with *n* > 6 technical replicate. Control versus *pths KD* for both assays: *P* < 0.0001.EGraph shows mRNA levels relative to the control of the targeted gene in embryos ubiquitously expressing RNAis against subunits of mitochondrial Complexes III and V. Expression measured by qPCR and normalized to results for the internal control gene RpS20; *n* = 3 independent biological experiments. RNAi KD of *Complex III* resulted in 49% (RNAi 1), 24% (RNAi 2), and 32% (RNAi 3) of its normal mRNA levels; for *Complex V*, this was 50%. Control versus *Complex III RNAi 1*, *P* = 0.0048; versus *Complex III RNAi 2 P* = 0.0002; versus *Complex III RNAi 3 P* = 0.0005; versus *Complex V RNAi P* = 0.005FQuantification in fixed early Stage 12 embryos shows a significant increase of macrophages on the yolk upon the expression in macrophages of any of three different *RNAis* against mitochondrial OxPhos *Complex III* (*UQCR*) or an *RNAi* against *Complex V* (*F1F0*, CG3612). For control (*n* = 34 embryos) versus *Complex III* (*Cyt‐c1*, CG4769) *RNAi* 1 (VDRC 109809, *n* = 19) *P* = 0.0049; versus *Complex III* (*UQCR‐cp1*, CG3731) *RNAi* 2 (VDRC 101350, *n* = 18) *P* = 0.024; versus *Complex III* (*UQCR‐cp2*, CG4169) *RNAi* 3 (VDRC 100818, *n* = 16) *P* = 0.009; versus *Complex V* (*F1F0*, *CG3612*) *RNAi* (VDRC 34664, *n* = 21) *P* = 0.0068.G, HQuantification of the number of macrophages in vnc segments does not show a significant change in general migration along the vnc in embryos whose macrophages express (G) *CV‐DN* or (H) *RNAis* against mitochondrial OxPhos complex components compared to the control. For (G) control (*n* = 20 embryos) versus *CV‐DN* (*n* = 23) *P* > 0.05. For (H) control (*n* = 14 embryos) versus *Complex III (Cyt‐c1*, CG4769) *RNAi* 1 (VDRC 109809, *n* = 10) *P* > 0.8; versus *Complex III* (*UQCR‐cp1*, CG3731) *RNAi* 2 (VDRC 101350, *n* = 14) *P* > 0.05; versus *Complex III* (*UQCR‐cp2*, CG4169) *RNAi* 3 (VDRC 100818, *n* = 11) *P* > 0.9; versus *Complex V* (*F1F0*, CG3612) *RNAi* (VDRC 34664, *n* = 18) *P* > 0.2. Data information: Ubiquitous expression of RNAis is through *da‐GAL4*. Mean ± SEM, ns=*P* > 0.05, *P < 0.05, **P < 0.01, ***P < 0.001, ****P < 0.0001. Unpaired *t*‐test followed by Sidak's correction (B,E). Unpaired *t*‐test for (D‐H). See Source Data 1 for Fig EV5. Source data are available online for this figure.

**Figure 7:**
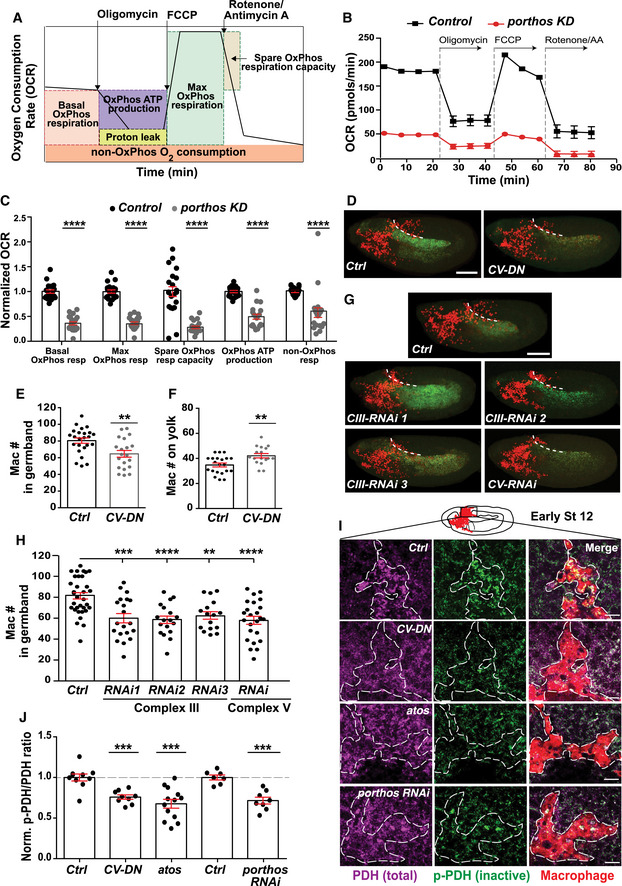
Porthos boosts mitochondrial respiration, which is required in macrophages to power their germband tissue invasion ASchematic of mitochondrial energetic profiling with a Seahorse efflux assay.BThe Oxygen Consumption Rate (OCR, pmols O_2_/min) assessed as a representative parameter of OxPhos in control and *pths*‐*KD* S2R^+^ cells by a Seahorse efflux assay. *n* ≥ 3 independent biological experiments each with *n* > 6 technical replicate.CDistinct respiration parameters calculated from relative OCR values obtained in experiments shown in B. *P* < 0.0001 for all comparisons.D–F(D) Confocal images and (E) quantification of macrophages in the germband or (F) on the yolk next to the germband from control embryos and those expressing a dominant negative c‐ring of ATP synthase (CV‐DN) in macrophages. (D‐H) Images show Stage 12 embryos and germband edge with dotted white line. For (E) control (*n* = 24 embryos) versus CV‐DN (*n* = 20) *P* = 0.0032. For (F) control (*n* = 21 embryos) versus CV‐DN (*n* = 17) *P* = 0.003.G, H(G) Confocal images and (H) quantification of germband macrophages in control embryos and those expressing different *RNAis* against mitochondrial OxPhos *Complex III (*or an *RNAi* against *Complex V*. For (H) control (*n* = 34 embryos) versus *Complex III RNAi* 1 (*n* = 20) *P* = 0.0001; versus *Complex III RNAi* 2 (*n* = 18) *P* = 0.027; versus *Complex III RNAi* 3 (*n* = 16) *P* < 0.0001; versus *Complex V RNAi* (*n* = 14) *P* < 0.0001.ISingle plane confocal microscope image during germband entry from control or *atos^PBG^* embryos, or those expressing *pths*‐*RNAi* or *CV‐DN* in macrophages. Antibodies against S293‐phosphorylated inactivated Pyruvate Dehydrogenase (pPDH, green) or total PDH (magenta) in macrophages (red). Higher pPDH levels are usually found when ATP/ADP levels are high and input into the TCA cycle is being downregulated (Patel *et al*, 2014).JQuantification of normalized pPDH/PDH levels calculated from fluorescence intensities in macrophages from the genotypes in (I) during initial germband invasion. The pPDH/PDH ratio is significantly reduced, arguing that decreased function of CV, Atos or Pths in macrophages results in lower cellular ATP/ADP ratios compared to the control. *N* = 3 independent experiments. Ctrl 1 (*n* = 10 embryos) versus *CV‐DN* (*n* = 9) and versus *atos* mutant (*n* = 13) both *P* = 0.0002; Ctrl 2 (*n* = 7 embryos) versus *pths RNAi* (*n* = 8) *P* = 0.0001. *** shown above columns in J are for comparison to relevant control. Data information: D‐I show Stage 12 embryos. G‐H *Complex III* RNAis target Cyt‐c1, UQCR‐cp1‐2, for *Complex V*, ATP synthase F1F0. Scale bars: 50 µm (D,G) 10 µm (I). Mean ± SEM, **P < 0.01, ***P < 0.001, ****P < 0.0001. Unpaired *t*‐tests (B,E,J), two‐way ANOVA with Sidak's test (C), and one‐way ANOVA with Tukey’s (H). See Source Data 1 for Fig 7. Source data are available online for this figure.

Mitochondria generate ATP through OxPhos mostly from the pyruvate formed by the glycolytic pathway (Berg *et al*, 2002) (Fig EV5A) and thus can utilize metabolites downstream of the two enzymes we identified as Atos targets, LKR/SDH and GR/HPR. Given that Atos upregulates these three targets together to spur invasion, we hypothesized that Pths regulates mitochondrial energy production. We generated S2R^+^ cells producing 56% of *pths’s* normal mRNA levels with CRISPR/Cas9‐mediated mutagenesis (*pths‐KD* cells) (Fig EV5B) and utilized a Seahorse assay to determine the oxygen consumption rate (OCR) (Llufrio *et al*, 2018) before and after sequential treatment with compounds affecting different steps in OxPhos (Fig 7A and B). Comparing the OCR in different conditions, we calculated OxPhos‐dependent basal and maximum respiration and found that both were reduced 64% in *pths‐KD*. We saw a 72% reduction in OxPhos‐dependent spare respiration capacity and 42% in OxPhos‐independent respiration, perhaps due to reduced levels of the Pths target lysyl oxidase (Fig 7B and C, Appendix Fig S2B). S2R^+^ cells utilize primarily mitochondrial OxPhos rather than glycolysis for ATP production (Freije *et al*, 2012); this remains the case even in the *pths*‐*KD* cells (Fig EV5C) as we also observed a 60% reduction in the basal extracellular acidification rate (ECAR), a measure of lactate production through complete glycolysis (Fig EV5D). In totality, ATP production through OxPhos was reduced by 50% upon *pths* depletion (Fig 7C). Given that Pths modulates the translation of assembly factors for mitochondrial complex I and IV and accessory subunits of complex III and the ATP synthase complex V, our data argue that Pths normally increases metabolic capacity and flux to upregulate the OxPhos pathway and increase energy production.

### Mitochondrial respiration is required for metabolism and energy production in macrophages to initiate invasion into the germband tissue

We directly assessed the importance of the OxPhos complexes whose TE is upregulated by Pths for macrophage germband invasion in the embryo. We tested the effect of a dominant negative form of Complex V, the ATP synthase which converts the electron gradient produced during OxPhos into ATP (CV‐DN) (Hurd *et al*, 2016) (Fig 7D–F). We also expressed RNAis against different subunits of Complex III and the α‐subunit of Complex V in macrophages, reducing their mRNA’s expression by 50–80% (Figs 7G and H, and EV5E). Consistent with the polysome‐profiling results from *pths‐KD* S2R^+^ cells, these treatments significantly reduced macrophage numbers within the germband (Fig 7D, E, G and H) and increased them on the yolk at the germband entry site (Figs 7F and EV5F), phenocopying the germband invasion defect in *atos^PBG^* or *pths*‐*RNAi* in macrophages. We observed no significant difference in macrophage numbers on the vnc in late Stage 12 upon expression of *CV‐DN* or of the RNAis (Fig EV5G and H) compared to the control, indicating normal general migration. These data strongly support the conclusion that higher levels of the OxPhos complexes III and V are required specifically for macrophage tissue invasion.

### Atos and its target Pths increase macrophage bioenergetics for germband tissue invasion

To examine embryonic macrophages’ bioenergetics *in vivo* in the absence of Pths or Atos, we first assessed the pyruvate dehydrogenase complex (PDH), a key point of metabolic regulation (Patel *et al*, 2014) as it allows pyruvate formed by glycolysis to feed into the TCA cycle (Fig EV5A). Metabolites produced by the TCA cycle increase PDH’s phosphorylation thereby inhibiting it and the running of the cycle; metabolites utilized by the TCA cycle decrease PDH phosphorylation and activate it. Importantly, when energy levels fall and mitochondrial ADP levels rise, PDH is unphosphorylated and active, opening the gate to the TCA cycle and OxPhos (Patel *et al*, 2014). By antibody staining, we determined the levels of phosphorylated inactive PDH (pPDH) and the total amounts of PDH (Lieber *et al*, 2019) in embryonic macrophages. We assessed the pPDH/PDH ratio: a smaller number indicates less inhibition and thus more activity of PDH. As a positive control, we examined macrophages expressing CV‐DN, which blocks mitochondrial ATP synthase, and thus increases ADP levels. Indeed, we observed a lower pPDH/PDH ratio than in the control (Fig 7I and J). We also observed significantly lower pPDH/PDH ratios in macrophages invading the germband in *atos^PBG^* embryos as well as those expressing *pths* RNAi in macrophages compared to the control (Fig 7I and J). Our results support the conclusion that in the absence of Atos or Pths, macrophages *in vivo* have reduced ATP/ADP ratios, leading the cells to keep PDH in its active form to try to generate more ATP by converting pyruvate into acetyl CoA that can feed into the TCA cycle.

### Atos targets, Pths and the metabolic enzymes GR/HPR and LKR/SDH, enhance cellular bioenergetics to promote macrophage tissue invasion

**Figure 8:**
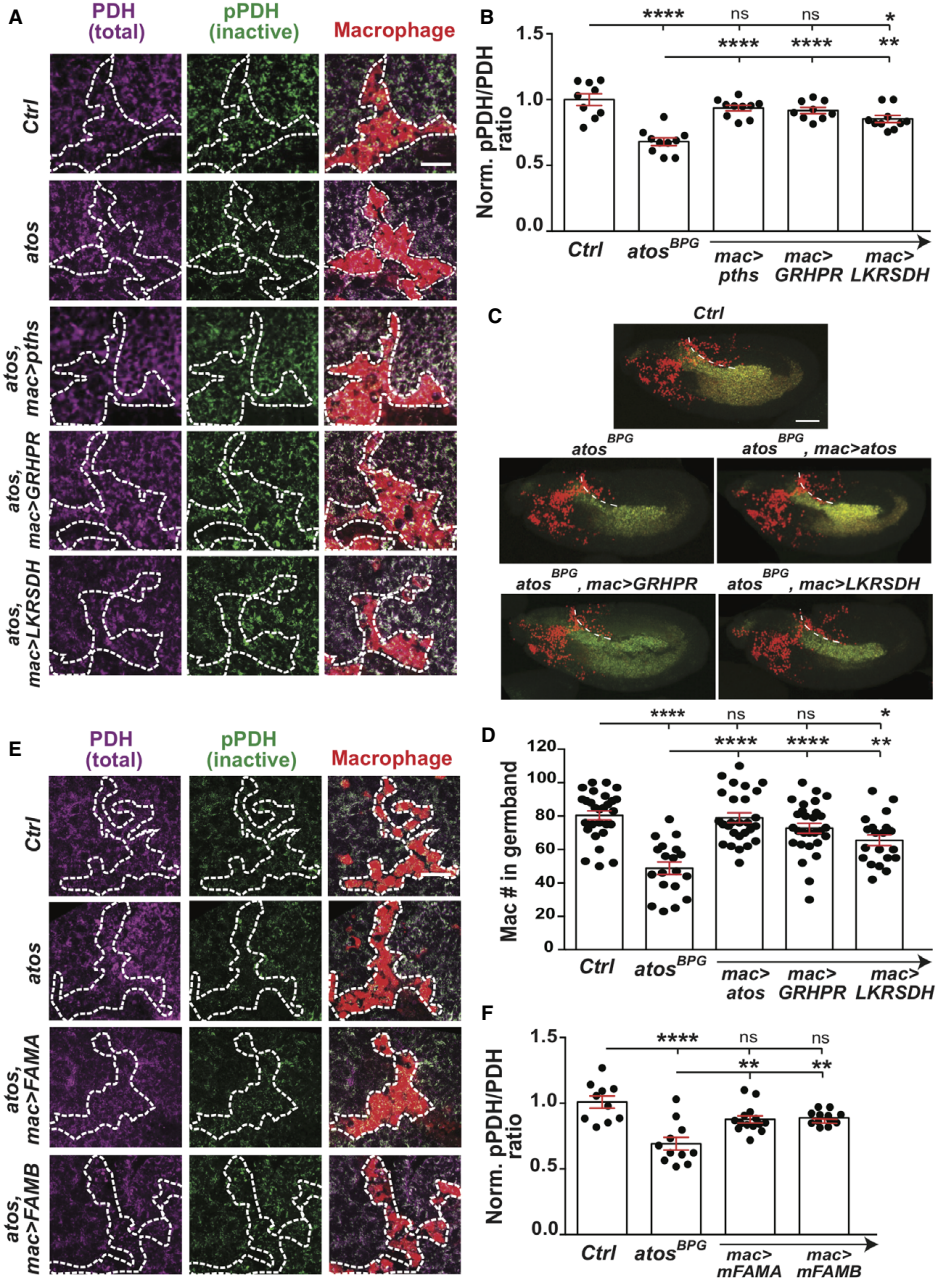
Atossa’s mammalian orthologs FAM214A‐B, and its targets Pths and the metabolic enzymes GR/HPR and LKR/SDH, are each sufficient to boost macrophage bioenergetics and germband invasion ASingle plane confocal microscope images during germband entry from control or *atos^PBG^* embryos, or those expressing *pths*, *GR/HPR*, and *LKR/SDH* in macrophages (*mac>*). Embryos were stained for antibodies against S293‐phosphorylated inactivated Pyruvate Dehydrogenase (pPDH, green) or total PDH (magenta) in macrophages (red). Higher pPDH/PDH ratios are consistent with higher ATP/ADP levels.BQuantification of normalized pPDH/PDH levels calculated from fluorescence intensities in macrophages from the genotypes in (A) during initial germband invasion. The pPDH/PDH ratio is significantly increased in *atos^PBG^* embryos expressing either *pths*, *GR/HPR*, or *LKR/SDH* in macrophages compared to the *atos^PBG^* embryos. This argues that the decreased Atos function in *atos^PBG^* macrophages, resulting in lower cellular ATP/ADP ratios, was restored by expressing either its targets or murine orthologs. *N* = 3 independent experiments. Control (*n* = 9 embryos) versus *atos* mutant (*n* = 10) *P* < 0.0001. Control versus *atos* mutant rescued with *mac>pths* (*n* = 10) *P* = 0.77; rescued with *mac>GRHPR* (*n* = 9) *P* = 0.48; rescued with *mac>LKRSDH* (*n* = 10) *P* = 0.012. For *atos* mutant versus *atos* rescued with *mac>pths* or *mac>GRHPR P* < 0.0001. versus *atos* rescued with *mac>LKRSDH P* = 0.0014.C, D(C) Confocal images or (D) quantification of the macrophages in germband in Stage 12 embryos from the control, *atos^PBG^*, and *atos^PBG^* expressing *atos* itself or *GR/HPR*, or *LKR/SDH* in macrophages. Germband edge: dotted white line. For (D) control (*n* = 29 embryos) versus *atos* mutant (*n* = 19) *P* < 0.0001. Control versus *atos* mutant rescued with *mac>atos* (*n* = 27 embryos) *P* = 0.99; rescued with *mac>GRHPR* (*n* = 28) *P* = 0.29; rescued with *mac>LKRSDH* (*n* = 20) *P* = 0.036. *atos* mutant versus *atos* rescued with *mac>atos* or *mac>GRHPR P* < 0.0001; rescued with *mac>LKRSDH P* = 0.013.ESingle plane confocal microscope images during germband entry from control or *atos^PBG^* embryos, or those expressing mammalian orthologs m*FAM214A* or *B* (FAMA‐B) in macrophages (*mac>*). Embryos were stained for antibodies against S293‐phosphorylated inactivated Pyruvate Dehydrogenase (pPDH, green) or total PDH (magenta) in macrophages (red).FQuantification of normalized pPDH/PDH levels measured from fluorescence intensities in macrophages from the genotypes in (E) during initial germband invasion. The pPDH/PDH ratio is significantly increased in *atos^PBG^* embryos expressing either mammalian ortholog m*FAM214A* or *B* in macrophages compared to the *atos^PBG^* embryos. Results from three independent experiments. Control (*n* = 10 embryos) versus *atos* mutant (*n* = 11) *P* < 0.0001. Control versus *atos* mutant rescued with *mac‐mFAMA* (*n* = 14 embryos) *P* = 0.06; rescued with *mac‐mFAMB* (*n* = 12) *P* = 0.13. *atos* mutant versus *atos* mutant rescued with *mac‐mFAMA P* = 0.0025 or *mac‐mFAMB P* = 0.0019. Data information: Mean ± SEM, ns=*P* > 0.05, *P < 0.05, **P < 0.01, ****P < 0.0001. One‐way ANOVA with Tukey (B,D,F). Scale bars: 10 µm (A,E), 50 µm (C). See Source Data 1 for Fig 8. Source data are available online for this figure.

Next we tested our hypothesis that Atossa’s downstream targets Pths and the metabolic enzymes GR/HPR and LKR/SDH all can boost the cellular energetics required for macrophage tissue invasion. We measured the pPDH/PDH ratios in macrophages invading the germband in *atos^PBG^* mutant embryos and in such mutants also expressing either Porthos, GR/HPR, or LKR/SDH in macrophages (Fig 8A and B). We observed significantly higher pPDH/PDH ratios in these *atos^PBG^* macrophages upon the expression of any of these three Atos targets (Fig 8A and B). To further confirm our conclusion, we overexpressed either GR/HPR or LKR/SDH in macrophages in *atos^PBG^* embryos and we observed that these enzymes were able to rescue the germband invasion defect (Fig 8C and D). We also observed higher pPDH/PDH ratios in *atos^PBG^* macrophages expressing Atossa’s mammalian orthologs mFAM214 A and B (Fig 8E and F), arguing that regulation of energy levels by the Atossa pathway is conserved. These results strongly suggest that each of the three targets of Atossa, Porthos, and the two metabolic enzymes GR/HPR and LKR/SDH increases cellular energetics and ATP generation to facilitate tissue invasion, a capacity also observed in Atossa’s mammalian orthologs which we rename AtosA and AtosB.

### Atos enhances cellular metabolism and ATP/ADP levels

**Figure 9:**
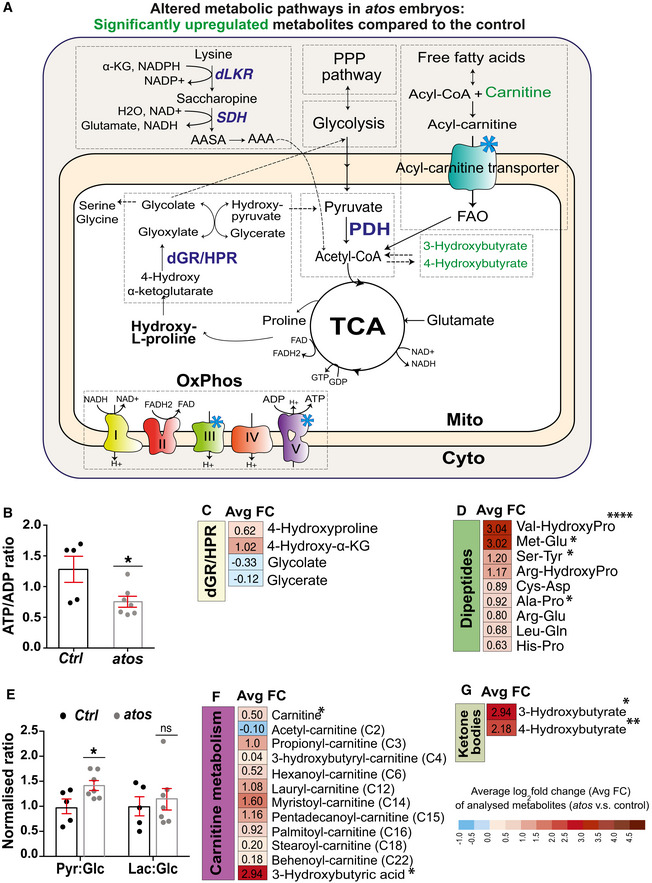
Mitochondrial metabolism is enhanced by Atos ASchematic depicting ATP‐generating pathways in eukaryotic cells: glycolysis, the Pentose Phosphate Pathway (PPP), fatty acid oxidation (FAO), the TCA cycle, and the mitochondrial respiratory electron transport chain (ETC). Blue stars mark *pths* targets. Green indicates individual metabolites with statistically significant upregulation in *atos^PBG^* compared to the control.B–GCellular metabolites were measured by LC–MS‐based untargeted metabolomics from extracts of Stage 11 embryos; biological replicates for control *n* = 5, for *atos^PBG^ n* = 7. (B) Normalized ATP/ADP ratio values are decreased in *atos^PBG^* compared to control embryos (*P* = 0.028). (C‐D,F‐G) Heatmap of non‐targeted metabolites in *atos^PBG^* compared to wild‐type embryos shown with average log_2_fold change (FC) in the non‐targeted LC‐MS/MS analysis reveals (C) an increase in *atos^PBG^* in substrates of the dGR/HPR enzyme, including 4‐hydroxy α‐ketoglutarate and hydroxyproline (HLP) and a smaller decrease in its products, glycolate and glycerate, (D) a significant increase in some dipeptides including those containing hydroxyproline. (E) Quantification shows an increase in *atos^PBG^* in the pyruvate/glucose ratio (*P* = 0.035), but none for the lactate/glucose ratio (*P* = 0.65). (F) We observe increases in *atos^PBG^* in intermediates of mitochondrial fatty acid β‐oxidation (FAO), including different carnitine‐conjugated lipids, and (F‐G) a significant increase in ketone body substituents compared to the control. Data information: Mean ± SEM, ns=*P* > 0.05, *P < 0.05, **P < 0.01, ****P < 0.0001. Values in heat maps are obtained from untargeted metabolomic analysis, unpaired *t*‐test (B‐G). See Source Data 1 for Fig 9 and Dataset EV1 for metabolomics source data. Source data are available online for this figure.

**Figure 10:**
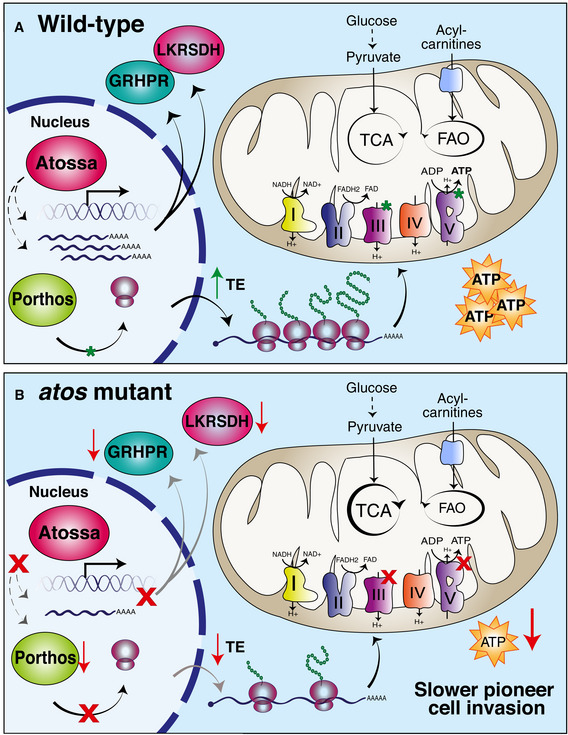
Atossa turns on a metabolic program to boost mitochondrial bioenergetics for macrophage tissue invasion The developmental upregulation of Atossa in macrophages increases mRNA levels of the nucleolar protein Pths and the metabolic enzymes GR/HPR and LKR/SDH. Metabolic pathways downstream of GR/HPR and LKR/SDH are known to produce metabolites that feed into glycolysis and the TCA cycle to produce ATP. Pths enhances the translational efficiency of a subset of mRNAs, including those encoding mitochondrial ETC components. Macrophages with elevated mitochondrial OxPhos can meet their emerging energy demands for tissue invasion.However, the absence of Atos leads to reduced levels of GR/HPR, LKR/SDH, and Porthos. This decreases the OxPhos‐generated ATP supply leading to defective tissue infiltration of the pioneering macrophages.

To investigate the metabolic changes that Atos enables, we performed untargeted comparative metabolite profiling by capillary liquid chromatography‐tandem mass spectrometry (LC‐MS/MS) of extracts from control and *atos^PBG^* embryos (Appendix Fig S3A, Dataset EV1). Most importantly, consistent with the OCR and pPDH/PDH ratio measurements, we observed a significantly decreased ATP/ADP ratio in the absence of Atos (Fig 9A and B). Matching Atos’s role in upregulating GR/HPR, in *atos^PBG^* we observed higher levels of this enzyme’s substrate, 4‐hydroxy α‐ketoglutarate (4‐Hα–KG) and of hydroxy‐L‐proline (HLP) (Fig 9C), the metabolite just upstream of 4‐Hα–KG (Fig 9A), along with significantly higher levels of dipeptides containing HLP (Fig 9D). Atos also upregulates LKR/SDH; we observed a reduction to 60% of control levels of its product α‐amino adipic semialdehyde (AASA) in *atos^PBG^*, by targeted‐metabolomics profiling (Fig 9A). We also saw less glycolytic intermediates and a backup of some metabolites whose products would normally be fed into glycolysis and the TCA cycle (Appendix Fig S3B–D). However, no indications of a metabolic shift away from mitochondrial OxPhos toward aerobic glycolysis in the absence of Atos were present (Fig 9E). We found significantly higher levels of β‐hydroxybutyrate and carnitine‐conjugated fatty acids, all of which can be broken down to acetyl CoA (Puchalska & Crawford, 2017), a main metabolite fed into the TCA cycle (Fig 9F and G). There were strong increases in thymidine, which can be catabolized to a product that is fed into glycolysis (Tabata *et al*, 2017), and uridine which can be interconverted with thymidine, along with other purine and pyrimidine nucleotides (Appendix Fig S3E and F). We observed a small increase in most amino acids in *atos^PBG^* (Appendix Fig S1G). Additionally strong reductions occurred in the glycine‐related metabolite sarcosine (N‐methylglycine) known to be a biomarker of highly metastatic prostate cancer (Appendix Fig S3H) (Sreekumar *et al*, 2009). In sum, the metabolomics in combination with our other findings strongly support the conclusion that Atos is a potent regulatory protein, increasing the efficiency and amount of OxPhos through multiple avenues to produce sufficient ATP to power tissue invasion (Fig 10).

## Discussion

We identify a key regulator of energy levels in *Drosophila* macrophages as a highly conserved and previously uncharacterized nuclear protein, Atos. Atos’ mRNA levels increase in macrophages several hours before tissue invasion and remain elevated during the process (Tomancak *et al*, 2007). Atos requires conserved domains associated with transcriptional activation and chromosomal segregation, supporting the hypothesis that it acts as a transcription factor, although it could alternatively affect other aspects of mRNA production, stabilization, or turnover. RNA sequencing indicates that Atos enables higher mRNA levels of two metabolic enzymes, increasing GR/HPR by 6.5‐fold and LKR/SDH by 25‐fold, as well as a Ddx protein, named Pths, by 10‐fold. We show that two‐fold higher levels of Pths mRNA correspond to two‐fold higher OxPhos activity, a process that generates ATP by transferring electrons produced by the TCA cycle to oxygen (Berg *et al*, 2002). Each of these three proteins is required for normal amounts of invasion, and upon forced restoration of their expression in *atos* mutant macrophages, each can restore invasion and higher pPDH/ PDH levels consistent with higher ATP. We thus favor the hypothesis, supported by our metabolomics, that these two metabolic enzymes act in pathways that ultimately feed into glycolysis or the TCA cycle and thus OxPhos. We observe active unphosphorylated PDH, consistent with low ATP, in macrophages in *atos* mutant embryo and directly detect two‐fold lower ATP/ADP levels in extracts from this strain. Given that Atos is much more highly expressed in macrophages at this stage than in the rest of the embryo, the effects in macrophages will be even greater. In sum, our data argue that the developmentally programmed upregulation of Atos triggers an increase in OxPhos by upregulating this triad of targets, ultimately significantly increasing ATP/ADP in all macrophages.

We find OxPhos not significantly required for general immune cell migration *in vivo*, just for infiltration against surrounding tissue resistance. These energy enhancing pathways appear particularly crucial in the first two invading macrophages which trade positions as they forge a path. Our *in vivo* findings align with previous *in vitro* work indicating that higher ATP levels are needed in the first cancer cell to migrate through extracellular matrix (Kelley *et al*, 2019; Zhang *et al*, 2019). However, here we also identify a concerted molecular pathway that can produce the higher energy levels required for challenging cellular tasks. At least at the RNA level Atossa is upregulated not just in the first two pioneer macrophages but in all of them. This may enable a large potential pool of macrophages to be capable of serving as the pioneers. Atossa may also support other energy intensive tasks such as apoptotic cell phagocytosis (Borregaard & Herlin, 1982), a capacity carried out by most migrating macrophages to aid development (Tepass *et al*, 1994), which also primes their inflammatory responses (Weavers *et al*, 2016).

Atos’ target Pths belongs to a family of ATP‐dependent DEAD‐box proteins that have essential roles in RNA metabolism (Bourgeois *et al*, 2016; preprint: Martin *et al*, 2021). Pths localizes to the nucleolus, where ribosomes are produced and assembled (Baßler & Hurt, 2019) and enables a higher 40S/60S ribosome ratio as well as more frequent entry of multiple ribosomes onto a single mRNA. Pths binds rRNA precursors (preprint: Martin *et al*, 2021) as does its mammalian ortholog DDX47 which is required for rRNA processing (Sekiguchi *et al*, 2006). Pths could enhance 40S subunit and mature ribosome assembly generally and still only substantially increase the TE of a specific subset of mRNAs, as we observed. mRNAs with shorter 5′UTRs, which are enriched among Pths targets, have been shown to require higher ribosome levels for efficient translation (Khajuria *et al*, 2018). Many of the mRNAs which require Pths for higher TE encode mitochondrial proteins (Morita *et al*, 2013) or others linked to mitochondrial function, such as lipid metabolism. Orthologs of Porthos’ mitochondrial targets affect many aspects of the organelle’s function, from its specialized translation, its import of proteins and their insertion into the inner membrane where the ETC resides, the formation of this inner membrane which can affect ATP production (Brandt *et al*, 2017), and its import of fatty acids as fuel. We also identify as targets accessory subunits of the ETC’s OxPhos complexes, which are involved in enhancing complex function (Graham *et al*, 1992; Phillips *et al*, 1993) as well as proteins affecting these complexes’ assembly or dimerization (Davies *et al*, 2011; Dennerlein *et al*, 2015; Formosa *et al*, 2015; Hahn *et al*, 2016). Thus, the enhanced OxPhos we detected in the presence of Porthos in our mitochondrial assay could result from improved efficiency through multiple avenues: increases in the activity, amount, localization, and assembly of OxPhos components as well as the extent of the membrane folds in which they are localized. Co‐regulation to increase translation of this set of mitochondrial proteins could thus allow a concerted enhancement of mitochondrial energy production by avoiding that individual steps become rate limiting.

Enhancing mitochondrial energy production by raising ribosome levels and thereby increasing the TE of already existing mRNAs is a complementary mechanism to those previously identified. In response to nutrient availability, mTORC1 stimulates all cap‐dependent translation and activates the transcription of ribosomal RNAs and proteins and the processing of rRNAs; this ultimately leads to higher levels of many proteins enabling growth, including those required for mitochondrial function (Borregaard & Herlin, 1982). We find that Atossa through Porthos also affects ribosomal assembly and the *atos* mutant can be rescued by TOR pathway activation. However, the unchanged p‐4EBP1 levels and the specific translational effect in the mutants argue that Atossa does not regulate TORC1 activity and that the observed rescue is due to higher general ribosome production. CLUH forms RNA granules, directly binding to, stabilizing and enhancing the translation of mRNAs encoding mitochondrial proteins involved in metabolism, while inhibiting translation of those involved in mitochondrial transcription and translation (Schatton *et al*, 2017; Pla‐Martín *et al*, 2020). We detect few shared targets between CLUH and Atossa. PGC‐1 activates OxPhos through increased transcription of mitochondrial genes themselves and thus mitochondrial biogenesis (Lin *et al*, 2005), as does its *Drosophila* ortholog, Spargel (Tiefenböck *et al*, 2010). We find Spargel in our RNAseq of invading macrophages at levels comparable to Atossa and a significant overlap in their targets. We hypothesize that these two regulators of mitochondrial function work in concert, with Atossa allowing faster and more easily reversible control of bioenergetics, and that they act beyond flies.

Atos’s vertebrate orthologs FAM214A‐B can substitute for Atos’ function and are broadly expressed along with orthologs of Atos’s three downstream effectors (Sekiguchi *et al*, 2006; Human Protein Atlas, BioGPS). We rename them AtosA and AtosB. The brain has dynamic requirements for large amounts of energy which it obtains through OxPhos (Wang *et al*, 2020). Interestingly, four different single nucleotide polymorphisms (SNPs) in FAM214A/AtosA introns were linked to more severe Alzheimer’s disease or neurofibrillary tangles in genome wide association studies while another SNP in a transcription factor‐binding region was associated with increased general intelligence (*P*‐value for all variants ≤5 × 10^−6^; FAM214A GWAS findings) (Sherva *et al*, 2020; Wang *et al*, 2020). Many neurodegenerative diseases are connected to defects in OxPhos (Koopman *et al*, 2013) or PGC‐1 (Cui *et al*, 2006; Zheng *et al*, 2010); examining if mutating AtosA in neurons results in lower levels of mitochondrial OxPhos, and decreased neural survival and signaling will be an interesting area of inquiry. AtosA is also particularly enriched within plasmacytoid dendritic cells (pDCs) and B cells (Appendix Table S2), immune cells which upregulate OxPhos during anti‐viral responses (Wu *et al*, 2016) or antibody secretion (Price *et al*, 2018) and thus could impact these immune functions. Thus, future studies should investigate the mammalian version of the regulatory network that we identified in this work and how cells might modulate it to dynamically tune energy production as demand fluctuates.

Altogether, our work uncovers a surprising molecular genetic view into the development and physiology of the organism, revealing a heretofore unsuspected cross‐regulatory mechanism spanning different levels of the biological organization of cellular metabolism, ribosome activity, and the immune system.

## Materials and Methods

### Fly work

Flies were raised on food bought from IMBA (Vienna, Austria) which was prepared according to the standard recipe of agar, cornmeal, and molasses with the addition of 1.5% Nipagin. Adults were placed in cages in a Percival DR36VL incubator maintained at 29°C and 65% humidity or a Sanyo MIR‐153 incubator at 29°C within the humidity controlled 25°C fly room; embryos were collected on standard plates prepared in house from apple juice, sugar, agar, and Nipagin supplemented with yeast from Lesaffre (Marcq, France) on the plate surface. Embryo collections for fixation (7–8 h collection) as well as live imaging (4–5 h collection) were conducted at 29°C.

### Fly lines obtained used in this work

*srpHemo‐GAL4* was provided by K. Brückner (Brückner *et al*, 2004). The RNA lines tested in this paper (Appendix Table S3) were obtained from the Bloomington *Drosophila* Stock Center (Bloomington, IN, USA) and the Vienna *Drosophila* Resource Center (VDRC, Vienna, Austria). Lines *w^‐^*; *P{w[+mC] srpHemo‐3xmCherry}*, *w^‐^*; *P{w[+mC] srpHemo‐H2A::3xmCherry}* were published previously (Gyoergy *et al*, 2018).

### Embryo fixation and immunohistochemistry

Embryos were collected on apple juice plates between 7 and 8 h at 29°C. Embryos were incubated in 50% Chlorox (DanClorix) for 5 min and washed. Embryos were fixed with 17% formaldehyde/heptane (ThermoFisher Scientific, Waltham, MA, USA) for 20 min followed by methanol or ethanol devitellinization. PDH and p‐PDH staining utilized hand‐devitellinized embryos. Fixed embryos were blocked in BBT (0.1 M PBS + 0.1% TritonX‐100 + 0.1% BSA) for 2 h at RT and then incubated overnight at 4°C. Antibodies were used at the following dilutions: Chicken anti α‐GFP (1:500) (clone 5G4, Ogris lab, MFPL, Vienna, Austria), Rat anti‐HA (Roche, Basel, Switzerland, 1:100), Mouse anti‐PDH E1α (Abcam, Cambridge, UK, ab110334, 1:200), and Rabbit antiphospho‐PDH E1α (S293) (Abcam, ab92696, 1:200). Afterward, embryos were washed in BBT for 2 h, and incubated with secondary antibodies at room temperature (RT) for 2 h, and washed again for 2 h. Secondary antibodies and Phalloidin were used at the following dilutions: anti‐rat 488 (1:300), anti‐chicken 488 (1:500), anti‐mouse 488 (1:500) or anti‐mouse 633 (1:200), anti‐rabbit 488 (1:300), and Phalloidin (1:300) (all from ThermoFisher Scientific) (Appendix Table S4). The embryos were mounted overnight at 4°C in Vectashield mounting medium (Vector Laboratories, Burlingame, USA), which contains DAPI. Embryos were placed on a slide and imaged with a Zeiss Inverted LSM800 Confocal Microscope using a Plain‐Apochromat 20×/0.8 Air Objective or a Plain‐Apochromat 63×/1.4 Oil Objective.

### S2R^+^ cell work and immunostaining

S2R^+^ cells were a gift from Frederico Mauri of the Knöblich laboratory at IMBA, Vienna) and were tested for mycoplasma contamination before utilization. Cells were grown in Schneider’s medium (Gibco) supplemented with 10% FBS (Gibco) and transfected with the *srpHemo‐HA::CG9005* (*atos*), or *UAS‐CG9005(atos)::FLAG::HA*, *UAS‐CG9253*(*pths*)*::FLAG::HA*, and *srpHemo‐GAL4* constructs using Effectene Transfection Reagent (Qiagen, Hilden, Germany) following the manufacturer’s protocol (Appendix Table S5). Transfected S2R^+^ cells were grown on Poly‐L‐Lysine coated coverslips (ThermoFisher Scientific) in complete Schneider’s medium (Gibco) supplemented with 10% FBS (Gibco) to a confluency of 60%. For antibody staining, cells were fixed with 4% paraformaldehyde (Sigma‐Aldrich) in PBS for 15 min at RT. Cells were washed three times with PBS followed by permeabilization with 0.5% Triton X‐100 (Sigma‐Aldrich) in PBS for 15 min and then blocked in BBT (see above) for at least 1 h. Antibodies were diluted in blocking buffer and incubated for 2 h at RT. Primary antibodies were used at the following working dilutions: Chicken anti‐GFP (clone 5G4, Ogris lab, MFPL, 1:100), Rat anti‐HA (Roche, 1:50), Mouse anti‐Lamin (DSHB, lamin Dm0, ADL1010, 1:50), and Mouse anti‐fibrillarin (gift from Rangan lab, 1:1). Cells were subsequently washed three times with PBS‐Triton X‐100 (0.05%) for 5 min each, followed by secondary antibody incubation in blocking/permeabilization buffer for 1 h at RT. Secondary antibodies were used at the following working dilutions: anti‐chicken 488 (1:500), anti‐rat Alexa Fluor 488 (1:50), anti‐mouse Alexa Flour 488 (1:200), and anti‐mouse Alexa Fluor 633 (1:100) (all from ThermoFisher Scientific). Cells were counterstained with DAPI (ThermoFisher Scientific) for 10 min in PBS‐Triton X‐100 (0.05%). After immunoblotting, cells were mounted with Vectashield (Sigma‐Aldrich). Images were acquired using the Zeiss inverted LSM‐800 confocal microscope. Pictures were processed with ImageJ.

### DNA isolation from single flies

Single male flies were frozen overnight before being grounded with a pellet homogenizer (VWR, Radnor, PA, USA) and plastic pestles (VWR) in 50 µl of homogenizing buffer (100 mM Tris‐HCl, 100 mM EDTA, 100 mM NaCL, and 0.5% SDS). Lysates were incubated at 65°C for 30 min. Then 5 M KAc and 6 M LiCl were added at a ratio of 1:2.5 and lysates were incubated on ice for 10 min. Lysates were centrifuged for 15 min at 20,000 *g*, supernatant was isolated and mixed with Isopropanol. Lysates were centrifuged again for 15 min at 20,000 *g*, the supernatant was discarded, and the DNA pellet was washed in 70% ethanol and subsequently dissolved in distilled water.

### FACS sorting of macrophages

For embryo collections, adult flies of either *w^+^; srpHemo‐3xmCherry* or *w^+^; CG9005^BG02278^; srpHemo‐3xmCherry* genotypes were placed into plastic cages topped with apple juice plates with yeast for egg laying. Collections were performed at 29°C at 8–20 h light‐dark cycle. Macrophages were collected from Stage 11‐early Stage 12, when macrophages initiate invasive migration into the extended germband. Briefly, adult flies laid eggs for 1 h, then the isolated plates with embryos were kept at 29°C for an additional 4 h 45 min to reach the desired age. Embryos were collected for 2 days with about 6–7 collections per day and stored meanwhile at +4°C to slow down development. Collected embryos were dissociated and the macrophages were sorted according to the procedure described before (Gyoergy *et al*, 2018). The cells were sorted using a FACS Aria III (BD) flow cytometer. Emission filters were 600LP, 610/20, and 502 LP, 510/50. Data were analyzed with FloJo software (Tree Star). The cells from the negative control embryos were sorted to set a baseline plotAbout. Approximately 1–1.5 × 10^5^ macrophages were sorted within 30 min.

### Sequencing of the macrophage transcriptome

Total RNA was isolated from the FACS‐sorted macrophages using the Qiagen RNeasy Mini kit (Hilden, Germany, Cat No. 74104). The quality and concentration of RNA was determined using the Agilent 6000 Pico kit (Santa Clara, CA USA, Cat No. 5067‐1513) on the Agilent 2100 Bioanalyzer: about 100 ng of total RNA was extracted from 1.5 × 10^5^ macrophages. RNA sequencing was performed by the CSF facility of the Vienna Biocenter according to their standard procedures (VBCF NGS website). Briefly, a cDNA library was synthesized using the QuantSeq 3′ mRNA‐seq Library Prep kit, and four replicates of each of the genotypes (*w+; +; srpHemo::3xmCherry* or *w^+^; CG9005^BG02278^*; *srpHemo‐3xmCherry)* were sequenced on the Illumina HiSeq 2500 platform.

The reads were mapped to the *Drosophila melanogaster* Ensembl BDGP6 reference genome with STAR (version 2.5.1b). The read counts for each gene were detected using HTSeq (version 0.5.4p3). The Flybase annotation (r6.19) was used in both mapping and read counting. The counts were normalized using the TMM normalization from the edgeR package in R (Dobin *et al*, 2013). Prior to statistical testing, the data were transformed and then the differential expression between the sample groups was calculated with the limma package in R. The functional analyses were done using the topGO and gage packages in R.

### Time‐lapse imaging

Embryos were dechorionated in 50% bleach for 4 min, washed with water, and mounted in halocarbon oil 27 (Sigma) between a coverslip and an oxygen permeable membrane (YSI). The anterior dorsolateral region of the embryo was imaged on an inverted multiphoton microscope (TrimScope, LaVision) equipped with a W Plan‐Apochromat 40×/1.4 oil immersion objective (Olympus). mCherry was imaged at an 820 nm excitation wavelength, using an optical parametric oscillator technology (Coherent Chameleon Compact OPO). Excitation intensity profiles were adjusted to tissue penetration depth and Z‐sectioning for imaging was set at 1 µm for tracking. For long‐term imaging, movies were acquired for 180–200 min with a frame rate of 40 s. Embryos were imaged with a temperature control unit set to 29°C.

### Image analysis

#### Macrophage cell counts

Autofluorescence of the embryo was used to measure the position of the germband to determine the stages for analysis of fixed samples. Embryos with germband retraction of between 29 and 31% were assigned to Stage 11. Embryos with 35–40% retraction (Stage 12) were analyzed for the number of macrophages that had entered the germband. Embryos with above 50–75% retraction were used for the number along the vnc and in the whole embryo. Macrophages were visualized using confocal microscopy with a Z‐resolution of 2 µm, and the number of macrophages within the germband or the segments of the vnc was calculated in individual slices (and then aggregated) using the Cell Counter plugin in Fiji. Total macrophage numbers were obtained using Imaris (Bitplane) by detecting all the macrophage nuclei as spots.

#### Macrophage tracking, speed, directionality, and time for macrophage entry analysis

Embryos in which the macrophage nuclei were labeled with *srpHemo‐H2A::3XmCherry* were imaged, and 250 × 130 × 36 µm^3^ 3D‐stacks were typically acquired with a constant 0.5 × 0.5 × 1 µm^3^ voxel size at every 40–41 s for approximately 3 h. Images acquired from multiphoton microscopy were initially processed with InSpector software (LaVision Bio Tec) to compile channels from the imaging data. Afterward, the exported files were further processed using Imaris software (Bitplane) to visualize the recorded channels in 3D and the movie from each imaged embryo was rotated and aligned along the AP axis for further tracking analysis.

To analyze the movies by Imaris, the following analyses were applied:

1. To calculate the migration parameters while macrophages migrate from the head mesoderm to the yolk zone, movies were cropped in time to that period (typically 60 min from the original movie was used for analysis).
2. To calculate the migration parameters of the macrophage moving on the yolk zone into the edge of germband, movies were acquired from the time point of the first macrophage appearing in the yolk zone and recorded until the onset of germband retraction.
3. Macrophage nuclei were extracted using the spot detection function and tracks generated in 3D over time. We could not detect all macrophages in the head mesoderm as spots because of limitations in our imaging parameters. Tracks of macrophages which migrate toward the dorsal vessel, vnc, and to the anterior of the head were omitted. The edge of the germband was detected using autofluorescence from the yolk, and the mean position of the tracks in X‐ and Y‐axes was used to restrict analysis before macrophages reach the edge of the germband.
4. Nuclei positions in XYZ‐dimensions were determined for each time point and used for further quantitative analysis.
5. The time point when the first macrophage nucleus reached the germband was defined as T0; the time point when the macrophage nucleus was within the germband and moved forward along the route between the ectoderm and mesoderm was taken as T1; T1‐T0 was defined as time for macrophage entry. T0 and T1 were determined by precisely examining macrophage position in xy and z dimensions (examination of individual 2 micron slices) over time.
6. To measure the speed along the route between the germband edge and the yolk, tracks generated from macrophages from the time when the first macrophages started to move along the mentioned path until germband retraction onset were utilized.
7. To calculate the speed of migration of the first or second macrophages in the germband, the track generated for the first or second macrophages alone was used to obtain the nuclei position in XYZ‐dimensions. Moreover, the average speed of the third through fifth macrophages moving along the same route was also measured. Speed was calculated within the first 30–35 µm of the path between the germband ectoderm and mesoderm. The mean position of the tracks in X‐ and Y‐axes was used to restrict the analysis to either of the migratory zones (head, yolk, germband entry, route along the germband ectoderm and mesoderm, route along the germband mesoderm and the yolk).

Macrophage migratory parameters, including cell speed and directionality (persistence), were calculated in Matlab (The MathWorks Inc.) from single‐cell positions in 3D for each time frame measured in Imaris (Bitplane), as described elsewhere (Smutny *et al*, 2017). Briefly, instantaneous velocities from single‐cell trajectories were averaged to obtain a mean instantaneous velocity value over the course of the measurement. To calculate directionality values, single‐cell trajectories were split into segments of equal length (*l*; *l* = 10 frames) and calculated via a sliding window as the ratio of the distance between the macrophage start‐to‐end distance (*D*) over the entire summed distance covered by the macrophage between each successive frame (*d_i_*) in a segment. Calculated directionality values were averaged over all segments in a single trajectory, and all trajectories were averaged to obtain a directionality index (*I*) for the duration of measurement (with 0 being the lowest and 1 the maximum directionality) as follows:

$$I\left(l\right)=\sum_{k=1}^{n‐1}\frac{\left(D_{k}/\sum_{i=k}^{k+1}d_{i}\right)}{n‐l}$$

where *n* defines the total number of frames, *i* the sum of frame‐to‐frame distances over one segment, and *k* the sum over all segments of a trajectory.

Embryos from the control (*w^+^; +; srpHemo::3xmCherry*) and the CG9005 mutant *(w^+^; CG9005^BG02278^; srpHemo::3xmCherry*) were used for calculating the time for macrophage entry. Briefly, 100 × 130 × 34 μm^3^ 3D‐stacks were typically acquired with a constant 0.28 × 0.28 × 2 μm^3^ voxel size at every 40–41 s for approximately 3 h.

#### Cloning of constructs

Standard molecular biology methods were used, and all constructs were sequenced by the Mycrosynth company (Vienna, Austria) before injecting into flies. The enzymes *NotI*, T4 Polynucleotide Kinase (*PNK*), and *DpnI* were obtained from New England Biolabs (Ipswich, MA, USA). PCR amplifications were performed with GoTaq G2 DNA polymerase (Promega, Madison, WI, USA) using a peqSTAR 2× PCR machine from PEQLAB (Erlangen, Germany). All Infusion cloning was conducted using an Infusion HD Cloning kit (Clontech’s European distributer). The relevant oligo sequences were chosen using the Infusion primer Tool at the Clontech website (Infusion primer design website sInit.do).

#### Construction of *srpHemo*‐CG9005

A 3,894 bp fragment containing the CG9005 ORF was amplified from the *UAS‐CG9005::FLAG::HA* construct (Appendix Table S5) (*Drosophila* Genomics Resource Centre, DGRC) using relevant primers (Appendix Table S6). The fragment was cloned into the *srpHemo* plasmid (a gift from K. Brückner (Brückner *et al*, 2004) after its linearization with *NotI*, using an Infusion HD cloning kit (Clontech’s European distributor).

#### Construction of *srpHemo‐FAM214A* and *srpHemo‐FAM214B*

Fragments of 3,225 and 1,615 bp containing the FAM214A and FAM214B ORFs, respectively, were amplified from cDNA prepared from dendritic cells (a gift from M. Sixt’s lab) with FAM214A Fwd and Rev primers, and with FAM214B Fwd and FAM214B Rev primers (Appendix Table S6). The fragments were cloned into the *srpHemo* plasmid using an Infusion HD cloning kit after its linearization with *NotI* (NEB).

#### Construction of mutant forms of *srpHemo‐atossa*

Mutant forms of Atossa (CG9005) were generated by removing the desired region from the CG9005 cDNA sequence by using inverse PCR followed by blunt end ligation and related primers (Appendix Table S6). Afterward, *atossa* mutant constructs in the Bluescript vector were amplified and cloned into the *srpHemo* plasmid after its linearization with *NotI*, using an Infusion HD cloning kit.

#### Transgenic fly line production

The *srpHemo* and *UAS* constructs (Appendix Table S5) were independently injected into syncytial blastoderm stage embryos of the M{3xP3‐RFP.attP}ZH‐86Fb (BL 24749) line (obtained from Peter Duchek of IMBA) to generate inserts on the third chromosome by C31‐mediated integration (Appendix Table S4) (Bischof *et al*, 2007; Gyoergy *et al*, 2018).

#### CRISPR sgRNA production and cloning

sgRNA target sequences for CRISPR‐Cas9 based gene knockdown of CG9253 (*pths*) were designed as 20 nt sequences upstream of an NGG PAM motif in the *Drosophila* genome (CRISPR Drosophila gRNA design website) (Bassett & Liu, 2014). The targeting oligonucleotides incorporated into *pths* sgRNAs are given in Appendix Table S6. The annealed oligo inserts were cloned into BspQ1‐digested pAC‐sgRNA‐Cas9 vector (Addgene, plasmid #49330) before transformation. Positive clones were confirmed by sequencing with the pAC‐sgRNA‐Cas9‐U6F primer (Appendix Table S6). All CRISPR‐Cas9 constructs contain three distinct cassettes for expression of Cas9, an sgRNA against *pths*, and a puromycin resistance marker.

#### Generation of *pths*‐depleted S2R^+^ *cells*

To make the stable depleted cell lines, S2R^+^ Cells (2 × 10^5^) were seeded in Schneider medium plus 10% FCS (Gibco 21720024, Sigma F9665) in a 24‐well plate. Plasmid sgRNA CRISPR *pths* was co‐transfected (1 µg of total DNA per well) with Effectene Transfection Reagent (Qiagen) following the manufacturer’s protocol. Four hours after transfection, the medium was changed and the cells were incubated for 72 h at 25°C. Cells were then transferred to a 6‐well plate before addition of 5 µg/ml Puromycin. Selection with Puromycin took place for 7 days. Surviving cells were incubated without selection medium for 24 h, after that they were added to 96‐well cell culture plates in conditioned medium at a density of 1 cell/well. After 7 days, we checked the wells for growing colonies to rule out that more than 1 colony was present per well. When cells were dense enough, we first transferred them to a 24‐, then a 12‐, and finally a 6‐well plate. Once the cells reached confluency, we extracted the genomic DNA to perform a PCR‐based prescreening of *pths*‐depleted cells to detect effective CRISPR (Appendix Table S6).

#### Quantitative Real‐Time PCR (qRT‐PCR) analysis

To verify the effective knockdown of genes, we first isolated RNA from S2R^+^ cells (1 × 10^7^ for the control and KD cells) according to the manufacturer’s protocol (Qiagen RNeasy Mini Kit Cat No./ID: 74104). We used 500 ng of isolated RNA for cDNA synthesis, according to the manufacturer’s protocol (Qiagen Omniscript RT, Cat No./ID: 205111). Afterward we performed qPCR to assess the mRNA expression of *atossa* and *pths*, using *RpS20* as an internal control. Primer sequences for *Drosophila atossa* (CG9005) and *pths* (CG9253) transcripts were designed using NCBI’s primer design tool (NCBI primer design website), and primer sequences for RpS20 gene, as an internal control gene, were obtained from the FlyPrimerBank (FlyPrimerBank website) (Appendix Table S7). We amplified 4 µl cDNA (50 ng) using 10 µl of Takyon™ No Rox SYBR MasterMix Blue dTTP (Eurogentec, Liege, Belgium), 2 µl of each reverse and forward primers (10 mM). The thermal cycling conditions were as follows: 40 cycles of amplification each consisting of 10 s at 95°C, 15 s at 60°C and 10 s at 72°C, and cooling at 4°C. The experiments were carried out in technical triplicates and with three biological replicates for each data point. The qPCR experiment was run on a LightCycler 480 (Roche, Basel, Switzerland) and data were analyzed in the LightCycler 480 Software and Prism (GraphPad Software). To calculate the fold change in *atossa* and *pths* mRNA levels compared to the house‐keeping gene's mRNA levels, we averaged the Ct values of the technical replicates of each trial. We measured Δct by subtracting the housekeeping gene Ct average from the Ct average of *atossa* or *pths*. Afterward, the 2^−Δct^ was calculated for each trial.

### Polysome profiling in *pths*‐KD S2 cells

#### RNAi treatment of S2 cells

dsRNA for *pths* (CG9253) was prepared as described by the SnapDragon manual (Snapdragon dsRNA primer design website). Briefly, template was prepared from S2 cell cDNA using the following primers designed using SnapDragon 5′‐TAATACGACTCACTATAGGATAAG GAAGGGGACAGCGAG‐3′ and the reverse primer: 5′‐TAATACGACTCACTATAGGTTTGAAATGCCAGTTCCCTC‐3′ both of which contain a T7 polymerase promoter. As a negative control, we made non‐targeting dsRNA against GFP using the following primers: 5′‐TAATACGACTCACTATAGGGGAGCGCACCATCTTCTTCAA‐3′ and 5′‐TAATACGACTCACTATAGGGCTGCTTGTCGGCCATGATATAG‐3′. We performed *in vitro* transcription overnight at 37°C using the T7 Megascript kit (AM1334) following manufacturer’s instructions (Appendix Table S6). The RNA was treated with DNAse and purified using acid‐phenol chloroform extraction and ethanol precipitated. The resulting RNA was annealed by heating at 65°C for 5 min and slow cooling to 37°C for an hour. Knocking down in S2 cells was performed using 1 µg of dsRNA as previously described (S2 RNAi knockdown reference; Zhou *et al*, 2013). 0.5–1.0 × 10^6^ cells were seeded 30 min prior to transfection to adhere. Prior to transfection, the media was changed for 500 µl of fresh media. The seeded cells were treated with 500 µl of transfection complexes per well of a 6‐well plate. Forty‐eight hours post transfection, cells were passaged to 10 cm dishes. After 3 more days, cells were harvested for further analysis.

#### Polysome profiling and polysome sequencing

Polysome sequencing was performed as described by (Flora *et al*, 2018) with minor modifications. Cells were incubated with fresh medium 2–4 h before harvesting. Cycloheximide (100 μg/ml) was first added to the medium for 3 min at RT, and the cells were subsequently centrifuged at 800 *g* for 3 min. The cell pellet was afterward washed two times with ice‐cold phosphate‐buffered saline (1× PBS, pH 7.4). The supernatant was discarded and the pellet was gently resuspended in 300 µl of lysis buffer A (300 mM NaCl, 15 mM Tris‐HCl, pH 7.5, 15 mM EDTA, 1 mg/ml heparin, 1% Triton‐X100, and 100 μg/ml cycloheximide) and lysed for 15 min on ice. The lysate was clarified by centrifugation at 8,500 *g* for 5 min at 4°C. 20% of the lysate was kept aside as an input. The clarified lysate was loaded onto a 10–50% sucrose gradient in Buffer B (300 mM NaCl, 15 mM Tris‐HCl, pH 7.5, 15 mM MgCl_2_, supplemented with 100 μg/ml cycloheximide) and centrifuged for 3 h at 35,000 rpm in an SW41 rotor in a Beckman L7 ultracentrifuge (Beckman Coulter, Krefeld, Germany). The gradients were simultaneously fractionated on a Density Gradient Fractionation System (#621140007) at 0.75 ml/min. We added 20 μl of 20% SDS, 8 μl of 0.5 M pH 8 EDTA, and 16 μl of proteinase K (#P8107S) to each polysome fraction and incubated them for 30 min at 37°C. The RNA from each fraction was extracted by standard acid phenol: chloroform purification followed by 80% ethanol precipitation. The polysome fractions were then measured for RNA content and RNAseq libraries were prepared.

#### Polysome‐seq library preparation and mRNA sequencing

The RNA was first treated with Turbo DNAse (TURBO DNA‐free Kit, Life Technologies, AM1907) and then purified using DNAse Inactivation buffer. The RNA was then centrifuged for 1.5 min at 1,000 *g* and the supernatant was collected and centrifuged once more at the same condition. The RNA quantity was determined by measuring the absorbance at 260 nm (NanoDrop 2000 spectrophotometer; Peqlab).

Poly‐A selection was performed according to manufacturer’s instructions (Bio Scientific Corp., 710 NOVA‐512991). Following Poly‐A selection, mRNA libraries were prepared according to manufacturer’s instructions (Bio Scientific Corp., NOVA‐5138‐08), except that the RNA was incubated at 95°C for 13 min to generate optimal fragment sizes. The sequencing library quantity was determined using Qubit (Thermo Fisher Scientific). The library integrity was assessed with a Bioanalyzer 2100 system (RNA 6000 Pico kit, Agilent Technologies). The libraries on biological duplicates from each genotype were subjected to 75 base‐pair single‐end sequencing on Illumina NextSeq500 at the Center for Functional Genomics (CFG).

#### Data analysis of S2 cell polysome sequencing

First the reads were assessed for their quality using FastQC. Mapping of the reads was performed against the *Drosophila* genome (dm6.01, www.fruitfly.org) using Hisat version 2.1.0. Mapped reads were then assigned to feature using feature Count version v1.6.4. To calculate Translation efficiency (TE), TPMs (transcripts per million) values for polysome libraries were calculated (Flora *et al*, 2018). All transcripts with zero reads were discarded from libraries for further analysis. The log2 ratio of TPMs between the polysome fraction and total mRNA was measured. This ratio represents TE. The TE value of each replicate was averaged, and delta TE (ΔTE) was calculated as (*pths* dsRNA TE)/(GFP dsRNA TE). Targets were defined as transcripts falling greater or less than 2 standard deviations (SD) from the median of ΔTE (Appendix Table S7).

#### Bioinformatic analysis of 5′UTRs of targets enriched in polysomes due to Porthos

5′UTRs sequences were obtained using the Bioconductor packages BSgenome. Dmelanogaster.UCSC.dm6 and TxDb. Dmelanogaster.UCSC.dm6.ensGene for each gene. 5′UTR motif enrichment: One isoform of each 5′UTR was used per gene to avoid sequence overrepresentation. Only 5′UTRs with a minimum length > 9 were considered. Meme was used to perform motif enrichment in Differential Enrichment mode using default setting. 5′UTR length: Identical 5′UTRs were filtered out of the data set to avoid overrepresentation. Average 5′UTR length was calculated per gene. 5′UTR length was plotted for each gene for Porthos polysome‐seq targets compared to nontargets. Nontargets were defined as genes expressed in S2 cells using the same cutoffs as were used in the polysome‐seq analysis, excluding Porthos polysome‐seq targets. A Welch’s *t*‐test was performed to test for significance between the length of the 5′UTRs of Porthos polysome‐seq targets compared to nontargets.

#### qPCR of polysome profile fractions

RNA was isolated individually from fractions and pooled into four categories: 40S/60S, monosome, low polysome (di‐ and trisome), high polysome (remaining fractions). The RNA pellet was washed with 80% ethanol and then air‐dried. After air‐drying, the pellet was dissolved in 40 µm of nuclease‐free water. We reverse‐transcribed and amplified 4 µl of RNA using 10 µl of Luna Universal One‐Step Reaction Mix (NEB Luna Universal One‐Step RT‐qPCR kit, Luna® Universal One‐Step RT‐qPCR Kit (New England Biolabs, Ipswich, MA, USA (Frankfurt, Germany)), 2 µl of Luna WarmStart RT Enzyme Mix, and 2 µl of each reverse and forward primers (10 mM).

The thermal cycling conditions were as follows: 40 cycles of amplification each consisting of 10 s at 95°C, 15 s at 60°C and 10 s at 72°C, and cooling at 4°C. The experiments were carried out in technical triplicates and three biological replicates for each data point. The qPCR experiment was run on a LightCycler 480 (Roche, Basel, Switzerland), and data were analyzed in the LightCycler 480 Software and Prism (GraphPad Software). To calculate the fold change in mRNA levels of target genes compared to the house‐keeping gene (GAPDH) mRNA levels, we averaged the Ct values of the technical replicates of each trial. The Ct values of different polysome fractions were normalized to the values of monosome fraction.

#### Western blots

Wild‐type and *porthos*‐*KD* S2R^+^ cells were lysed in a lysis buffer (25 mM Tris, 150 mM NaCl, 1 mM EDTA, 1% Triton X‐100) supplemented with a protease inhibitor cocktail (Complete, Roche, Basel, Switzerland) for 20 min on ice, followed by centrifugation at 14,000 *g*, 4°C for 15 min. The protein lysates were stored at −80°C. Protein concentration was determined with the Pierce BCA Protein Assay Kit (Thermo Fisher Scientific). Cell lysates (20 µg) were loaded and separated on 4–12% SDS‐PAGE gradient gels (Bio‐Rad) and blotted on Protran 0.45 nitrocellulose membranes (GE Healthcare). Membranes were blocked with 1× Pierce Clear Milk Blocking Buffer (ThermoFisher Scientific, #37587) or Blocker BLOTTO Blocking Buffer (for phosphorylated proteins, ThermoFisher Scientific, #37530) for 1 h at RT. The following primary antibodies were diluted in 1× blocking buffer and incubated overnight at 4°C: Mouse anti‐OxPhos complex V‐subunit β (mitochondrial OxPhos complex III; Invitrogen, ab92696, 1:1,000) (see also Teixeira *et al*, 2015), Rabbit anti‐MT‐ND1 (mitochondrial OxPhos complex III; Abcam, ab181848, 1:1,000), Mouse anti‐tubulin beta antibody (E7, DSHB, 1:50), mouse α‐profilin (chi 1J, DSHB, 1:50), Rabbit phospho‐4E‐BP1 (Cell Signaling Technology, 3929, 1:250). Afterward, membranes were washed 3× with TBS‐T and incubated with either Goat Anti‐Mouse IgG (H + L)‐HRP Conjugate (Bio‐Rad, #1721011) or Goat Anti‐Rabbit IgG (H + L)‐HRP Conjugate (Bio‐Rad, #1706515) secondary antibody. After 3× washing with TBS‐T, the membrane was incubated with SuperSignal West Femto Maximum Sensitivity Substrate (ThermoFisher Scientific, #34096) and the chemoluminescence signals were detected with the ChemiDoc MP Gel Imaging System (Bio‐Rad). Densitometric analysis of Western blot bands was performed with ImageJ.

#### Bioinformatic analysis of target overlap

CLUH target genes were obtained from (Schatton *et al*, 2017; Pla‐Martín *et al*, 2020) both for basal conditions and for HBSS. Genes were converted from mouse ensemble genes to orthologous fly genes using the Bioconductor package biomaRt. R was used to subset overlapping genes between porthos targets and targets from the above CLUH datasets. Significance of overlap was tested with a Fisher’s exact test.

*Drosophila* PGC‐1 (Spargel, Srl) targets were obtained from (Tiefenböck *et al*, 2010). Supplementary Table S1 indicates gene expression changes in mitochondria‐associated gene. PGC‐1a targets were defined as genes decreasing in expression in *srl^1^* mutants compared to srl^wt^, with a *P* < 0.05. However, this list was not exhaustive. Therefore, we reprocessed the underlying data from GSE14780 using the GEO2R tool build into GEO to obtain all gene expression changes between *srl^1^* mutants to *srl^wt^* using default settings. Targets were defined as genes decreasing > 2 fold with a *P*‐value < 0.05. R was used to subset overlapping genes between Porthos targets and genes from (Tiefenböck *et al*, 2010). Significance of overlap was tested with a Fisher’s exact test.

#### Extracellular flux measurements for bioenergetic profiling

Cellular respiration was assessed using a Seahorse XF96 extracellular flux analyzer (Agilent Technologies, Santa Clara, CA USA). The OCR as a measure of oxygen utilization of cells is an important indicator of mitochondrial function. The extracellular acidification rate (ECAR) is a measure of glycolytic activity measured via extracellular acidification due to lactate release, formed during the conversion of glucose to lactate during anaerobic glycolysis. Prior to measurement, wild‐type and *pths* KD cells were seeded at 10 × 10^5^ cells per well in Seahorse XF96 polystyrene tissue culture plates (Agilent) and incubated in unbuffered Seahorse RPMI assay medium (Agilent) supplemented with glucose (25 mM; Sigma‐Aldrich), sodium pyruvate (1 mM; Gibco), and glutamine (2 mM; Gibco) in a non‐CO2 incubator at 25°C and pH 7.4 for 1 h before the experiment. Cellular oxygen consumption was assessed in basal condition (prior to any addition) and after addition of oligomycin (2 μM; Agilent), Carbonyl cyanide‐4 (trifluoromethoxy) phenylhydrazone (FCCP, 2 μM; Sigma‐Aldrich), antimycin A and rotenone (both at 1 μM; Agilent). The three drugs were injected into the XF96 plate sequentially. This allowed for calculation of OCR linked to ATP production, maximal respiration capacity, and spare respiratory capacity. Basal respiration was measured prior to injection of oligomycin A. Both OCR and ECAR were measured every 4 min with a mixing of 2 min in each cycle, with 4 cycles in total for the first step and 3 cycles thereafter.

Different parameters from the OCR graph were measured as follows. ATP turnover was calculated by subtracting the “last rate measurement before oligomycin” from the “minimum rate measurement after oligomycin injection.” Maximal respiration was defined as (maximum rate measurement after FCCP) ‐ (non‐mitochondrial respiration). Spare respiratory capacity (SRC) was measured by subtracting basal respiration from maximal respiration (Mookerjee *et al*, 2017).

#### Metabolomics profiling analysis

Samples for metabolomics were assessed by the VBCF metabolomics facility according to Rao *et al* (2019) with slight modifications (https://www.viennabiocenter.org/facilities/metabolomics/). One gram of wild‐type or *atos* embryos was extracted using an ice‐cold MeOH:ACN:H2O (2:2:1, v/v) solvent mixture. A volume of 1 ml of cold solvent was added to each pellet, vortexed for 30 s, and incubated in liquid nitrogen for 1 min. The samples were thawed at room temperature and sonicated for 10 min. This cycle of cell lysis in liquid nitrogen combined with sonication was repeated three times. To precipitate proteins, the samples were incubated for 1 h at −20°C, followed by centrifugation at 13,000 rpm for 15 min at 4°C. The supernatant was removed and evaporated. The dry extracts were reconstituted in 100 μl of ACN:H2O (1:1, v/v), sonicated for 10 min, and centrifuged at 13,000 rpm for 15 min at 4°C to remove insoluble debris. The supernatants were transferred to Eppendorf tubes, shock frozen, and stored at −80°C prior to LC/MS analysis.

For metabolomics profiling, 1 μl of each sample was injected independently onto two different phase systems, on either a SeQuant ZIC‐pHILIC HPLC column (Merck, 100 × 2.1 mm; 5 µm) or on a C18‐column (Waters, ACQUITY UPLC HSS T3 150 × 2.1; 1.8 μm). Separation was performed with a flow rate of 100 µl/min, employing an Ultimate 3000 HPLC system (Thermo Fisher Scientific, Germany). In HILIC (hydrophilic interaction liquid chromatography), a 25 min gradient from 10 to 80% B was used (A: acetonitrile (ACN); B: 25 mM ammonium bicarbonate in water) and in reversed phase a gradient from 1 to 90% B in (A: 0.1% formic acid (FA) in water; B: 0.1% FA in ACN). The HPLC was coupled via electrospray ionization to a Q‐Exactive Focus (Thermo Fisher Scientific, Germany). Metabolites were ionized via electrospray ionization in polarity switching mode, acquiring high‐resolution tandem mass spectrometry data in data‐dependent acquisition mode. Combined data sets have been processed by Compound Discoverer (Thermo Fisher Scientific), searching our in‐house library and publicly available spectral libraries with a mass accuracy of 3 ppm for precursor masses and 10 ppm for fragment ion masses.

For targeted metabolomics, a volume of 1 μl of the metabolite extract was injected on a ZIC‐pHILIC HPLC column (Merck, 100 × 2.1 mm; 5 µm) operated at a flow rate of 100 μl/min, directly coupled to a TSQ Quantiva mass spectrometer (Thermo Fisher Scientific).

We used the following transitions for quantitation in the negative ion mode: AMP 346–79 *m/z*, ADP 426–134 *m/z*, ATP 506–159 *m/z*, IMP 347–79 *m/z*, GMP 362–211 *m/z*, GDP 442–344 *m/z*, GTP 522–424 *m/z*, taurine 124–80 *m/z*, malate 133–115 *m/z*, citrate 191–111 *m/z*, pyruvate 87–43 *m/z*, lactate 89–43 *m/z*, NADH 664–408 *m/z*, NAD 662–540 *m/z*, hexose phosphates 259–97 *m/z*, Acetyl CoA 808–408 *m/z*, CoA 766–408 *m/z*, succinate 117–73 *m/z*. Glutamine 147–130 *m/z*, glutamate 148–84 *m/z*, serine 106–60 *m/z* were measured in the positive ion mode.

For all transitions, the optimal collision energy was defined by analyzing pure metabolite standards. Chromatograms were manually interpreted using trace finder (Thermo Fisher Scientific), validating experimental retention times with the respective quality controls. All measurements were within the linear range of detection.

For the metabolomics analysis, the metabolite concentration was normalized using a Z‐score normalization method with the formula of y = (x − α)/λ, in which x refers to the real concentration, α indicates the mean value of all samples, and λ is the variance of all samples. The normalized concentrations of metabolites were applied to generate a heatmap, which showed the concentration difference of all metabolites. For KEGG (http://www.kegg.jp, Tokyo, Japan) pathway analysis, the clusterProfiler R package was employed.

### Statistical analysis and repeatability

We always converted the names of all the images analyzed into a code, so that they could be quantified without any knowledge of sample identity. Statistical tests as well as the number of embryos/ cells assessed are listed in the figure legends. All statistical analyses were performed using GraphPad Prism and significance was determined using a 95% confidence interval. Data points from individual experiments/ embryos were pooled to estimate mean and SEM. No statistical method was used to predetermine sample size and the experiments were not randomized. Unpaired two‐tailed *t*‐test or Mann‐Whitney was used to calculate the significance in differences between two groups and One‐way ANOVA followed by Tukey post‐test followed by Conover or Dunn’s post‐test for multiple comparisons. All measurements were performed in 3‐50 embryos. Representative images illustrated in Figs 1A–C, 2B, C and E, EV2A and B, 3B–D, 4A and I, EV4B, E and I, 5E and F, 7D, G and I, 8A, C and E, Appendix Fig S1A and B were from separate experiments that were repeated at least 3 and up to 7 times. Stills shown in Figs 1F, EV1I, 4B and EV4E are representative images from two‐photon movies, which were repeated at least 3 times. Raw data from embryo scoring and analyzed tracking output from each movie are in Source Data files for each relevant Figure.

## Author contributions

**Shamsi Emtenani:** Conceptualization; Formal analysis; Investigation; Methodology; Writing – original draft. **Elliot T Martin:** Formal analysis; Investigation; Methodology; Writing – original draft; Writing – review & editing. **Attila György:** Investigation. **Julia Bicher:** Investigation. **Jakob‐Wendelin Genger:** Investigation; Methodology; Writing – original draft; Writing – review & editing. **Thomas Koecher:** Formal analysis; Investigation; Methodology; Writing – original draft; Writing – review & editing. **Maria Akhmanova:** Investigation; Visualization; Writing – review & editing. **Mariana Guarda:** Investigation. **Marko Roblek:** Methodology. **Andreas Bergthaler:** Resources; Data curation; Funding acquisition; Writing – review & editing. **Thomas R Hurd:** Conceptualization; Resources; Methodology; Writing – review & editing. **Prashanth Rangan:** Conceptualization; Resources; Data curation; Funding acquisition; Methodology; Writing – original draft; Writing – review & editing. **Daria E Siekhaus:** Conceptualization; Resources; Data curation; Supervision; Funding acquisition; Methodology; Writing – original draft; Project administration; Writing – review & editing.

In addition to the CRediT author contributions listed above, the contributions in detail are:

Conceptualization: SE, DES, TRH, PR. Formal Analysis: SE, ETM, TK, JWG. Methodology: SE, DES, TK, ETM, JWG, MR, TRH. Investigation: SE, ETM, AG, JB, JWG, MP, TK, MA, MG, MR. Resources: TRH, AB. Manuscript writing: SE, ETM, DES with input from all authors.

## Disclosure and competing interests statement

The authors declare that they have no conflict of interest.

## Supporting information



Appendix

Expanded View Figures PDF

Movie EV1

Movie EV2

Movie EV3

Movie EV4

Dataset EV1

Source Data for Expanded View and Appendix

Source Data for Figure 1

Source Data for Figure 2

Source Data for Figure 3

Source Data for Figure 4

Source Data for Figure 5

Source Data for Figure 6

Source Data for Figure 7

Source Data for Figure 8

Source Data for Figure 9

## Data Availability

Primary reads from RNA sequencing analysis of control and *atos* mutant sorted macrophages, along with RNA sequencing analysis data of polysome profiling from control and *pths kd* S2R^+^ cells, have been deposited at NCBI’s Gene Expression Omnibus (GEO) (Edgar *et al*, 2002) as GSE167134 (https://www.ncbi.nlm.nih.gov/geo/query/acc.cgi?acc=GSE167134). All reagents are available from the Lead contact: daria.siekhaus@ist.ac.at. More processed versions of this data are in Source Data for Figs 3 and 5. Metabolomics data are in Dataset EV1.
