# Supplementary material for: Macrophage mitochondrial bioenergetics and tissue invasion are boosted by an Atossa‐Porthos axis in Drosophila
Source: EMBO J. 2022 Mar 23;41(12):e109049. doi: 10.15252/embj.2021109049 (PMC9194793; doi:10.15252/embj.2021109049)
Supplement: Supplementary file 1 — Appendix [file EMBJ-41-e109049-s005.pdf]

Macrophage mitochondrial bioenergetics and tissue invasion are promoted by an Atossa-Porthos axis in *Drosophila*

Shamsi Emtenani[1], Elliot T. Martin[2], Attila Gyoergy[1], Julia Bicher[1], Jakob-Wendelin Genger[3], Thomas Köcher[4], Maria Akhmanova[1], Mariana Pereira Guarda[1], Marko Roblek[1], Andreas Bergthaler[3], Thomas R. Hurd[5], Prashanth Rangan[2], Daria E. Siekhaus[1*]

[1] Institute of Science and Technology Austria, 3400 Klosterneuburg, Austria,

[2] University at Albany, Department of Biological Sciences, RNA Institute, Albany, NY 12222.

[3] CeMM Research Center for Molecular Medicine of the Austrian Academy of Sciences, 1090 Vienna, Austria.

[4] Vienna BioCenter Core Facilities, 1030 Vienna, Austria.

[5] Department of Molecular Genetics, University of Toronto, Toronto, Ontario, M5G 1M1, Canada.

***Corresponding author:** daria.siekhaus@ist.ac.at

Table of Contents

Appendix Figure S1. Porthos' nuclear localization helps promote macrophage germband invasion. **2**

Appendix Figure S2. Porthos increases the translation of a subset of mRNAs. **4**

Appendix Figure S3. Atos affects pyrimidine and sarcosine metabolism **6**

Appendix Table S1. Shared targets between Porthos and *Drosophila* PGC-1 (Spargel). **8**

Appendix Table S2. The enriched expression of *mFAM214A* and *mFAM214B* genes, the vertebrate orthologs of *Drosophila atos*, in vertebrate human immune cells. **9**

Appendix Table S3. Fly lines utilized in this paper. **10**

Appendix Table S4. List of key resources used in this paper. **13**

Appendix Table S5. The DNA plasmid constructs utilized in gene construction. **15**

Appendix Table S6. Oligonucleotides utilized in gene construction. **16**

Appendix Table S7. List of software tools, analytical packages, and laboratory devices utilized in this paper. **19**

Exact genotypes of *Drosophila* lines **21**

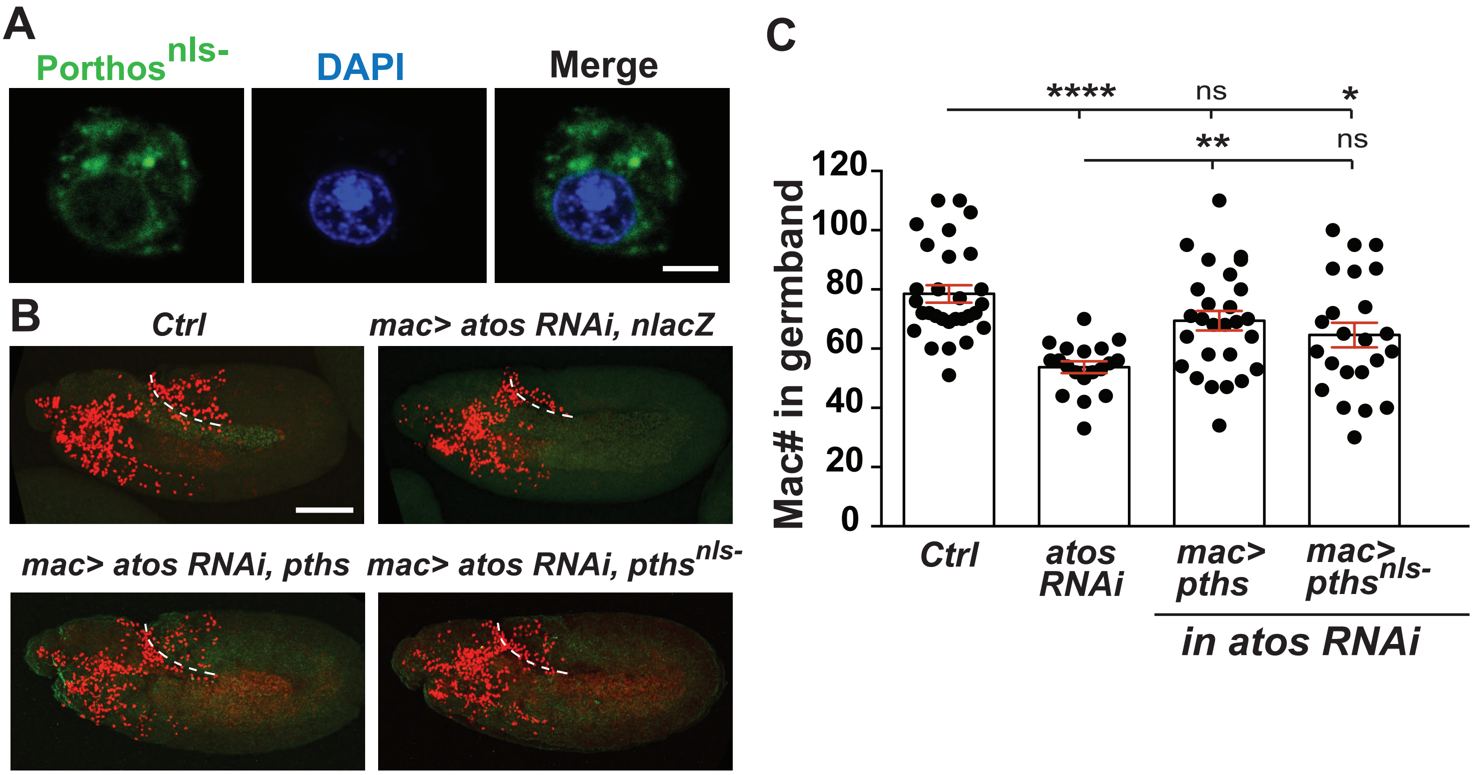
A
Porthosnls-
DAPI
Merge
B
Ctrl
mac> atos RNAi, nlacZ
mac> atos RNAi, pths
mac> atos RNAi, pthsnls-
C
****
ns
*
**
ns
Mac# in germband
120
100
80
60
40
20
0
Ctrl
atos RNAi
mac> pths
mac> pthsnls-
in atos RNAi

Apendix Figure S1. Porthos' nuclear localization helps promote macrophage germband invasion.

(A) Porthos^{nls-} (green) visualized by transfecting S2R+ cells with *UAS-pth$^{nls-::}$HA* and *srpHemo-Gal4*, and staining for the HA antibody and nuclear marker DAPI (blue). Porthos lacking the NLS is mainly in the cytoplasm.

(B-C) (B) Confocal images or (C) quantification of macrophages in the germband of early Stage 12 embryos expressing *atos RNAi* in macrophages and embryos expressing *atos RNAi* along with *pths* (*pths::FLAG::HA*) or *pths^{nls-}* (*pths^{nls-}::FLAG::HA*) in macrophages (red). For (C) control (n=29 embryos) vs. *atos RNAi* (n=19) p<0.0001; *atos RNAi* vs. its rescue with *pths* (n=28) p=0.009, or with *pths^{nls-}* (n=23) p=0.18.

Data information: Mean±SEM, ns=p>0.05, *p<0.05, **p<0.01, ****p<0.0001. One-way ANOVA with Tukey in (C). Scale bars: 3µm (A) and 30µm (B). See Source Data file 1 for Appendix Figure S1.

Appendix Fig S2

A

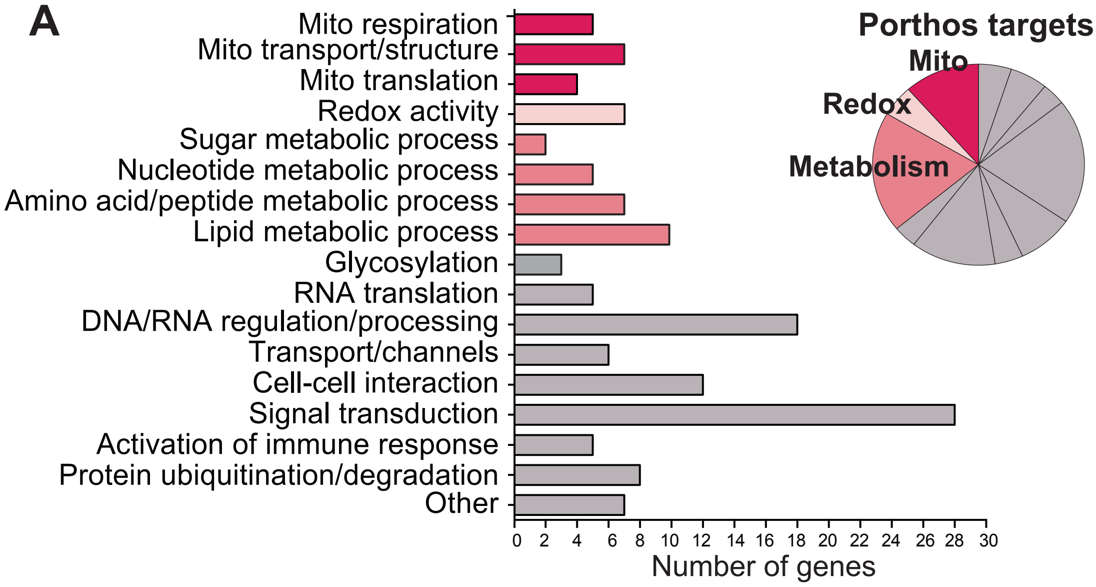

B

Biological function	Gene symbol	Description of Porthos targets	Vertebrate ortholog
DNA regulation, Transcription	CG11403	DNA DEAD/H box helicase 11	Ddx11
	CG11335	Lysyl oxidase-like 1 (Loxl1), euchromatinization	Loxl2
	CG10694	nucleotide-excision repair	Rad23a
	CG12659	Chromatin remodeling	Ino80c
	CG5441	taxi, transcription factor	Atoh1
	CG13005	Zinc finger protein 839, transcription factor	Zfp839
	CG7963	Zinc finger C2H2 transcription factor	Gm14322
	CG8021	SLIRP2, mRNA processing	Slirp
	CG8159	Regulation of transcription	Plag1
	CG11456	Regulation of transcription by RNA polymerase II	Plagl2
	CG10654	Regulation of transcription by RNA polymerase II	J23Rik
	CG31626	Regulation of transcription by RNA polymerase II	Pou2af1
	CG12442	wuc, regulation of transcription by RNA polymerase II	Lin52
	CG12320	A1 cistron-splicing factor, AAR2	Aar2
	CG12938	U7 snRNA-associated Sm-like protein LSm10	Lsm10
	CG7637	snRNA/rRNA pseudouridine synthesis	Nop10
RNA translation	CG15693	RpS20, ribosomal small protein S20	Rps20
	CG3997	RpL39, ribosomal large protein L39	Rpl39l
	CG30425	RpL41, ribosomal large protein L41	NF
	CG4061	Rtca, RNA 3'-terminal phosphate cyclase	Rtca
	CG18643	Dtd, D-aminoacyl-tRNA deacylase, tRNA metabolic process	Dtd1
Protein degradation	CG8272	SCF-dependent proteasomal ubiquitin-dependent proteolysis	Lrrc29
	CG14260	Proteasomal ubiquitin-dependent proteolysis	NF
	CG31807	Ubiquitin-protein transferase	Rfwd3
	CG8419	Ubiquitin-protein transferase	Trim45
	CG32847	Ubiquitin-protein ligase	Rnf185
	CG5001	Chaperone/unfolded protein binding	Dnajb5
	CG2046	Proteasome assembly chaperone 1	Psmg1
	CG6972	Desumoylating isopeptidase 1	Desi1
Immune cell response	CG2723	ImpE3, Ecdysone-inducible gene E3	NF
	CG1367	Cecropin A2, activity against Gram-negative bacteria	NF
	CG10794	Diptericin B, activity against Gram-negative bacteria	NF
	CG16712	IM33 peptide against systemic microbial infection	Eppin
	CG33493	Antibacterial humoral response	Ndufa5

C

Biological function	Gene symbol	Description of Porthos targets	Vertebrate ortholog
Signal transduction	CG1279	reticulon 2, ER organization and function	Rtn1
	CG5417	Srp14, protein targeting to ER	Srp14
	CG12843	Tetraspanin 42Ei, Integrin signaling	Cd63
	CG5657	Sarcoglycan β, negative regulator of EGFR pathway	Sgcb
	CG3302	Corazonin, a G-protein-coupled receptor	NF
	CG42366	Mitogen-activated protein kinase	NF
	CG8767	Mos oncogeneactivates the MAPK cascade	Mos
	CG9336	positive regulation of voltage-gated K+ channel	NF
	CG3504	inaD, fast light-induced signaling	Lnx1
	CG7916	Haemolymph juvenile hormone binding	NF
	CG18188	Damm, caspase family of cysteine proteases	Casp6
	CG9470	Metallothionein A, metal ion homeostasis	Mt1
	CG3227	insensitive, corepressor for the product of Su(H)	NF
	CG17479	Sphingosine kinase 1, regulates cell division/traffiking	NF
	CG17962	Z600, a mitotic inhibitor	NF
	CG10861	Autophagy-related 12	Atg12
	CG14937	G2/M transition of mitotic cell cycle	NF
	CG32812	negative regulation of phosphatase activity	Chp1
	CG31391	negative regulation of phosphatase activity	Ppp1r36
Transport	CG17137	Porin2, voltage-dependent anion channel 1	Vdac1
	CG7912	Sulfate transport and transmembrane transport	Slc26a11
	CG18345	Trpl, transient receptor potential-like	Trpc5
	CG32069	ER to Golgi vesicle-mediated transpor	Ier3ip1
	CG11703	Sodium:potassium-exchanging ATPase	Atp1b1
	CG5421	H(+)-transporting two-sector ATPase	Atp6ap1l
Cell-cell interaction	CG13664	Cadherin 96Cb, control of cell adhesion	Cdh6
	CG16719	Regulation of cytoskeleton organization	Spef1
	CG5987	TTLL6B, microtubule cytoskeleton organization	Ttll6
	CG4537	Cytoplasmic microtubule organization	Cript
	CG7802	Neyo, regulation of cell shape/apical constriction	NF
	CG12408	Troponin C isoform 4, control of muscle contraction	Calm4
	CG8121	Pasiflora 2 (pasi2), endothelial barrier function	NF
	CG5458	Radial spoke head protein 1, axoneme assembly	Rsph1
	CG31020	Sanpodo, cell division/cell fate determination	NF
	CG31801	Mst36Fa, spermatogenesis	NF

Appendix Figure S2. Porthos increases the translation of a subset of mRNAs.

(A) mRNAs less prevalent on polysomes (DR) in *pths-dsRNA* treated versus control *gfp-dsRNA* treated S2R+ cells. Three biological replicates for control and *pths-dsRNA*. 71% of the genes encoded proteins with predicted functions; those corresponding to a functional category are shown. **(B-C)** We list the DR mRNAs in *porthos KD* cells involved in gene regulation and RNA processing, mRNA translation, cellular transport, cell signaling, cell-cell interactions, immune responses, and protein degradation. NF: Not Found. See Source data for Figure 5.

A

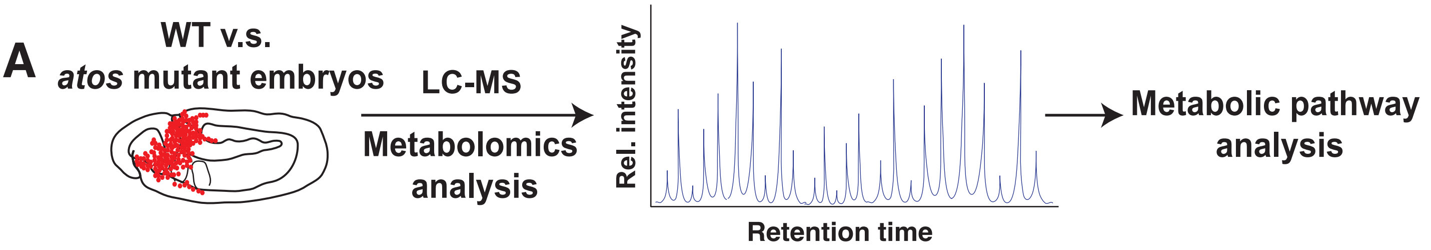

Altered metabolic pathways in *atos* embryos:

Avg fold change of analysed metabolites (*atos* v.s. WT)

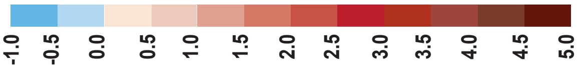

B Glycolysis

Avg FC	
0.90	Glc-6p
0.97	Fru-6p
0.52	Fru-1,6bp
0.96	DHAP
0.23	Glycerol-3p
-0.02	3-phosphoglycerate
0.67	Lactate
0.87	Pyruvate

C PPP

Avg FC	
1.61	D-Ribulose-5p
0.25	D-Xylose-5p
0.85	D-erythrose-4p
0.85	D-sedoheptulose-7p

D TCA

Avg FC	
0.33	Citrate
0.55	Isocitrate
0.55	α-ketoglutarate
0.65	Succinic semialdehyde
1.09	Succinate
1.29	Fumarate
0.46	Malate
1.57	Oxaloacetate

E Purine metabolism

Avg FC	
1.12	Xanthosine*
0.98	Methylinsoine*
1.61	Adenosine
0.96	Deoxyadenosine*
0.75	IMP
2.06	AMP
0.76	ADP
0.19	ATP
1.06	Adenylsuccinic acid
0.83	Deoxyguanosine
1.58	GMP
0.51	GDP *
0.58	GTP
1.90	Methylguanosine

F Pyrimidine metabolism

Avg FC	
3.29	Deoxyuridine***
0.71	UMP
0.27	UDP
0.03	UTP
0.16	CMP
0.94	CDP*
0.16	CTP
1.04	TMP
1.06	TDP
0.56	TTP
4.35	Thymidine****

G Amino acids

Avg FC	
-0.63	Methylglycine**
-0.04	Alanine
0.30	Serine
0.44	Proline
0.47	Valine
0.10	Threonine
0.49	Leucine
0.58	Isoleucine
0.24	Asparagine
0.40	Asparate
-0.11	Glutamine
0.38	Glutamate
0.40	Methionine
0.16	Histidine*
0.43	Phenylalanine
0.62	Arginine
0.31	Tyrosine
0.70	Tryptophan

H

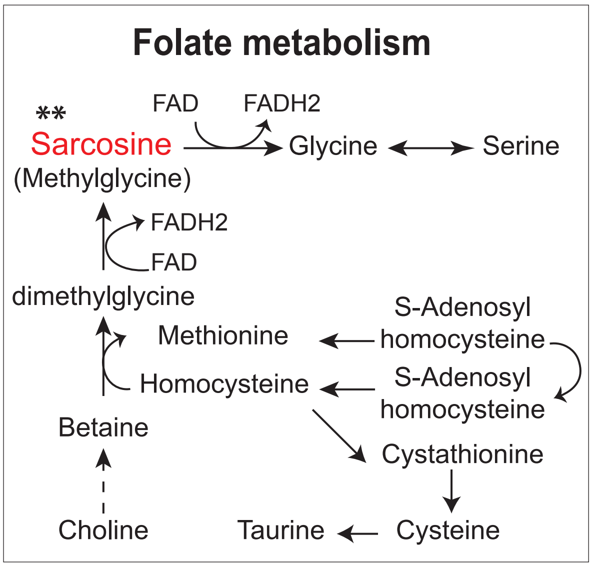

Appendix Figure S3. Atos affects pyrimidine and sarcosine metabolism

(A) The schematic illustrates the metabolic profiling procedure in wild-type and *atos*^{PBG} mutant embryos at Stage 12.

(B-G) Global non-targeted metabolite screening.

(B-D) We observe less than 1 fold increases for (B) most glycolytic intermediates along with up to 3 fold increases for metabolites from (C) the Pentose Pathway (PPP) and (D) the TCA cycle in the *atos* mutant compared to the control.

(E-F) Analysis reveals increases in some cellular nucleotide precursors and (E) purine and (F) pyridine metabolites including strong increases in thymidine, which can be catabolized to products that feed into the TCA cycle.

(G) We find a small increase in most amino acids in the *atos* mutant with a decrease in methylglycine and glutamine.

(H) Schematic shows the link between folate metabolism and glycine/serine metabolism, in which the glycine-related metabolite sarcosine (N-methylglycine) was significantly reduced in the *atos* mutant. FC: $\log_2$(Fold Change).

Data information: Metabolites with statistical significant change are shown as: *p<0.05, **p<0.01, ***p<0.001, ****p<0.0001, mean±SEM. Unpaired t test for (B-H). See Dataset EV1.

Appendix Table S1. Shared targets between Porthos and *Drosophila* PGC-1 (Spargel). Spargel targets identified from GSE14780 and Table S1 in Tiefenbock et al. 2010.

**FBID**	**Symbol**
**Shared targets btw Porthos and Spargel (GSE14780)**	
FBgn0033308	CG8736
FBgn0035343	CG16762
FBgn0036481	CG16959
FBgn0003979	Vm26Aa
FBgn0004047	Yp3
FBgn0004052	Z600
FBgn0051769	CG31769
FBgn0066084	RpL41
FBgn0085359	CG34330
FBgn0019936	RpS20
FBgn0023170	RpL39
FBgn0029606	CG14052
**Shared targets btw Porthos and Spargel (Table S1, Tiefenbock et al. 2010)**	
FBgn0010612	ATPsynG
FBgn0011227	ox

Appendix Table S2. The enriched expression of *mFAM214A* and *mFAM214B* genes, the vertebrate orthologs of *Drosophila atos*, in vertebrate human immune cells.

Gene	Tissue/cell type	Description	Expression data	Source
**FAM214A**	Plasmacytoid dendritic cells (DCs)	Low cell type specificity	RNA Seq	The Human Protein Atlas https://www.proteinatlas.org/ENSG00000047346-FAM214A/blood
	Dendritic cells (DC.DC6.123+.Bl)	Human population Avg gene expression	Population RNA Seq	Immune Cell Atlas http://immunecellatlas.net/ICA_Skyline.php?gene=FAM214A&celltype=all&organ=Blood&datatype=rnaseq&scale=Local
	Plasma B cells (B.PC)	High expression, score 6859	RNA Seq	Immgen http://rstats.immgen.org/Skyline/skyline.html
	Regulatory T cells (Cd4+, Cd25+)	score 4.57	Microarray	BioGPS http://biogps.org/#goto=genereport&id=56204
**FAM214B**	Neutrophils	Cell type enhanced (neutrophil	RNA Seq	The Human Protein Atlas https://www.proteinatlas.org/search/FAM214b
	Blood monocytes (Mo.16+.Bl, CD16+)	Human population Avg gene expression	Population RNA Seq	Immune Cell Atlas http://immunecellatlas.net/ICA_Skyline.php?gene=SLC10A2&celltype=all&organ=Blood&datatype=rnaseq&scale=Local
	Neutrophils Thio-induced pertonial neutrophils (GN.Thio.PC)	High>800 High expression, score 802	RNA Seq	Immgen http://rstats.immgen.org/Skyline/skyline.html
	Neutrophils	score 4.81	Microarray	BioGPS http://biogps.org/#goto=genereport&id=80256

Appendix Table S3. Fly lines utilized in this paper.

**Experimental models: Organisms/Strains**			
**Designation**	**Source of reference**	**Identifiers**	**Additional information**
srpHemo-Gal4	PMID: 15239955	Brückner et al. (2004)	*D. melanogaster*
srpHemo-3xmCherry	PMID: 29321168	RRID:BDSC_ 78358 and 78359	*D. melanogaster* (Gyoergy et al., 2018)
srpHemo-H2A::3xmCherry	PMID: 29321168	RRID:BDSC_ 78360 and 78361	*D. melanogaster* (Gyoergy et al., 2018)
$CG9005^{BG02278}$	Bloomington *Drosophila* Stock Center (BDSC)	RRID:BDSC_ 12768	
Df(2R)ED2222 (*Df1*)	BDSC 8911		
Df(2R)BSC259 (*Df2*)	BDSC 23159		
UAS-CG9005 RNAi 1	VDRC	VDRC: v106589	
UAS-CG9005 RNAi 2	VDRC	VDRC: v36080	
UAS-CG9005 RNAi 3	BDSC	BDSC: 33362	
srpHemo-HA::CG9005 (*srpHemo-HA::atossa*)	this paper		CG9005 amplified from genome cloned into DSPL172 (PMID: 29321168)
$srpHemo\text{-}HA::atossa^{DUF4210-}$	this paper		CG9005 amplified from genome cloned into DSPL172
$srpHemo\text{-}HA::atossa^{ChrSeg-}$	this paper		CG9005 amplified from genome cloned into DSPL172
$srpHemo\text{-}HA::atossa^{DUF4210-/ChrSeg-}$	this paper		CG9005 amplified from genome cloned into DSPL172
$srpHemo\text{-}HA::atossa^{TAD1-}$	this paper		CG9005 amplified from genome cloned into DSPL172
$srpHemo\text{-}HA::atossa^{TAD2-}$	this paper		CG9005 amplified from genome cloned into DSPL172 (PMID: 29321168)

srpHemo-HA::atossa$^{TAD1-/TAD2-}$	this paper		CG9005 amplified from genome cloned into DSPL172
srpHemo-FAM214A	this paper		FAM214A amplified from dendritic cell cDNA library cloned into *srpHemo* plasmid (DSPL172)
srpHemo-FAM214B	this paper		FAM214B amplified from dendritic cell cDNA library cloned into *srpHemo* plasmid (DSPL172)
UAS-atossa::FLAG::HA	this paper		
UAS-pth::FLAG::HA	this paper		
UAS-pth^{nls-}*::FLAG::HA*	this paper		
UAS-GR/HPR::FLAG::HA	this paper		
UAS-LKR/SDH::FLAG::HA	this paper		
UAS-HA::EGFP	this paper		
UAS-CG9253 RNAi (*pths*)	VDRC	VDRC: v36589	
UAS-CG9331 RNAi1 (*GRHPR*)	VDRC	VDRC: v44653	
UAS-CG9331 RNAi2 (GRHPR)	BDSC	BDSC: 64652	
UAS-CG9331 RNAi3 (*GRHPR*)	VDRC	VDRC: v107680	
UAS-CG7144 RNAi1 (*LKRSDH*)	VDRC	VDRC: v51346	
UAS-CG7144 RNAi2 (*LKRSDH*)	VD	VDRC: v109650	
UAS-CG2137 RNAi1 (*Gpo2*)	VDRC	VDRC: v1234	
UAS-CG2137 RNAi2 (*Gpo2*)	VDRC	BDSC: 68145	
UAS-CG11061 RNAi (*GM130*)	VDRC	BDSC: 64920	
UAS-CG11061 RNAi (*GM130*)	VDRC	VDRC: v330284	
y[-]v[-];attP40-pVALIUM22-UAS-ATPsyn Subunit C (*CG1746*) *E121Q*	VDRC	Thomas Hurd, et al., 2016.	

UAS-CG4769 RNAi1 *(Cyt-c1)*	VDRC	VDRC: v109809	
UAS-CG4169 RNAi2 *(UQCR-cp2*)	VDRC	VDRC: v100818	
UAS-CG3731 RNAi3 *(UQCR-cp1*)	VDRC	VDRC: v101350	
UAS-CG3612 RNAi (*ATP synthase F1F0*)	VDRC	VDRC: v34664	
w[*]; P{w[+mC]=UAS-da.G}52.2	BDSC	BDSC: 51669	
UAS-CG9104 RNAi (*Nprl2*)	VDRC	VDRC: *v10472*	
UAS-CG12090 RNAi *IMl1*	VDRC	VDRC: *v16390*	
UAS-CG6147 RNAi (*TSC1*)	BDSC	BDSC: *31039*	
P{GAL4-da.G32}UH1	BDSC	BDSC: 55850	

Appendix Table S4. List of key resources used in this paper.

**Antibodies and chemicals**		
**Designation**	**Source of reference**	**Identifiers**
Chicken polyclonal anti-GFP	Aves Labs	Cat# GFP-1020, RRID:AB_10000240
Rat monoclonal anti-HA	Roche	Cat# 3F10, RRID: AB_2314622
Mouse Lamin (lamin Dm0)	*Drosophila* Studies Hybridoma Bank (DSHB)	Cat# ADL1010
Mouse Fibrillarin	Rangan lab	N/A (Martin et al., 2021)
Mouse anti-Pyruvate Dehydrogenase E1-alpha subunit antibody (PDH E1α) [8D10E6]	Abcam	Cat# ab110334, RRID:AB_10866116 (Lieber et al., 2019)
Rabbit antiphoshpo-Pyruvate Dehydrogenase E1-alpha subunit (PDH E1α, S293)	Abcam	Cat# ab92696, RRID:AB_10711672 (Lieber et al., 2019)
Mouse Anti-OxPhos Complex V subunit beta	Invitrogen	Cat# A-21351, RRID:AB_221512
Anti-MT-ND1 Antibody	Abcam	Cat# ab181848, RRID:AB_2687504
Mouse Anti-NDUFS3 Monoclonal Antibody	Abcam	Cat# ab14711, RRID:AB_301429
Phospho-4E-BP1 (Thr37/46) (236B4) Rabbit mAb (Biotinylated) antibody	Cell Signaling Technology	Cat# 3929, RRID:AB_10695878
Anti α-profilin (mouse monoclonal)	Developmental Studies Hybridoma Bank (DSHB), RRID:SCR_013527	Cat# chi 1J, RRID:AB_528439
tubulin beta antibody (mouse monoclonal)	DSHB	Cat# E7 RRID:AB_528499
Goat anti-Chicken IgY (H+L) Secondary Antibody, Alexa Fluor 488	Thermo Fisher Scientific	Cat# A-11039, RRID: AB_2534096
Alexa Fluor 488 goat anti-rat	Thermo Fisher Scientific	Cat# A21212, RRID: AB_11180047
Goat anti-Mouse IgG1 Secondary Antibody, Alexa Fluor 488 conjugate	Thermo Fisher Scientific	Cat# A-21121, RRID: AB_2535764

Goat anti-Mouse IgG2b Secondary Antibody, Alexa Fluor 633 conjugate	Thermo Fisher Scientific	Cat# A-21146, RRID:AB_2535782
Goat anti-Rabbit IgG (H+L) Secondary Antibody, Alexa Fluor 488 conjugate	Thermo Fisher Scientific	Cat# R37116, RRID: AB_2556544
Phalloidin 488	Thermo Fisher Scientific	Cat# A12379, RRID:AB_2315147
Phalloidin 633	Thermo Fisher Scientific	Cat# 50-6559-05, RRID:AB_2574272
Goat-anti-rabbit IgG (H + L)-HRP conjugate	Bio-Rad	Cat# 1706515, RRID:AB_2617112
Goat-anti-mouse IgG (H + L)-HRP conjugate	Bio-Rad	Cat# 1721011 RRID:AB_11125936
Vectashield mounting medium	Vector Laboratories,RRID:SCR_000821	VectorLabs: H-1000
Vectashield Mounting medium with DAPI	Vector Laboratories,RRID:SCR_000821	VectorLabs: H-1200
Beckman Coulter 9/16x3.5 PA tubes		Cat. #331372

Appendix Table S5. The DNA plasmid constructs utilized in gene construction.

**Recombinant DNA**			
**Designation**	**Source of reference**	**Identifiers**	**Additional information**
UAS-CG9005::FLAG::HA	*Drosophila* Genomics Resource Center	DGRC: UFO03339<br>Flybase: FBgn0033638	*atossa*
UAS-CG9253::FLAG::HA	*Drosophila* Genomics Resource Center	DGRC: UFO12394<br>Flybase: FBgn0032919	*pths*
UAS-CG9331::FLAG::HA	*Drosophila* Genomics Resource Center	DGRC: UFO02643<br>Flybase: FBgn0032889	Glyoxylate reductase (NADP(+)) Hydroxypyruvate reductase (GR/HPR)
UAS-CG7144::FLAG::HA	*Drosophila* Genomics Resource Center	DGRC: UFO05689<br>Flybase: FBgn0286198	Lysine ketoglutarate reductase/saccharopine dehydrogenase (LKRSDH)
pAC-sgRNA-Cas9	Addgene	Addgene: 49330	49330 (DSPL 232)

Appendix Table S6. Oligonucleotides utilized in gene construction.

No.	Name	Sequence
1	CG9005-FP	TAGAAGCTTCTGCAAATGATACCGACAAGC<br>GTCACC
2	CG9005-RP	GTGCCTAGGCGCGCCCTAAATCCTGCCGGC<br>GCT
3	HACG9005-FP	TAGAAGCTTCTGCAAATGTACCCATACGAT<br>GTTCCAGATTACGCTGCCGCCGCCATGATA<br>CCGACAAGCGTCACC
4	HACG9005-RP	GTGCCTAGGCGCGCCAGCGTAATCTGGAAC<br>ATCGTATGGGTAGGCGGCGGCAATCCTGCC<br>GGCGCTCTC
5	infFPCG9005_NotIBluS	ACCGCGGTGGCGGCCATGTACCCATACGAT<br>GTTCCAG
6	infRPCG9005_NotIBluS	CGAAGTTATGCGGCCCTAAATCCTGCCGGC<br>GCTC
7	DUF4210^{-}CG9005-FP	TTGTGCGAGATTCGTTTGCCG
8	DUF4210^{-}CG9005-RP	AACGGACGTCCTCCAAATTGAG
9	ChrSeg^{-}CG9005-FP	AGTGCGCGACAGGAGAGC
10	ChrSeg $^{-}$CG9005-RP	AGTCGCTTCATCTGCTCGG
13	FAM214A-FP	ATGAAGCCAGACCGAGATGC
14	FAM214A-RP	TCAACATCTTGGTGAAAACTGAG
15	infFP-FAM214A	TAGAAGCTTCTGCAAATGAAGCCAGACCGA<br>GATGC
16	infRP-FAM214A	GTGCCTAGGCGCGCCTCAACATCTTGGTGA<br>AAACTG
17	FAM214B-FP	GGCTTCATGCGCCACGTG
18	FAM214B-RP	CGATCAGGGCAAAGGTGAATAACG
19	infFP-FAM214B	TAGAAGCTTCTGCAAGGCTTCATGCGCCAC<br>GTG
20	infRP-FAM214B	GTGCCTAGGCGCGCCCGATCAGGGCAAAGG<br>TGA
21	sgRNA CG9005-FP1	TTCG GCAGTCGGATGTCCGTATGCAGG
22	sgRNA CG9005-RP1	AACGCATACGGACATCCGACTGC C
23	sgRNA CG9005-FP2	TTCGCAGTTCGTAGAAGTAAGAGACGG
24	sgRNA CG9005-RP2	AACTCTCTTACTTCTACGAACTG C
25	sgRNA CG9005-FP3	TTCGCGGCGGATTCTGTCCCACCCAGG
26	sgRNA CG9005-RP3	AACGGGTGGGACAGAATCCGCCG C

27	sgRNA CG9253-FP1	TTCGGATCCAACGTGAGGCCATTCCGG
28	sgRNA CG9253-RP1	AACGAATGGCCTCACGTTGGATC C
29	sgRNA CG9253-FP2	TTCGGGCCATTCCGGTCGCCTTACAGG
30	sgRNA CG9253-RP2	AACGTAAGGCGACCGGAATGGCC C
31	sgRNA CG9253-FP3	TTCGCCCTCGTGGGGGTTAGCACGAGG
32	sgRNA CG9253-RP3	AACCGTGCTAACCCCCACGAGGG C
33	infNotI-TCHA-EGFPHA-FP	AACAGATCTGCGGCCGCATGTGTTGCCCGG GCTGCTGT
34	infNotI-TCHA-EGFP-RP	CCTCGAGCCGCGGCCGCTTAAGCGTAATCT GGCACATC
35	CG9005qPCR-FP1	TGTTCAGATTCTCGCCACCA
36	CG9005qPCR-RP1	TGAGGATTTGCCCAGCTGTT
37	CG9005qPCR-FP2	GCACGCCTTATTTGTGCGAG
38	CG9005qPCR-RP2	CCCGCATGTCGTAGGGTATC
38	CG9005qPCR-FP3	TATGCGGCAGGGAGAAAGTT
39	CG9005qPCR-RP3	GTGGTCTCTTCTGTCCACCG
40	CG9253qPCR-FP1	GCCTTACAGGGCAAGGATGT
41	CG9253qPCR-RP1	ATGCCAATCCCGCTACCAAG
42	CG9253qPCR-FP2	TCTAGGTAGCGAGGAGGAGC
43	CG9253qPCR-RP2	TGGCCTCACGTTGGATCTTC
44	CG9253qPCR-FP3	TTCGACCACGTGCTGCTATT
45	CG9253qPCR-RP3	TTGTAGCTGCGTCTGTTCGT
46	RpL32 qPCR-FP	AGCATACAGGCCCAAGATCG
47	RpL32 qPCR-RP	TGTTGTCGATACCCTTGGGC
48	RpS20qPCR-FP	ACGGTGCAAAGAACCAGAACT
49	RpS20qPCR-RP	GGAGTCTTACGGGTGGTGATG
50	pAC-sgRNA-Cas9-U6F	TTTGATTCTAAAGGAAATTTTGAAAA
51	GAPDHqPCR-FP	TAAATTCGACTCGACTCACGGT
52	GAPDHqPCR-RP	CTCCACCACATACTCGGCTC
53	CG3270qPCR-FP	TACACCAGCGCATCTACAGTG

54	CG3270qPCR-RP	ACATCTCTATGTTCTCGGCCAAA
55	CG8764qPCR-FP	GTCGGCCTTTTTCTTCGAGC
56	CG8764qPCR-RP	TGTCCTTCCAGAGTTTGCCT
57	CG6105qPCR-FP	GAACAGGCTCCTCACACAGG
58	CG6105qPCR-RP	GATGTTGCCCAGTCCTTGGC
59	CG34242qPCR-FP	CCTCACCCTATACCCCGTCA
60	CG34242qPCR-RP	ACAGGGGGTTCTACTGCCTT
61	CG1287qPCR-FP	CCAGGGAGTCGAGAGATCG
62	CGq1287qPCR-RP	GGAGTAAGCATTTTCACTGGGG
63	CG4769qPCR FP	GAGCAGATCACGGTCAAGGA
64	CG4769qPCR-RP	CGGAGGATAAGCACCGTTGT
65	CG3731qPCR-FP	GGCCACAGACGAAAAAGTCTTAAAT
66	CG3731qPCR-RP	GGTGGCAGCCGACTTGTATC
67	CG4169qPCR-FP	CTTAGTTGAACCCCGTGCGA
68	CG4169qPCR-RP	GTAGCCTCGTTTGGCGATGG
69	CG3612qPCR-FP	ATGCAGACCGGTATCAAGGC
70	CG3612qPCR-RP	GCCAGAGCGGTCTTACCAG

Appendix Table S7. List of software tools, analytical packages, and laboratory devices utilized in this paper.

Software and Algorithms		
**Designation**	**Source of reference**	**Identifiers**
ImageJ/FIJI		http://fiji.sc/ RRID:SCR_002285)
Imaris	Bitplane	http://www.bitplane. com/imaris/imaris, RRID:SCR_007370
Matlab	Mathworks	https://www.math works.com/products /matlab.html, RRID:SCR_001622
FlowJo		https://www.flowjo .com/RRID:SCR_008520
LaVision ImSpector	LaVision BioTec	http://www.lavision biotec.com/, RRID:SCR_015249
Proteome Discoverer 1.4		https://www.thermo fisher.com/order/ catalog/product/ OPTON-30795, RRID:SCR_014477
LightCycler 480 software (v. 1.5)	Roche Diagnostics	https://lifescience.roche .com/en_at/products/ lightcycler14301-480 -software-version-15.html
NLS prediction tool (NLS mapper)		NLS mapper: http://nls-mapper.iab.keio.ac.jp/
NLS prediction tool (mleg)		http://mleg.cse.sc.edu/seqNLS/
NLStradamus		http://www.moseslab.csb.utoronto.ca/NLStrad amus/
9 aaTAD Prediction Tool		https://www.med.muni.cz/9aaTAD/analysis.p hp#matches
Conserved Domain Architecture Retrieval Tool (CDART) progra m		https://www.ncbi.nlm.nih.gov/Structure/19exi ngton/19exington.cgi
Conserved Domain Database (CDD)		https://www.ncbi.nlm.nih.gov/Structure/cdd/w rpsb.cgi
Prism	GraphPad	https://www.graphpad.com/ scientific-software/prism/ RRID:SCR_002798
Flyrnai	sgRNA design	https://www.flyrnai.org/crispr/ http://tools.flycrispr.molbio.wisc.edu/targetFin

		der/
NCBI primer design tool	Primer design	https://www.ncbi.nlm.nih.gov/tools/primer-blast/
Infusion primer tool	Clontech website	http://bioinfo.clontech.com/infusion/convertPcrPrimersInit.do
HISAT2		https://ccb.jhu.edu/software/hisat2/index.shtml
MEME Suite		http://meme-suite.org/doc/overview.html
Homer (v4.10.4)		http://homer.ucsd.edu/homer/

**Others**		
**Designation**	**Source of reference**	**Identifiers**
Nikon Eclipse Ti Inverted widefield Microscope	Nikon	https://www.nikoninstruments.com/en_EU/Products/Inverted-Microscopes/Eclipse-Ti-E
Zeiss LSM 800 Confocal Microscope	Zeiss	https://www.zeiss.com/microscopy/us/products/confocal-microscopes.html
LaVision 2-Photon Inverted Microscope	LaVision BioTec	http://www.lavisionbiotec.com/products/trim-scope-ii-1.html
YSI Stretch membranes	YSI	https://www.ysi.com/Accessory/id-066155/Membranes-10-Pack-Standard
LightCycler 480	Roche Diagnostics	Idaho Technology Inc., Salt Lake City, UT, USA.
FACS Aria III (BD) flow cytometer		
Leica SP8 FALCON inverted confocal	WLL, FALCON, Leica	https://www.leica-microsystems.com/products/confocal-microscopes/p/dive/
Beckman L7 ultracentrifuge	Beckman Coulter, Krefeld, Germany	

Exact genotypes of *Drosophila* lines

Here, we list the lines used in each figure.

Figures 1 and EV1

Fig 1A-C: Control: *w-; +; srpHemo-H2A::3xmCherry*. CG9005 mutant*: w-; P{EP}CG9005*^{BG02278}*; srpHemo-H2A::3xmCherry*. CG9005 rescue*: w-; P{EP}CG9005*^{BG02278}*; srpHemo-CG9005, srpHemo-H2A::3xmCherry*. **Fig 1D:** Control: *w-; +; srpHemo-H2A::3xmCherry*. CG9005 mutant: *w-; P{EP}CG9005*^{BG02278}*; srpHemo-H2A::3xmCherry*, Df1 cross: *w-; P{EP}CG9005*^{BG02278}*/ Df(2R)ED2222; srpHemo-H2A::3xmCherry*. Df2 cross: *w-; P{EP}CG9005*^{BG02278}*/Df(2R)BSC259; srpHemo-H2A::3xmCherry*. CG9005 rescue: *w-; P{EP}CG9005*^{BG02278}*; srpHemo-CG9005, srpHemo-H2A::3xmCherry*. **Fig 1E:** Control 1: *w*$^-$ *P(w+)UAS-dicer/w-; P{attP,y[+],w[3`]/+; srpHemo-Gal4 UAS-GFP*. CG9005 RNAi 1: *UAS-Dicer2/w-; CG9005 RNAi* (*v106589*)*/+; srpHemo-Gal4 UAS-GFP, UAS-H2A::RFP/+*. Control 2: *w*$^-$ *P(w+)UAS-dicer/w-; +; srpHemo-Gal4 UAS-GFP***.** CG9005 RNAi 2: *UAS-Dicer2/ w-; CG9005 RNAi* (*v36080*)*/+; srpHemo-Gal4 UAS-GFP, UAS-H2A::RFP/.* Control 3: *w*$^-$ *P(w+)UAS-dicer/w-; P{attP,y[+],w[3`]/+; srpHemo-Gal4 UAS-GFP*. CG9005 RNAi 3: *UAS-Dicer2/w-; CG9005 RNAi* (*v33362*)*/+; srpHemo-Gal4 UAS-GFP, UAS-H2A::RFP/+*. **Fig 1F-L:** Control: *w-; +; srpHemo-H2A::3xmCherry.* CG9005 mutant: *w-; P{EP}CG9005*^{BG02278}*; srpHemo-H2A::3xmCherry.*

Fig EV1A,H: Control 1: *w*$^-$ *P(w+)UAS-dicer/w-; P{attP,y[+],w[3`]/+; srpHemo-Gal4 UAS-GFP***,** CG9005 RNAi 1: *UAS-Dicer2/w-; CG9005 RNAi* (*v106589*)*/+; srpHemo-Gal4 UAS-GFP, UAS-H2A::RFP/+*. Control 2: *w*$^-$ *P(w+)UAS-dicer/w-; +; srpHemo-Gal4 UAS-GFP*. CG9005 RNAi 2*: UAS-Dicer2/w-; CG9005 RNAi* (*v36080*)*/+; srpHemo-Gal4 UAS-GFP, UAS-H2A::RFP/+*. Control 3*: w*$^-$ *P(w+)UAS-dicer/w-; P{attP,y[+],w[3`]/+; srpHemo-Gal4 UAS-GFP*. CG9005 RNAi 3: *UAS-Dicer2/ w-; CG9005 RNAi* (*v33362*)*/+; srpHemo-Gal4 UAS-GFP, UAS-H2A::RFP/+*. **Fig EV1B:** Control: *w-; +; srpHemo-H2A::3xmCherry.* mutant: *w-; P{EP}CG9005*^{BG02278}*; srpHemo-H2A::3xmCherry*. Df1 cross: *w-; P{EP}CG9005*^{BG02278}*/Df(2R)ED2222; srpHemo-H2A::3xmCherry*. Df2 cross: *w-; P{EP}CG9005*^{BG02278}*/Df(2R)BSC259; srpHemo H2A::3xmCherry*. CG9005 rescue: *w-; P{EP}CG9005*^{BG02278}*; srpHemo-CG9005, srpHemo-H2A::3xmCherry*. **Fig EV1C,G:** Control: *w-; +; srpHemo- H2A::3xmCherry.* mutant: *w-; P{EP}CG9005*^{BG02278}*; srpHemo-H2A::3xmCherry.* **Fig EV1D:** Control 1: *w*$^-$ *P(w+)UAS-dicer/w-; P{attP,y[+],w[3`]/+; srpHemo-Gal4 UAS-GFP***.** CG9005 RNAi 1: *UAS-Dicer2/w-; CG9005 RNAi* (*v106589*)*/+; srpHemo-Gal4 UAS-GFP, UAS-H2A::RFP/+.* **Fig EV1E:** Control 2: *w*$^-$ *P(w+)UAS-dicer/w-; +; srpHemo-Gal4 UAS-GFP*. CG9005 RNAi 2*: UAS-Dicer2/w-; CG9005 RNAi* (*v36080*)*/+; srpHemo-Gal4 UAS-GFP, UAS-H2A::RFP/+*. **Fig EV1F:** Conrol 3*: w*$^-$ *P(w+)UAS-dicer/w-; P{attP,y[+],w[3`]/+; srpHemo-Gal4 UAS-GFP*. CG9005 RNAi 3*: UAS-Dicer2/w-; CG9005 RNAi* (*v33362*)*/+; srpHemo-Gal4 UAS-GFP, UAS-H2A::RFP/+*. **Fig EV1I-L:** Control: *w-; +; srpHemo-H2A::3xmCherry*. CG9005 mutant: *w-; P{EP}CG9005*^{BG02278}*; srpHemo-H2A::3xmCherry.*

Figures 2 and EV2

Fig 2B: *w-;+; UAS-atossa::FLAG::HA, srpHemo-Gal4, srpHemo-H2A::3xmCherry*. **Figs 2C,D:** Control: *w-; +; srpHemo- H2A::3xmCherry, atos* mutant: *w-; atossa*^{BG02278}*; srpHemo-H2A::3xmCherry,* Atossa rescue: *w-; atossa*^{BG02278}*; srpHemo- atossa, srpHemo-H2A::3xmCherry.* Rescue: *w-; atossa*^{BG02278}*; srpHemo- atossa*$^{DUF4210-}$*, srpHemo-H2A::3xmCherry*. Rescue: *w-; atossa*^{BG02278}*; srpHemo- atossa*$^{CherSeg-}$*, srpHemo-H2A::3xmCherry*, rescue: *w-; atossa*^{BG02278}*; srpHemo- atossa*$^{DU4210-/CherSeg-}$*, srpHemo-H2A::3xmCherry*. Rescue: *w-; atossa*^{BG02278}*; srpHemo- atossa*$^{TAD1-/TAD2-}$*, srpHemo-H2A::3xmCherry***. Fig 2E,F:** Control: *w-; +; srpHemo-H2A::3xmCherry. aost* mutant: *w-; atossa*^{BG02278}*; srpHemo-H2A::3xmCherry.* Rescue: *w-; atossa*^{BG02278}*; srpHemo-FAM214A, srpHemo-H2A::3xmCherry.* Rescue: *w-; atossa*^{BG02278}*; srpHemo-FAM214B, srpHemo-H2A::3xmCherry.* **Fig EV2B:** Rescue: *w-; atossa*^{BG02278}*; srpHemo-atossa*$^{TAD1-}$*, srpHemo-H2A::3xmCherry*. Rescue: *w-; atossa*^{BG02278}*; srpHemo-atossa*$^{TAD2-}$*, srpHemo-H2A::3xmCherry*. **Fig EV2C:** Control: *w-; +; srpHemo-H2A::3xmCherry, atos* mutant: *w-; atossa*^{BG02278}*; srpHemo-H2A::3xmCherry.* Atossa rescue: *w-; atossa*^{BG02278}*; srpHemo-atossa, srpHemo-H2A::3xmCherry.* Rescue: *w-; atossa*^{BG02278}*; srpHemo-atossa*$^{TAD1-}$*, srpHemo-H2A::3xmCherry.* Rescue: *w-; atossa*^{BG02278}*; srpHemo-atossa*$^{TAD2-}$*, srpHemo-H2A::3xmCherry*. **Fig EV2D:** Control: *w-; +; srpHemo-H2A::3xmCherry.* mutant: *w-; atossa*^{BG02278}*; srpHemo-H2A::3xmCherry.* Rescue: *w-; atossa*^{BG02278}*; srpHemo-atossa, srpHemo-H2A::3xmCherry.* Rescue: *w-; atossa*^{BG02278}*; srpHemo-atossa*$^{DUF4210-}$*, srpHemo-H2A::3xmCherry.* Rescue: *w-; atoss*^{BG02278}*; srpHemo-atossa*$^{CherSeg-}$*, srpHemo-H2A::3xmCherry.* Rescue: *w-; atossa*^{BG02278}*; srpHemo-atossa*$^{DUF4210-/CherSeg-}$*, 2srpHemo-H2A::3xmCherry.* Rescue: *w-; atossa*^{BG02278}*; srpHemo-atossa*$^{TAD1-}$*, srpHemo-H2A::3xmCherry.* Rescue: *w-; atossa*^{BG02278}*; srpHemo-atossa*$^{TAD2-}$*, srpHemo-H2A::3xmCherry.* Rescue: *w-; atossa*^{BG02278}*; srpHemo-atossa*$^{TAD1-/TAD2-}$*, srpHemo-H2A::3xmCherry*. **Fig EV2E:** Control: *w-; +; srpHemo-H2A::3xmCherry, atos* mutant: *w-; atossa*^{BG02278}*; srpHemo-H2A::3xmCherry.* Atossa rescue: *w-; atossa*^{BG02278}*; srpHemo-atossa, srpHemo-H2A::3xmCherry.* Rescue: *w-; atossa*^{BG02278}*; srpHemo-FAM214A, srpHemo-H2A::3xmCherry.* Rescue: *w-; atossa*^{BG02278}*; srpHemo-FAM214B, srpHemo-H2A::3xmCherry.*

Figures 3 and EV3

Fig 3B,F: Control (for *pths* or *CG9253*): *w/y,w[1118]; P{attP,y[+],w[3']}; srpHemo-Gal4, srpHemo-H2A::3xmCherry/+*. CG9253 RNAi (*pths*): *w-; pths RNAi* (*v36589*)*/+; srpHemo-Gal4, srpHemo-H2A::3xmCherry/+*. **Fig 3C:** Control 1 (for CG9331 or *GR/HPR*): *w/y,w[1118]; P{attP,y[+],w[3']}; srpHemo-Gal4, srpHemo-H2A::3xmCherry/+*. CG9331 RNAi 1 (GR/HPR): *UAS-Dicer2/ w-; GR/HPR RNAi* (*v44653*)*/+; srpHemo-Gal4, srpHemo-H2A::3xmCherry/+*. **Fig 3D:** Control 1 (for CG7144 or *LKR/SDH*): *w/y,w[1118]; P{attP,y[+],w[3']}; srpHemo-Gal4, srpHemo-H2A::3xmCherry/+*. CG7144 RNAi 1 (*LKR/SDH*): *UAS-Dicer2/ w-; LKR/SDH RNAi* (*v51346*)*/+; srpHemo-Gal4, srpHemo-H2A::3xmCherry/+.* **Fig 3F:** Control 1: *w/y,w[1118]; P{attP,y[+],w[3']}; srpHemo-Gal4, srpHemo-H2A::3xmCherry/+*. CG9331 RNAi 1 *(GR/HPR*): *UAS-Dicer2/ w-; GR/HPR RNAi* (*v44653*)*/+; srpHemo-Gal4, srpHemo-H2A::3xmCherry/+. Control* 2: *w/y,w[1118]; P{attP,y[+],w[3']}; srpHemo-Gal4, srpHemo-*

H2A::3xm- Cherry/+, CG9331 RNAi 2 (*GR/HPR*): *UAS-Dicer2/ w-; GR/HPR RNAi* (*v10780*)*/+; srpHemo-Gal4, srpHemo-H2A::3xmCherry/+*. Control 3: *w/y,w[1118]; P{attP,y[+],w[3'*]*}; srpHemo-Gal4, srpHemo-H2A::3xmCherry/+*. CG9331 RNAi 3 *(GR/HPR): UAS-Dicer2/ w-; GR/HPR RNAi* (*64652*)*/+; srpHemo-Gal4, srpHemo-H2A::3xmCherry/+*. **Fig 3G:** Control 1: *w/y,w[1118]; P{attP,y[+],w[3'*]*}; srpHemo-Gal4, srpHemo-H2A::3xmCherry/+*. CG7144 RNAi 1 (*LKR/SDH*): *UAS-Dicer2/ w-; LKR/SDH RNAi (v51346)/+; srpHemo-Gal4, srpHemo-H2A::3xmCherry/+*. Control 2: *w/y,w[1118]; P{attP,y[+],w[3'*]*}; srpHemo-Gal4, srpHemo-H2A::3xmCherry/+*, CG7144 RNAi 2 *(LKR/SDH): UAS-Dicer2/ w-; LKR/SDH RNAi* (*v109650*)*/+; srpHemo-Gal4, srpHemo-H2A::3xmCherry/+*. **Fig EV3A-B:** Control: *w-; +; srpHemo-H2A::3xmCherry*, mutant*: w-; P{EP}CG9005*^{BG02278}*; srpHemo-H2A::3xmCherry.* **Fig EV3D:** Control (for CG9253 or *pths*): *w/y,w[1118]; P{attP,y[+],w[3'*]*}; srpHemo-Gal4, srpHemo-H2A::3xmCherry/.* CG9253 RNAi (*pths*): *w-; pths RNAi* (*v36589)/+; srpHemo-Gal4, srpHemo-H2A::3xmCherry/+*. **Fig EV3E:** Control 1: *w/y,w[1118]; P{attP,y[+],w[3'*]*}; srpHemo-Gal4, srpHemo-H2A::3xmCherry/+*. CG7144 RNAi 1 (*LKR/SDH*): *UAS-Dicer2/ w-; LKR/SDH RNAi* (*v51346*)*/+; srpHemo-Gal4, srpHemo-H2A::3xmCherry/+*, Control 2: *w/y,w[1118]; P{attP,y[+],w[3'*]*}; srpHemo-Gal4, srpHemo-H2A::3xmCherry/+*. CG7144 RNAi 2 (*LKR/SDH*): *UAS-Dicer2/w-; LKR/SDH RNAi* (*v109650*)*/+; srpHemo-Gal4, srpHemo-H2A::3xmCherry/+*. **Fig EV3F:** Control 1: *w/y,w[1118]; P{attP,y[+],w[3'*]*}; srpHemo-Gal4, srpHemo-H2A::3xmCherry/+. CG9331* RNAi 1 (*GR/HPR*)*: UAS-Dicer2/ w-; GR/HPR RNAi* (*v44653*)*/+; srpHemo-Gal4, srpHemo-H2A::3xmCherry/+*, Control 2: *w/y,w[1118]; P{attP,y[+],w[3'*]*}; srpHemo-Gal4, srpHemo-H2A::3xmCherry/+*. CG9331 RNAi 2 (*GR/HPR*)*: UAS-Dicer2/ w-; GR/HPR RNAi* (*v10780*)*/+; srpHemo-Gal4, srpHemo-H2A::3xmCherry/+*. Control 3: *w/y,w[1118]; P{attP,y[+],w[3'*]*}; srpHemo-Gal4, srpHemo-H2A::3xmCherry/.* CG9331 RNAi 3 (*GR/HPR*): *UAS-Dicer2/ w-; GR/HPR RNAi (64652)/+; srpHemo-Gal4, srpHemo-H2A::3xmCherry/+*. **Fig EV3G:** Control 1: *w/y,w[1118]; P{attP,y[+],w[3'*]*}; srpHemo-Gal4, srpHemo-H2A::3xmCherry/+*. CG2137 RNAi 1 (*Gpo2*): *w-/y,w[1118]; Gpo2 RNAi* (*v41234*)*/+; srpHemo-Gal4, srpHemo-H2A::3xmCherry/+*. Control 2: *w/y,w[1118]; P{attP,y[+],w[3'*]*}; srpHemo-Gal4, srpHemo-H2A::3xmCherry/+*. CG2137 RNAi 2 (*Gpo2*): *w-/y,w[1118]; Gpo2 RNAi* (*68145*)*/+; srpHemo-Gal4, srpHemo-H2A::3xmCherry/+*. **Fig EV3H:** Control 1: *w/y,w[1118]; P{attP,y[+],w[3'*]*}; srpHemo-Gal4, srpHemo-H2A::3xmCherry/+*. CG11061 RNAi 1 (*GM130*): *w-/y,w[1118]; GM130 RNAi* (*v330284*)*/+; srpHemo-Gal4 UAS-GFP, UAS-H2A::RFP/+*. Control 2: *w/y,w[1118]; P{attP,y[+],w[3'*]*}; srpHemo-Gal4, srpHemo-H2A::3xmCherry/+*. CG11061 RNAi 2 (*GM130*): *w-/y,w[1118]; GM130 RNAi* (*64920*)*/+; srpHemo-Gal4, srpHemo-H2A::3xmCherry/+*.

Figures 4 and EV4

Fig 4A: *w-;+; UAS-pths::FLAG::HA, srpHemo-Gal4, srpHemo::3xmCherry.* **Fig 4B-H:** Control: *w/y,w[1118]; P{attP,y[+],w[3'*]*}; srpHemo-Gal4, srpHemo-H2A::3xmCherry/+*. CG9253 RNAi (*pths*): *w-; pths RNAi*

(*v36589*)*/+; srpHemo-Gal4, srpHemo-H2A::3xmCherry/+*. **Fig 4I-J:** Control: *w-; +; srpHemo-H2A::3xmCherry*. *atos* mutant: *w-; atossa*^{BG02278}*; srpHemo-H2A::3xmCherry*, Atos rescue: *w-; atossa*^{BG02278}*; UAS-atos::FLAG::HA, srpHemo-Gal4, srpHemo-H2A::3xmCherry*. Rescue: *w-; atossa*^{BG02278}*; UAS-pths::FLAG::HA, srpHemo-Gal4, srpHemo-H2A::3xmCherry.*
Fig EV4C-H: Control: *w/y,w[1118]; P{attP,y[+],w[3']}/+; srpHemo-Gal4, srpHemo-H2A::3xmCherry/+*. CG9253 RNAi (*pths*): *w-; pths RNAi* (*v36589*)*/+; srpHemo-Gal4, srpHemo-H2A::3xmCherry/+*. **Fig EV4I:** *w-; atossa*^{BG02278}*; UAS-pths::FLAG::HA, srpHemo-Gal4, srpHemo-3xmCherry.*

Figure 5
Fig 5E,G: Control: *w-; UAS-nlacz/+; srpHemo-Gal4, srpHemo H2A::3xmCherry/+*. *atos* RNAi: *w-; UAS-nlacz/+; CG9005 RNAi* (*v36080*)*/srpHemo-Gal4, srpHemo-H2A::3xmCherry*. Rescue*: w-; +; srpHemo-Gal4, srpHemo-H2A::3xmCherry; CG9005 RNAi* (*v36080*)*/ Nprl2 RNAi* (*v10472*). Rescue: *w-; +; srpHemo-Gal4, srpHemo-H2A::3xmCherry; CG9005 RNAi* (*v36080*)*/ IMl1 RNAi* (*v16390*). Rescue: *w-; +; srpHemo-Gal4, srpHemo-H2A::3xmCherry; CG9005 RNAi* (*v36080*)*/ TSC1 RNAi (31039).*
Fig 5F,H: Control: *w-; UAS-nlacz/+; srpHemo-Gal4, srpHemo-H2A::3xmCherry/+*. *pths* RNAi: *w-; UAS-nlacz/+; pths RNAi* (*v36589*)*/ srpHemo-Gal4, srpHemo-H2A::3xmCherry*. Rescue: *w-; srpHemo-Gal4, srpHemo-H2A::3xmCherry/+; pths RNAi* (*v36589*)*/ Nprl2 RNAi* (*v10472*). Rescue*: w-; srpHemo-Gal4, srpHemo-H2A::3xmCherry/+; pths RNAi* (*v36589*)*/ IMl1 RNAi* (*v16390*). Rescue: *w-; srpHemo-Gal4, srpHemo-H2A::3xmCherry/+; pths RNAi* (*v36589*)*/ TSC1 RNAi (31039).*

Figures 7 and EV5
Fig 7D-F and Fig EV5G: Control: *w-; +; srpHemo-Gal4, srpHemo-H2A::3xmCherry,* dominant negative inhibitor of Complex V (CV-DN): *w-;UAS-CVDN; srpHemo-Gal4, srpHemo-H2A::3xmCherry.* **Fig 7G-H and Fig EV5F,H:** Control*: w-; P{attP,y[+],w[3`]}/+; srpHemo-Gal4, srpHemo-H2A::3xmCherry.* Complex III (Cyt-c1, CG4769) RNAi 1: *w-; cyt-c1 RNAi* (*v109809*)*/+; srpHemo-Gal4, srpHemo-H2A::3xmCherry.* Complex III (UQCR-cp1, CG3731) RNAi 2: *w-; UQCR-cp1 RNAi (v101350)/+; srpHemo-Gal4, srpHemo-H2A::3xmCherry.* Complex III (UQCR-cp2, CG4169) RNAi 3: *w-; UQCR-cp2 RNAi* (*v100818*)*/+; srpHemo-Gal4, srpHemo - H2A::3xmCherry.* Complex V (ATP synthase F1F0, CG3612) RNAi: *w-; RNAi* (*v34664*)*/+; srpHemo-Gal4, srpHemo-H2A::3xmCherry.* **Fig 7I,J:** Control: *w-;* +; *srpHemo-Gal4, srpHemo-3xmCherry. atos* mutant: *w-; atossa*^{BG02278}*; srpHemo-Gal4, srpHemo-3xmCherry.* Control: *w/y,w[1118]; P{attP,y[+],w[3']};srpHemo-Gal4, srpHemo-3xmCherry/+*, CG9253 RNAi (*pths*): *w-; pths RNAi* (*v36589*)*/+; srpHemo-Gal4, srpHemo-3xmCherry/+,* Control: *w-; +; srpHemo-Gal4, srpHemo-3xmCherry. CV-DN*: *w-;UAS-CV DN; srpHemo-Gal4, srpHemo-3xmCherry.* **Fig EV5E:** Control*: w-; P{attP,y[+],w[3`]}/+; da.G32-Gal4/+*. Complex III (Cyt-c1, CG4769) RNAi 1: *w-; cyt-c1 RNAi* (*v109809*)*/+; da.G32-Gal4/+*. Complex III (UQCR-cp1, CG3731) RNAi 2: *w-; UQCR-cp1 RNAi (v101350)/+; da.G32-Gal4/+.* Complex III (UQCR-cp2, CG4169) RNAi 3: *w-; UQCR-cp2 RNAi*

(*v100818*)/+; *da.G32-Gal4*/+. Complex V (ATP synthase F1F0, CG3612) RNAi: *w-; RNAi* (*v34664*)/+; *da.G32-Gal4/+.*

Figure 8

Fig 8A-B: Control: *w-;* +; *srpHemo-Gal4, srpHemo-3xmCherry. atos* mutant: *w-; atossaBG02278; srpHemo-Gal4, srpHemo-3xmCherry.* Rescue: *w-; atossa*^{BG02278}*; UAS-pths::FLAG::HA, srpHemo-Gal4, srpHemo-3xmCherry.* Rescue: *w-; atossa*^{BG02278}*; UAS-GR/HPR::FLAG::HA, srpHemo-Gal4, srpHemo-3xmCherry*. Rescue: *w-; atossa*^{BG02278}*; UAS-LKR/SDH::FLAG::HA*, *srpHemo-Gal4, srpHemo-3xmCherry.* **Fig 8C-D:** Control: *w-;* +; *srpHemo-Gal4, srpHemo-3xmCherry. atos* utant: *w-; atossa*^{BG0227}*; srpHemo-Gal4, srpHemo-H2A::3xmCherry.* Rescue: *w-; atossa*^{BG02278}*; UAS-atos::FLAG::HA, srpHemo-Gal4, srpHemo-H2A::3xmCherry.* Rescue: *w-; atossa*^{BG02278}*; UAS-GR/HPR*::*FLAG::HA, srpHemo-Gal4, srpHemo-H2A -3xmCherry.* Rescue: *w-; atossa*^{BG02278}*; UAS-LKR/SDH*$^{::}$*FLAG::HA*, *srpHemo-Gal4, srpHemo-H2A::3xmCherry.* **Fig 8E-F:** Control: *w-;* +; *srpHemo-Gal4, srpHemo-3xmCherry. atos* mutant: *w-; atossa*^{BG0227}*; srpHemo-3xmCherry.* Rescue: *w-; atossa*^{BG0227}*; srpHemo-mFAM214A, srpHemo::3xmCherry.* Rescue: *w-; atossa*^{BG0227}*; srpHemo-mFAM214B, srpHemo::3xmCherry.*

Figure 9

Fig 9B-G: Control: *w-; +; srpHemo-3xmCherry*, mutant: *w-; atossa*^{BG02278}*; srpHemo-3xmCherry.*

Appendix Figure S1

Appendix Fig S1B-C: Control *w-; srpHemo-Gal4, srpHemo-H2A::3xmCherry; +*. atos RNAi (v36080): *w-; srpHemo-Gal4, srpHemo-H2A::3xmCherry/ UAS-nlacz; CG9005 RNAi* (*v36080*)/+. pths RNAi (v36589): *w-; srpHemo-Gal4, srpHemo-H2A::3xmCherry/UAS-nlacz; pths RNAi* (*v36589*)/+. Rescue: *w-; srpHemo-Gal4, srpHemo-H2A::3xmCherry; CG9005 RNAi* (*v36080*)/*UAS-pths*$^{nls-::}$*FLAG::HA.* Rescue: *w-; srpHemo-Gal4, srpHemo-H2A::3xmCherry; pths RNAi* (*v36589*)/*UAS-pths*$^{nls-}$*::FLAG::HA.*

Appendix Figure S3

Appendix Fig S3B-H: Control: *w-; +; srpHemo-3xmCherry*. atos mutant: *w-; atossa*^{BG02278}*; srpHemo-3xmCherry.*
