# Supplementary material for: Macrophage mitochondrial bioenergetics and tissue invasion are boosted by an Atossa‐Porthos axis in Drosophila
Source: EMBO J. 2022 Mar 23;41(12):e109049. doi: 10.15252/embj.2021109049 (PMC9194793; doi:10.15252/embj.2021109049)
Supplement: Supplementary file 2 — Expanded View Figures PDF [file EMBJ-41-e109049-s011.pdf]

Expanded View Figures

Figure EV1. *CG9005^{PBG}* mutant macrophages migrate normally within the head and along the vnc.

A, B Quantification of macrophages in fixed early Stage 12 embryos shows a significant increase on the yolk in (A) lines expressing each of the CG9005 RNAis specifically in macrophages (*mac>)* and in (B) the P element mutant *CG9005^{PBG}* compared to the control. For (A) control (n = 43 embryos) versus *CG9005^{PB}* mutant (n = 50), *CG9005^{PB}* mutant/*Df1* (n = 28) or *CG9005^{PB}* mutant/*Df2* (n = 9), all P < 0.0001. Control versus *CG9005^{PB}* mutant with *mac>CG9005* rescue (n = 20) P = 0.99. *CG9005^{PB}* mutant alone versus, mutant with *mac>CG9005* rescue P = 0.001. For (B) control 1 (n = 21 embryos) versus *CG9005 RNAi* 1 (n = 20) P = 0.0002; control 2 (n = 25) versus *CG9005 RNAi* 2 (n = 19) P < 0.0001; control 3 (n = 16) versus *CG9005 RNAi* 3 (n = 15) P = 0.001.

C–F Macrophage quantification in ventral nerve cord (vnc) segments reveals no significant difference in macrophage migration along the vnc between (C) *CG9005^{PBG}* mutant and control embryos or (D-F) *mac>CG9005 RNAi* embryos compared to the controls. For (C) control (n = 7 embryos) versus *CG9005^{PB}* mutant (n = 15) P > 0.05. For (D) control 1 (n = 8 embryos) versus *CG9005 RNAi* 1 (n = 13) P = 0.25; for (E) control 2 (n = 8 embryos) versus *CG9005 RNAi* 2 (n = 16) P = 0.5; for (F) control 3 (n = 8 embryos) versus *CG9005 RNAi* 3 (n = 16) P > 0.99.

G, H Quantification of the total macrophage number reveals no significant difference between (G) the control and *CG9005^{PBG}* mutant embryos, or (H) the control and *mac>CG9005 RNAi* embryos. For (G) control (n = 43 embryos) versus *CG9005^{PBG}* mutant (n = 50) P = 0.69. For (H) control 1 (n = 12 embryos) versus *CG9005 RNAi* 1 (n = 17) P = 0.9; control 2 (n = 27) versus *CG9005 RNAi* 2 (n = 19) P = 0.84; control 3 (n = 23) versus *CG9005 RNAi* 3 (n = 27) P = 0.16.

I Stills from two-photon movies of control and *CG9005^{PBG}* mutant embryos, showing macrophages migrating starting at Stage 10 from the head toward the germband. Elapsed time indicated in minutes. The germband edge (white dotted line) was detected by yolk autofluorescence. For quantification of migration parameters from two-photon live imaging of macrophages, see (J-L).

J Macrophages on the yolk sac in the *CG9005^{PBG}* mutant reach the germband with a similar speed to control macrophages. Speed: control and mutant = 2.2 μm/min, P = 0.78; control n = 8 movies, mutant n = 3; control n = 373 tracks, mutant n = 124.

K, L Macrophage directionality (K) in the head or (L) on the yolk sac shows no change in the *CG9005^{PBG}* mutant compared to the control. For (K) head directionality: control = 0.39, mutant = 0.37, P = 0.74; control n = 7 movies, mutant n = 3. For (L) yolk sac directionality: control = 0.40, mutant = 0.39, P = 0.86; control n = 7 movies, mutant n = 3.

Data information: Macrophages analyzed in (A-L) were labeled with *srpHemo-H2A::3xmCherry* to visualize nuclei. In schematics, macrophages are shown in red and analyzed macrophages in light blue, the ectoderm in green, the mesoderm in purple, and the yolk in beige. Throughout this work *mac>* indicates *srpHemo-GAL4* driving UAS constructs specifically in macrophages. Mean ± SEM, ns=P > 0.05, **P < 0.01, ***P < 0.001, ****P < 0.0001. One-way ANOVA with Tukey (A) and unpaired t-test (B-H) and (J-L). Scale bar: 30 μm (I). See Source Data 1 and 2 for Fig EV1.
Source data are available online for this figure.

© 2022 The Authors

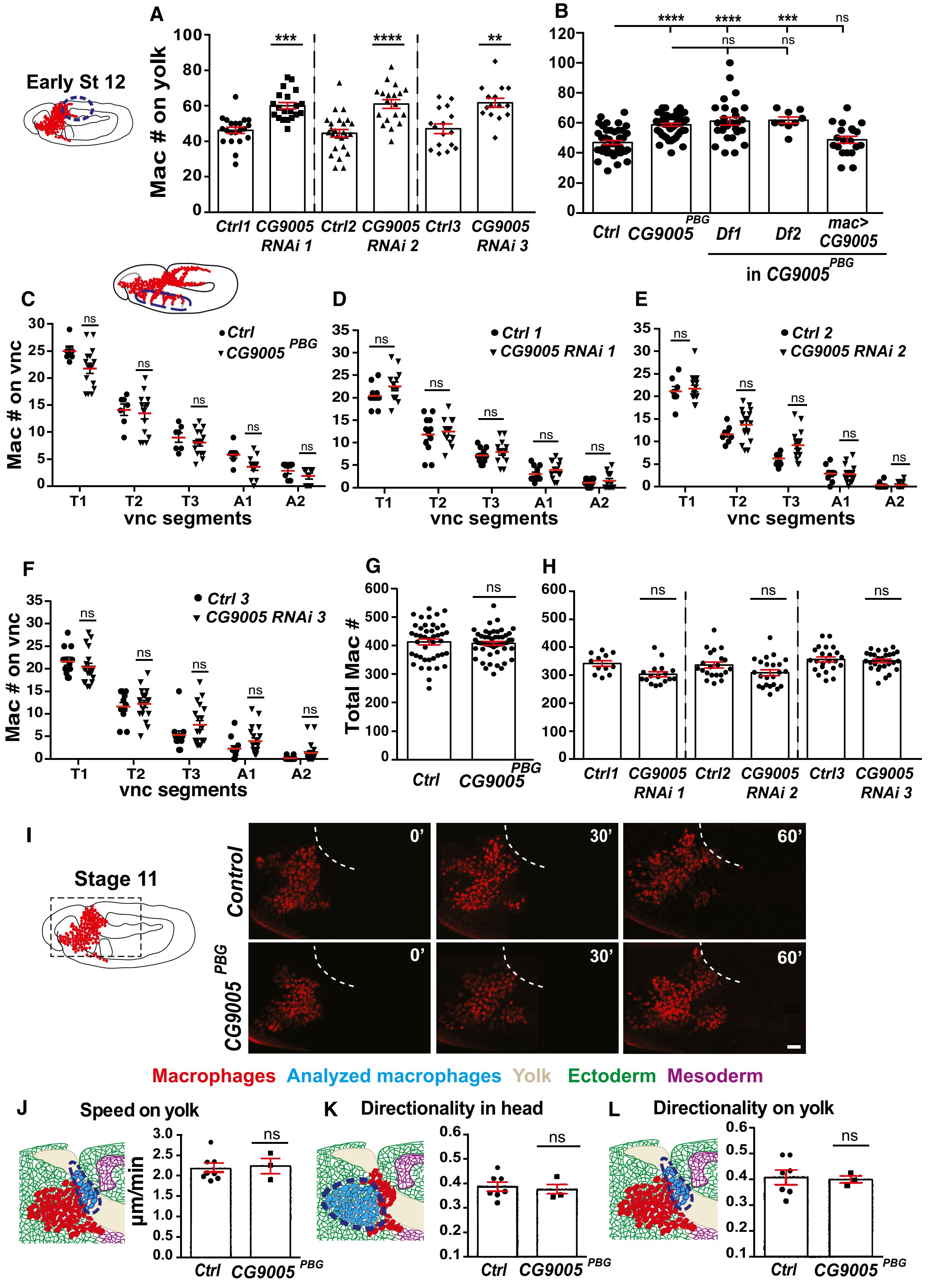
A
Early St 12
Mac # on yolk
Ctrl1 CG9005 RNAi 1 Ctrl2 CG9005 RNAi 2 Ctrl3 CG9005 RNAi 3
B
Ctrl CG9005 PBG Df1 Df2 mac> CG9005
in CG9005 PBG
C
Mac # on vnc
Ctrl
CG9005 PBG
vnc segments
D
Ctrl 1
CG9005 RNAi 1
E
Ctrl 2
CG9005 RNAi 2
F
Ctrl 3
CG9005 RNAi 3
G
Total Mac #
Ctrl CG9005 PBG
H
Ctrl1 CG9005 RNAi 1 Ctrl2 CG9005 RNAi 2 Ctrl3 CG9005 RNAi 3
I
Stage 11
Control
CG9005 PBG
0'
30'
60'
Macrophages Analyzed macrophages Yolk Ectoderm Mesoderm
J Speed on yolk
µm/min
K Directionality in head
L Directionality on yolk
Ctrl CG9005 PBG

Figure EV1.

Figure EV2. Atos's TAD domains are essential in macrophages for their tissue infiltration.

A S2R^{+} cells were transfected with wild-type Atos or forms lacking the indicated domains. N terminally HA-tagged Atos (green), the nuclear membrane marker Lamin (red), and the nucleolar marker Fibrillarin (red) were visualized with antibodies, and nuclear DNA with DAPI (blue). All forms of Atos are expressed under direct control of the macrophage-specific *srpHemo* promoter (*mac-*).

B Representative confocal images of Stage 12 embryos from $atos^{PBG}$ mutants expressing Atos lacking either TAD1 or 2 in macrophages. Macrophage nuclei (red) are visualized with a transgene and the embryo outlines with phalloidin staining (green). Germband edge: dotted white line.

C Quantification shows that deletion of TAD1 or 2 blocks Atos's ability to rescue the germband migration defect of Stage 12 $atos^{PBG}$ mutant embryos upon expression in macrophages. For control ($n = 32$ embryos) versus $atos^{PBG}$ mutant ($n = 56$) $P < 0.0001$; *mac-atos* rescue ($n = 18$) $P > 0.99$; $mac\text{-}atos^{TAD1-}$ rescue ($n = 32$) $P < 0.0001$; $mac\text{-}atos^{TAD2-}$ rescue ($n = 39$) $P = 0.008$. For $atos^{PBG}$ mutant versus *atos* rescue $P = 0.0006$, versus $atos^{TAD1-}$ rescue $P = 0.97$, versus $atos^{TAD2-}$ rescue $P = 0.06$.

D Quantification in fixed early Stage 12 embryos shows a significant increase compared to control embryos in the number of macrophages on the yolk in the mutant alone and in the $atos^{PBG}$ mutant expressing forms of *atos* lacking the DUF or Chrseg domains or both TAD domains. Significant difference was observed compared to the $atos^{PBG}$mutant only upon expression of wild-type *atos*. For control ($n = 43$ embryos) versus $atos^{PBG}$ mutant ($n = 50$) $P < 0.0001$; versus *atos* rescue ($n = 20$) $P > 0.99$; versus $atos^{DUF-}$ rescue ($n = 17$) $P = 0.0076$; versus $atos^{ChrSeg-}$ rescue ($n = 22$) $P = 0.0066$; versus $atos^{DUF-/ChrSeg-}$ rescue ($n = 27$) $P < 0.0001$; versus $atos^{TAD1-}$ rescue ($n = 18$) $P = 0.12$; versus $mac\text{-}atos^{TAD2-}$ rescue ($n = 24$) $P = 0.18$; versus $mac\text{-}atos^{TAD1-/\ TAD2-}$ rescue ($n = 18$) $P = 0.013$. For $atos^{PBG}$ mutant versus *atos* rescue $P = 0.0003$, versus $atos^{DUF-}$ rescue $P > 0.99$; versus $atos^{ChrSeg-}$ rescue $P > 0.99$; versus $atos^{DUF-/ChrSeg-}$ rescue $P > 0.99$, versus $atos^{TAD1-}$ rescue $P > 0.99$, versus $atos^{TA2-}$ rescue $P = 0.15$, versus $atos^{TAD1-/TAD2-}$ rescue $P > 0.99$.

E Quantification shows a similar number of macrophages on the yolk in fixed early Stage 12 $atos^{PBG}$ mutant embryos which express *mFAM214A* or *mFAM214B* in macrophages compared to the control. For control ($n = 43$ embryos) versus $atos^{PBG}$ mutant ($n = 50$) $P < 0.0001$; control versus *mac-mFAM214A* rescue ($n = 18$) $P = 0.65$; control versus *mac-mFAM214B* rescue ($n = 26$) $P = 0.56$; $atos^{PBG}$ mutant versus *mac-atos* rescue ($n = 20$), *mac-mFAM214A* and *mac-mFAM214B* rescues $P < 0.0001$.

Data information: Macrophages visualized with *srpHemo-H2A::3xmCherry* expression throughout mean ± SEM, ns = $P > 0.05$, *$P < 0.05$, **$P < 0.01$, ***$P < 0.001$, ****$P < 0.0001$. One-way ANOVA with Tukey (C-E). Scale bars: 3 µm (A), 50 µm (B). See Source Data 1 and 2 for Fig EV2.
Source data are available online for this figure.

 © 2022 The Authors

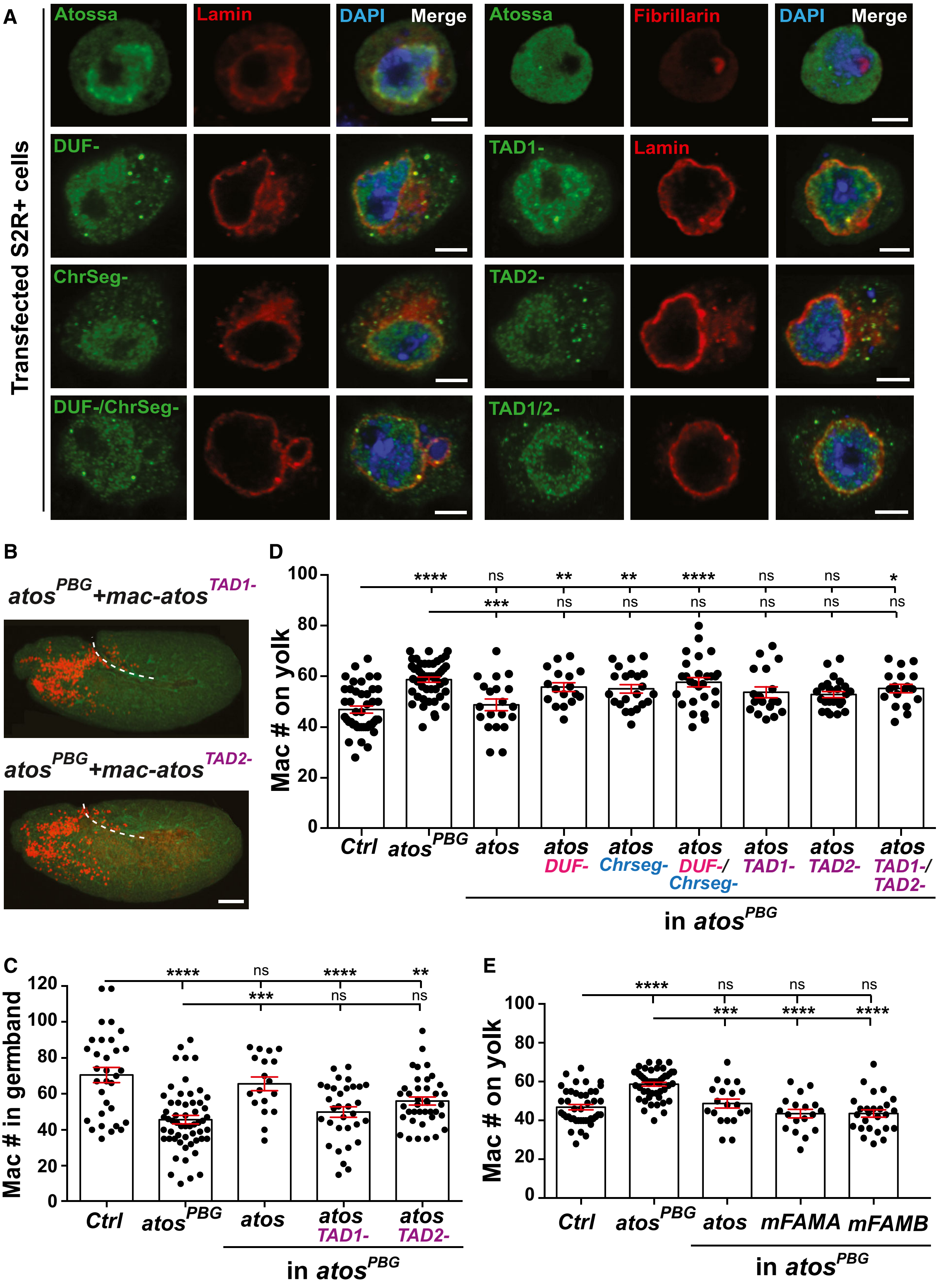
A
Transfected S2R+ cells
Atossa
Lamin
DAPI
Merge
Fibrillarin
DUF-
TAD1-
ChrSeg-
TAD2-
DUF-/ChrSeg-
TAD1/2-
B
atosPBG+mac-atosTAD1-
atosPBG+mac-atosTAD2-
C
Mac # in germband
Ctrl
atosPBG
atos
atos TAD1-
atos TAD2-
in atosPBG
D
Mac # on yolk
Ctrl
atosPBG
atos
atos DUF-
atos Chrseg-
atos DUF-/Chrseg-
atos TAD1-
atos TAD2-
atos TAD1-/TAD2-
in atosPBG
E
Mac # on yolk
Ctrl
atosPBG
atos
mFAMA
mFAMB
in atosPBG

Figure EV2.

Figure EV3. Macrophage transcriptome analysis reveals that Atos targets participate in signaling, cell communication, and ion transport.

A FACS plot of Side Scatter (SSC) versus mCherry fluorescence signal in macrophages obtained from embryos expressing *srpHemo-3xmCherry*. The two populations are sorted as mCherry marker + (red) and – (blue) cells.

B Genes expressed differentially in analysis of RNA sequencing data from macrophages from the $atos^{PBG}$ mutant compared to the control are shown in a volcano plot graphing the $\log_{10}$ of the *P*-value against the log fold change (FC) of the mean normalized expression levels. Each point represents the average value of one gene's expression from four replicate experiments. Dotted vertical lines indicate a $\log_{10}$ fold change ≥ 1 and the dotted horizontal line a *P*-value of ≤ 0.05. Statistically significant up- and downregulated genes are reported as red and green dots, respectively.

C Gene ontology (GO) analysis of downregulated genes from $atos^{PBG}$ mutant macrophages compared to the control shows that these genes are involved in oxidation-reduction processes, stress responses, as well as the nervous system.

D–F Quantification reveals that expression of RNAis against *porthos*, *LKR/SDH* and *GR/HPR*, in macrophages leads to a significant increase in macrophage numbers on the yolk in fixed early Stage 12 embryos compared to their controls. For (D) control ($n = 30$ embryos) versus *porthos* RNAi $n = 28$, (E) control 1 ($n = 27$) versus *LKR/SDH RNAi* 1 (VDRC 51346, $n = 17$) and control 2 ($n = 22$) versus *LKR/SDH RNAi* 2 (VDRC 109650, $n = 19$), all $P < 0.0001$. For (F) control 1 ($n = 27$) versus *GR/HPR RNAi* 1 (VDRC 44653, $n = 18$) $P = 0.0004$; control 2 ($n = 22$) versus *GR/HPR RNAi* 2 (VDRC 107680, $n = 24$) $P = 0.03$; control 3 ($n = 14$) versus *GR/HPR RNAi* 3 (VDRC 64652, $n = 21$) $P = 0.7$.

G, H Quantification in fixed early Stage 12 embryos reveals that knockdown by two different RNAis of (G) *Glycerophosphate oxidase* 2 (*Gpo2*, *CG2137*) or (H) *Golgi matrix protein 130 kD* (*GM130*, CG11061) did not change the macrophage number within the germband compared to their controls. For (G) control 1 ($n = 24$ embryos) versus *Gpo2 RNAi* 1 (VDRC 41234, $n = 11$) $P = 0.26$; control 2 ($n = 15$) versus *Gpo2 RNAi* 2 (VDRC 68145, $n = 27$) $P = 0.38$. For (H) control 1 ($n = 15$ embryos) versus *GM130 RNAi* 1 (VDRC 330284, $n = 25$) $P = 0.14$; control 2 ($n = 27$) versus *GM130 RNAi* 2 (VDRC 64920, $n = 20$) $P = 0.34$.

Data information: Mean ± SEM, ns=$P > 0.05$, *$P < 0.05$, ***$P < 0.001$, ****$P < 0.0001$. Unpaired *t*-test for (D-H). See Source Data 1 for Fig EV3.
Source data are available online for this figure.

 © 2022 The Authors

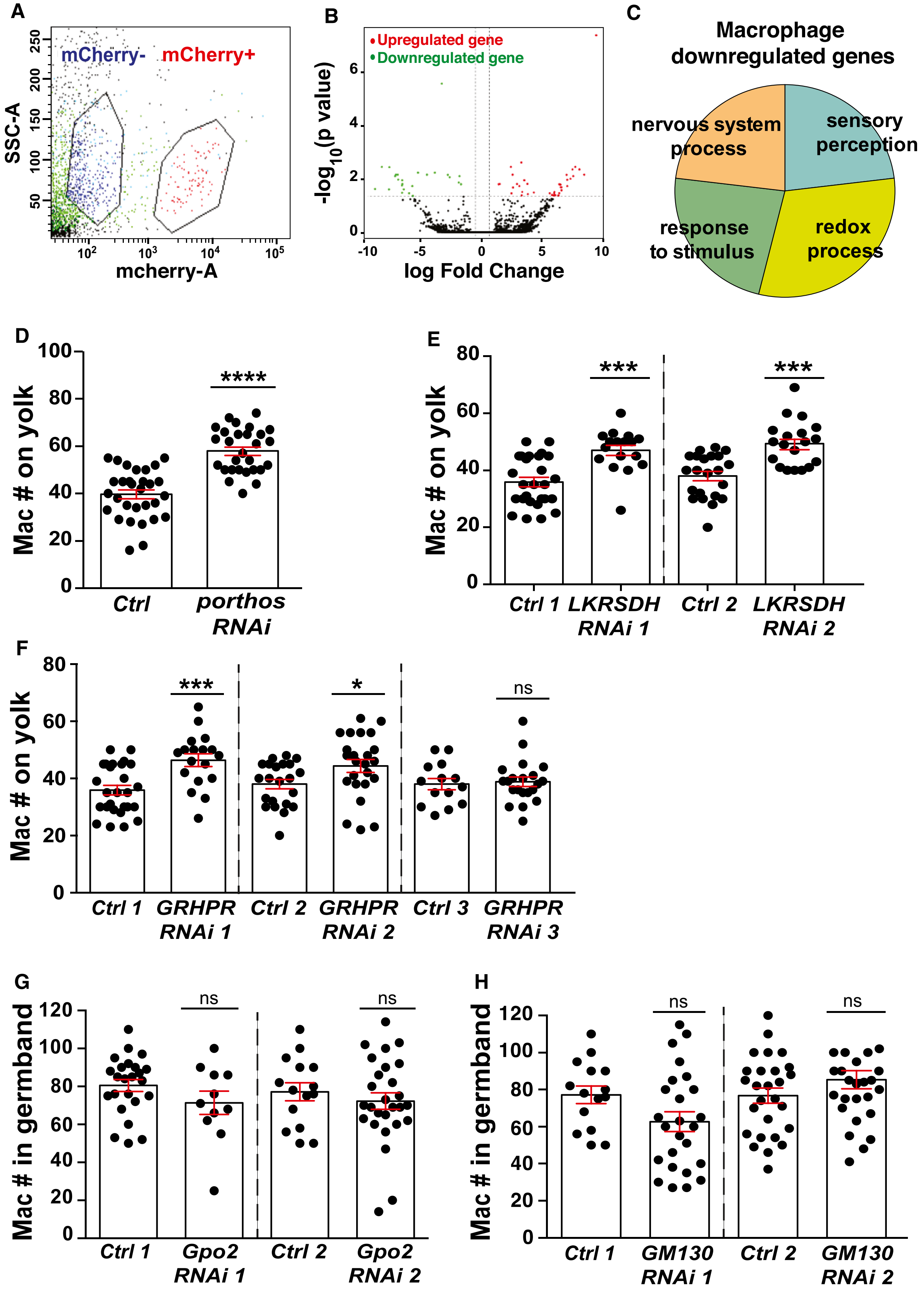
A
mCherry-
mCherry+
SSC-A
mcherry-A
B
Upregulated gene
Downregulated gene
$-log_{10}$(p value)
log Fold Change
C
Macrophage downregulated genes
nervous system process
sensory perception
response to stimulus
redox process
D
Mac # on yolk
****
Ctrl
porthos RNAi
E
***
***
Ctrl 1
LKRSDH RNAi 1
Ctrl 2
LKRSDH RNAi 2
F
***
*
ns
Ctrl 1
GRHPR RNAi 1
Ctrl 2
GRHPR RNAi 2
Ctrl 3
GRHPR RNAi 3
G
Mac # in germband
ns
ns
Ctrl 1
Gpo2 RNAi 1
Ctrl 2
Gpo2 RNAi 2
H
ns
ns
Ctrl 1
GM130 RNAi 1
Ctrl 2
GM130 RNAi 2

Figure EV3.

Figure EV4. Downregulation of *porthos* recapitulates the *atos* mutant phenotype.

A Deduced protein structure of Porthos (CG9253). Porthos contains two conserved motifs, a DEAD motif (Asp-Glu-Ala-Asp) and a Helicase C domain, as well as a predicted transactivation domain (TAD). *Drosophila* Porthos shows 71% identity and 84% similarity to its human ortholog, DDX47.

B Porthos (green) in $S2R^+$ cells transfected with *UAS-pths::HA* and *srpHemo-Gal4* and stained for the nuclear membrane marker Lamin (red), colocalizes with the staining for the nucleolar marker Fibrillarin (red), and DAPI (blue).

C, D Quantification of macrophage numbers in fixed Stage 12 embryos. Expression of *porthos* (*pths*) *RNAi* in macrophages has no effect in their numbers on (C) the vnc or (D) in the whole embryo compared to the control. For (C) control $n = 15$ embryos, *pths RNAi* $n = 15$, $P > 0.35$. For (D) control $n = 28$ embryos, *pths RNAi* $n = 20$, $P = 0.85$.

E Stills from two-photon movies of the migration of macrophages labeled with *srpHemo-H2A::3xmCherry* in control embryos and in those expressing *porthos RNAi* in macrophages.

F–H Macrophages from both genotypes have a similar (F) directionality in the head, and (G) speed and (H) directionality on the yolk sac, to control macrophages. For (F) directionality in head: control = 0.35, *pths RNAi* = 0.37; $P = 0.27$; control $n = 4$ movies, *pths RNAi* $n = 6$. For (G) speed on yolk sac: control = 2.10 μm/min, *pths RNAi* = 2.15; $P = 0.35$; control $n = 4$ movies, *pths RNAi* $n = 6$; control $n = 104$ tracks, *pths RNAi* $n = 168$. For (H) directionality on yolk: control = 0.42, *pths RNAi* = 0.39; $P = 0.58$; control $n = 3$ movies, *pths RNAi* $n = 6$.

I Macrophages (cytoplasm, red) expressing pths::FLAG::HA near the germband in Stage 11/12 $atos^{PBG}$ embryos show partial colocalization of the HA-antibody labeling Pths (green) with the nucleus (DAPI, blue). Pths expressed under *srpHemo-GAL4 UAS* control.

Data information: Mean ± SEM, ns=$P > 0.05$, Unpaired *t*-test for (C-D), and (F-H). Scale bars: 5 μm (B), 30 μm (E), and 50 μm (I). See Source Data 1 and 2 for Fig EV4. Source data are available online for this figure.

 © 2022 The Authors

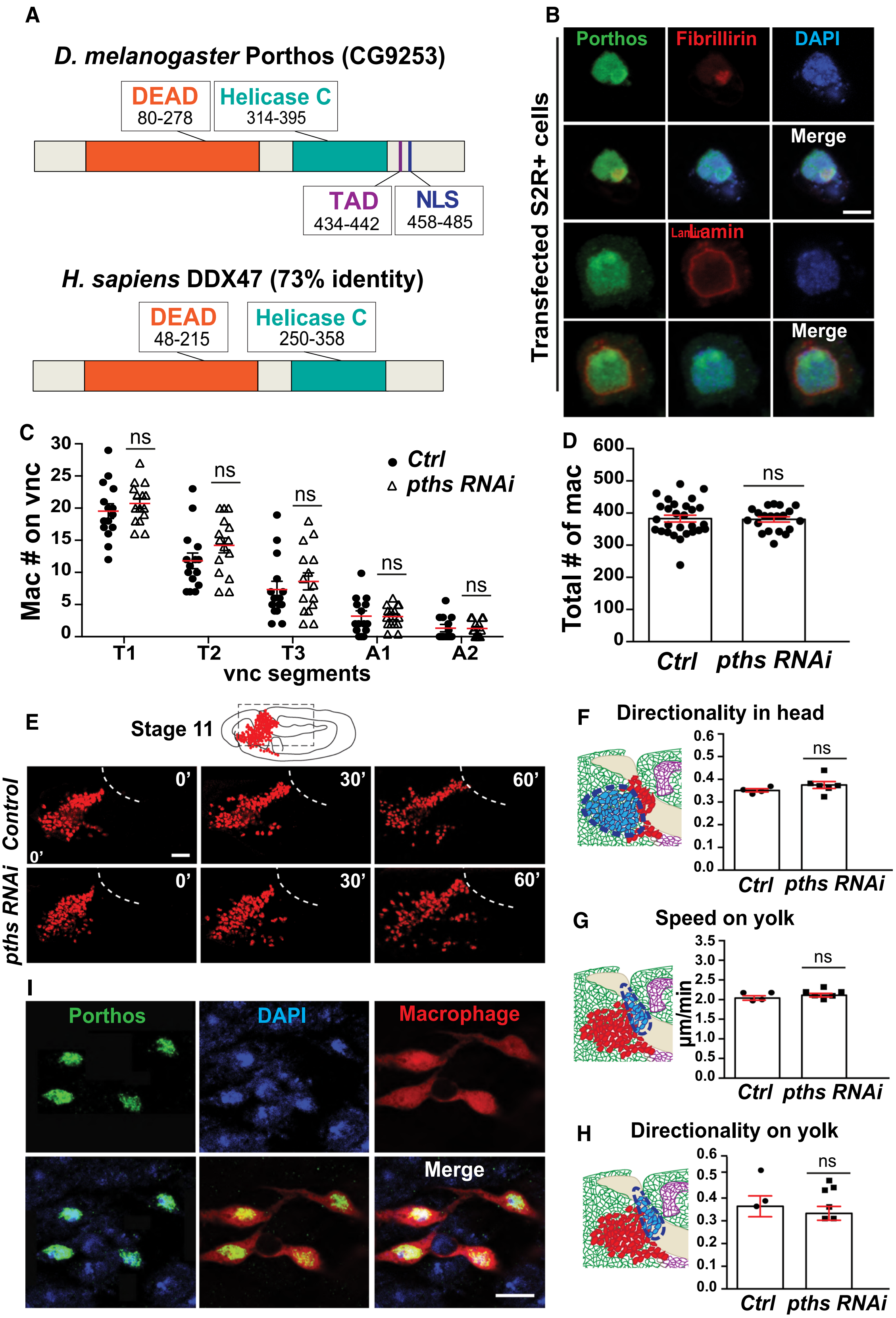
A
D. melanogaster Porthos (CG9253)
DEAD 80-278
Helicase C 314-395
TAD 434-442
NLS 458-485
H. sapiens DDX47 (73% identity)
DEAD 48-215
Helicase C 250-358
B
Transfected S2R+ cells
Porthos
Fibrillirin
DAPI
Merge
Lamin
Merge
C
Mac # on vnc
Ctrl
pths RNAi
ns
T1
T2
T3
A1
A2
vnc segments
D
Total # of mac
ns
Ctrl
pths RNAi
E
Stage 11
Control
pths RNAi
0'
30'
60'
F
Directionality in head
G
Speed on yolk
μm/min
H
Directionality on yolk
I
Porthos
DAPI
Macrophage
Merge

Figure EV4.

Figure EV5. Depletion of Atossa or Porthos mRNA causes impairment in mitochondrial metabolic activity, reduced ATP production, and a deficiency in macrophage tissue invasion.

A Schematic indicating the specific inhibitors (in red at right) used to block the function of mitochondrial OxPhos components. The glycolysis, TCA cycle, and mitochondrial respiratory chain in eukaryotic cells are shown.

B Graph shows relative *porthos* (*pths*) and *atos* mRNA levels in *pths KD* $S2R^+$ cells measured by qPCR from at least three independent experiments. The data are normalized to results for the internal control gene RpS20. *pths KD* $S2R^+$ cells contain 56% of normal *pths* mRNA levels and display a statistically insignificant decrease in *atos* mRNA levels. Control ($n = 6$ biological replicates) versus *pths* ($n = 6$), $P = 0.0002$, versus *atos* ($n = 3$), $P = 0.09$.

C The contribution of basal OxPhos ATP production rate and glycolytic ATP production rate were calculated. The plot shows that both wild-type and *pths KD* $S2R^+$ cells utilize OxPhos respiration as the predominant bioenergetic pathway to produce ATP in these cells; we observe no increase in the relative utilization of glycolysis.

D The relative basal values of the Oxygen Consumption rate (OCR), as a marker of OxPhos, and Extracellular Acidification Rate (ECAR), as an indication of glycolysis, in control and *pths KD* $S2R^+$ cells are plotted. Basal respiration rate is calculated before the addition of antimycin A. For OCR and ECAR assay, analysis is from values obtained in $n \geq 3$ independent biological experiments each with $n > 6$ technical replicate. Control versus *pths KD* for both assays: $P < 0.0001$.

E Graph shows mRNA levels relative to the control of the targeted gene in embryos ubiquitously expressing RNAis against subunits of mitochondrial Complexes III and V. Expression measured by qPCR and normalized to results for the internal control gene RpS20; $n = 3$ independent biological experiments. RNAi KD of *Complex III* resulted in 49% (RNAi 1), 24% (RNAi 2), and 32% (RNAi 3) of its normal mRNA levels; for *Complex V*, this was 50%. Control versus *Complex III RNAi 1*, $P = 0.0048$; versus *Complex III RNAi 2* $P = 0.0002$; versus *Complex III RNAi 3* $P = 0.0005$; versus *Complex V RNAi* $P = 0.005$

F Quantification in fixed early Stage 12 embryos shows a significant increase of macrophages on the yolk upon the expression in macrophages of any of three different *RNAis* against mitochondrial OxPhos *Complex III* (*UQCR*) or an *RNAi* against *Complex V* (*F1F0*, CG3612). For control ($n = 34$ embryos) versus *Complex III* (*Cyt-c1*, CG4769) *RNAi* 1 (VDRC 109809, $n = 19$) $P = 0.0049$; versus *Complex III* (*UQCR-cp1*, CG3731) *RNAi* 2 (VDRC 101350, $n = 18$) $P = 0.024$; versus *Complex III* (*UQCR-cp2*, CG4169) *RNAi* 3 (VDRC 100818, $n = 16$) $P = 0.009$; versus *Complex V* (*F1F0*, *CG3612*) *RNAi* (VDRC 34664, $n = 21$) $P = 0.0068$.

G, H Quantification of the number of macrophages in vnc segments does not show a significant change in general migration along the vnc in embryos whose macrophages express (G) *CV-DN* or (H) *RNAis* against mitochondrial OxPhos complex components compared to the control. For (G) control ($n = 20$ embryos) versus *CV-DN* ($n = 23$) $P > 0.05$. For (H) control ($n = 14$ embryos) versus *Complex III (Cyt-c1*, CG4769) *RNAi* 1 (VDRC 109809, $n = 10$) $P > 0.8$; versus *Complex III* (*UQCR-cp1*, CG3731) *RNAi* 2 (VDRC 101350, $n = 14$) $P > 0.05$; versus *Complex III* (*UQCR-cp2*, CG4169) *RNAi* 3 (VDRC 100818, $n = 11$) $P > 0.9$; versus *Complex V* (*F1F0*, CG3612) *RNAi* (VDRC 34664, $n = 18$) $P > 0.2$.

Data information: Ubiquitous expression of RNAis is through *da-GAL4*. Mean ± SEM, ns=$P > 0.05$, *$P < 0.05$, **$P < 0.01$, ***$P < 0.001$, ****$P < 0.0001$. Unpaired *t*-test followed by Sidak's correction (B,E). Unpaired *t*-test for (D-H). See Source Data 1 for Fig EV5.
Source data are available online for this figure.

© 2022 The Authors

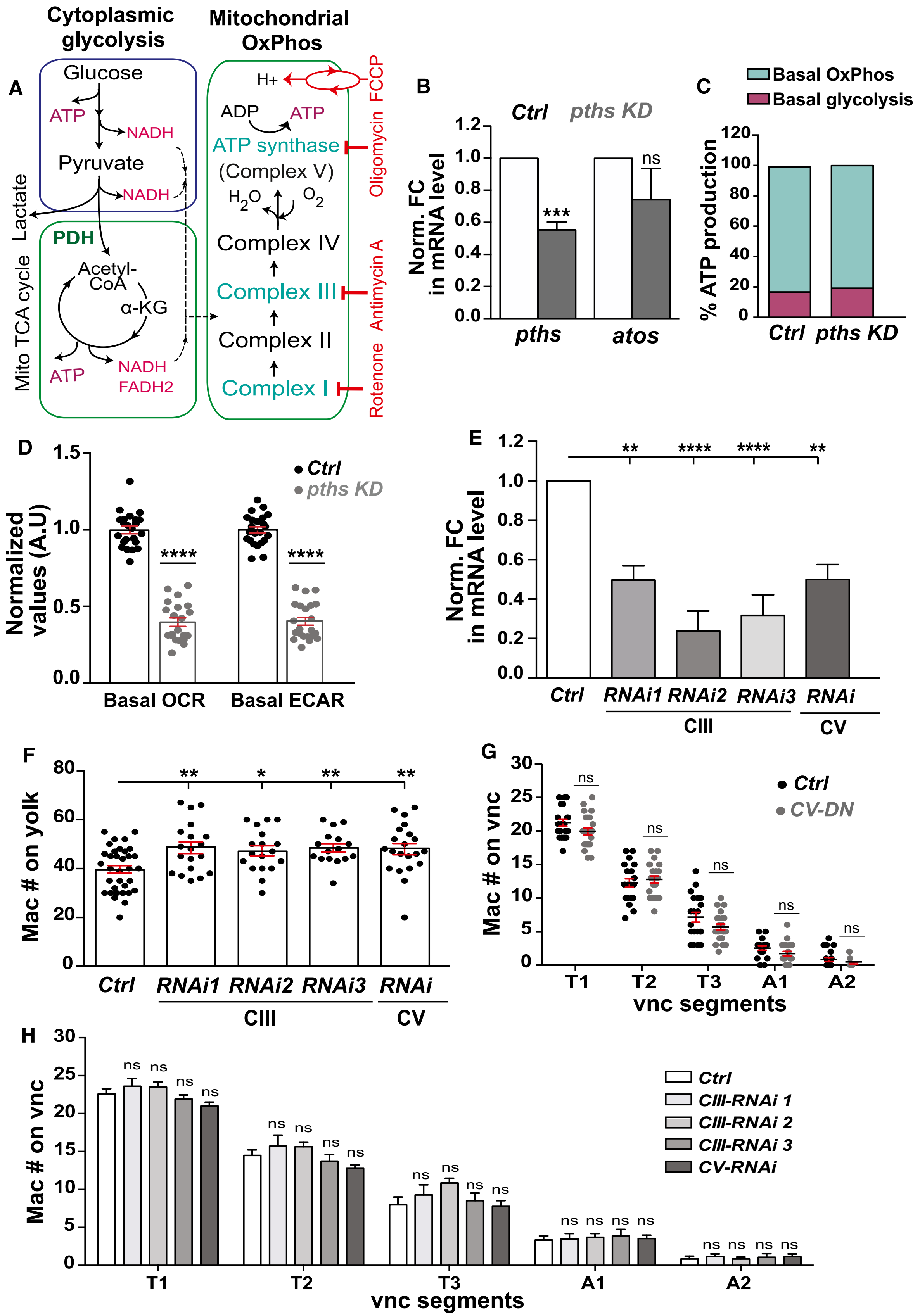
A
Cytoplasmic glycolysis
Mitochondrial OxPhos
Glucose
ATP
NADH
Pyruvate
NADH
Lactate
PDH
Acetyl-CoA
α-KG
Mito TCA cycle
ATP
NADH
FADH2
H+
FCCP
ADP
ATP
ATP synthase
(Complex V)
Oligomycin
H_2O
O_2
Complex IV
Complex III
Antimycin A
Complex II
Complex I
Rotenone
B
Ctrl
pths KD
Norm. FC in mRNA level
***
ns
pths
atos
C
Basal OxPhos
Basal glycolysis
% ATP production
Ctrl
pths KD
D
Normalized values (A.U)
Ctrl
pths KD
****
****
Basal OCR
Basal ECAR
E
Norm. FC in mRNA level
**
****
****
**
Ctrl
RNAi1
RNAi2
RNAi3
RNAi
CIII
CV
F
Mac # on yolk
**
*
**
**
Ctrl
RNAi1
RNAi2
RNAi3
RNAi
CIII
CV
G
Mac # on vnc
Ctrl
CV-DN
ns
T1
T2
T3
A1
A2
vnc segments
H
Mac # on vnc
Ctrl
CIII-RNAi 1
CIII-RNAi 2
CIII-RNAi 3
CV-RNAi
ns
T1
T2
T3
A1
A2
vnc segments

Figure EV5.
