# Supplementary material for: Macrophage mitochondrial bioenergetics and tissue invasion are boosted by an Atossa‐Porthos axis in Drosophila
Source: EMBO J. 2022 Mar 23;41(12):e109049. doi: 10.15252/embj.2021109049 (PMC9194793; doi:10.15252/embj.2021109049)
Supplement: Supplementary file 4 — Movie EV2 [file EMBJ-41-e109049-s010.zip › Movie EV2/EMBOJ-2021-109049R _MovieEV2_legend.docx]

**Movie EV2. (Related to Figures 1 and EV1) Representative movie of macrophage migration into the germband in the *CG9005^PBG^* (*atos*) mutant.**

Macrophages (red) are labeled with *srpHemo-H2A::3xmCherry*. Arrow shows first macrophage moving into the germband. Time interval between each acquisition is 40s, the display rate is 15 frames/s. Scale bar: 20μm.
