# Supplementary material for: Macrophage mitochondrial bioenergetics and tissue invasion are boosted by an Atossa‐Porthos axis in Drosophila
Source: EMBO J. 2022 Mar 23;41(12):e109049. doi: 10.15252/embj.2021109049 (PMC9194793; doi:10.15252/embj.2021109049)

Source Data related to Figure EV1

Fig. EV1I

Still mages were obtained from the two-photon movies of Control and *CG9005 mutant* embryos in xyz and 40X magnification. Stills were then cropped and the signal was adjusted in Fiji.

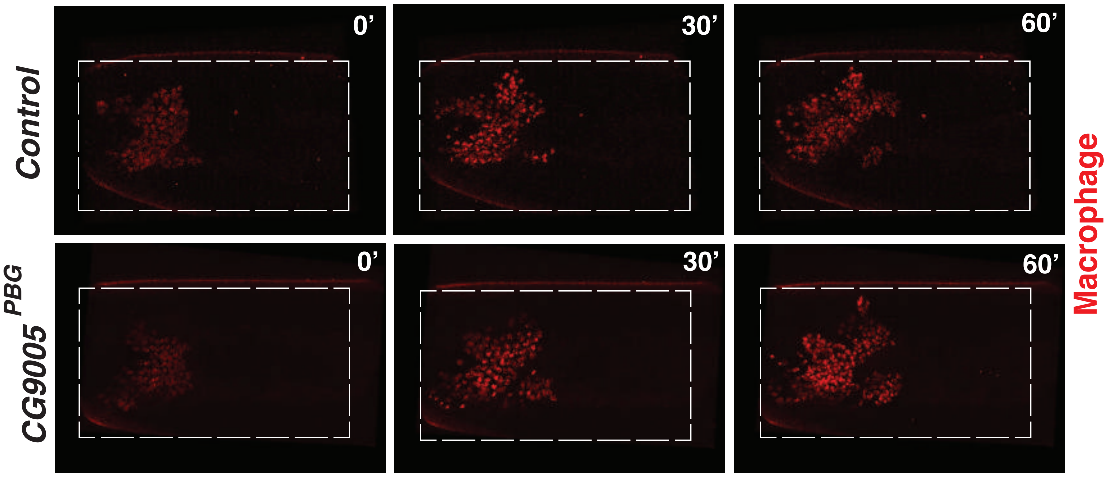

Supplement: Supplementary file 8 — Source Data for Expanded View and Appendix [file EMBJ-41-e109049-s016.zip › Appendix_and_EV_Figure_Source_Data/FigEV1_Source_Data/SourceData_2_for_Fig_EV1.pdf]
