# Supplementary material for: Macrophage mitochondrial bioenergetics and tissue invasion are boosted by an Atossa‐Porthos axis in Drosophila
Source: EMBO J. 2022 Mar 23;41(12):e109049. doi: 10.15252/embj.2021109049 (PMC9194793; doi:10.15252/embj.2021109049)

Source Data related to Figure EV4

Fig. EV4B
Confocal images of fixed S2R+ cells expressing Porthos-HA.
The signal and contrast were adjusted in Fiji.
The cropped images indicated with white boxes are shown in the paper.

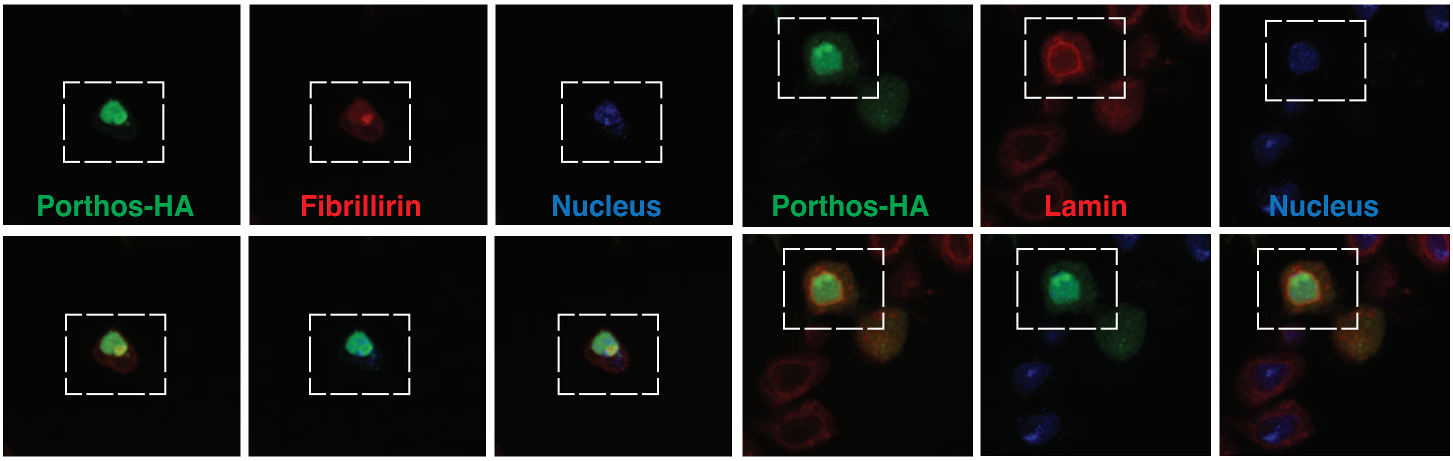

Fig. EV4E
Still images were obtained from the two-photon movies of *Control* and *porthos RNAi* embryos.
The signal and the contrast were adjusted in Fiji.
The cropped images indicated with white boxes are shown in the paper.

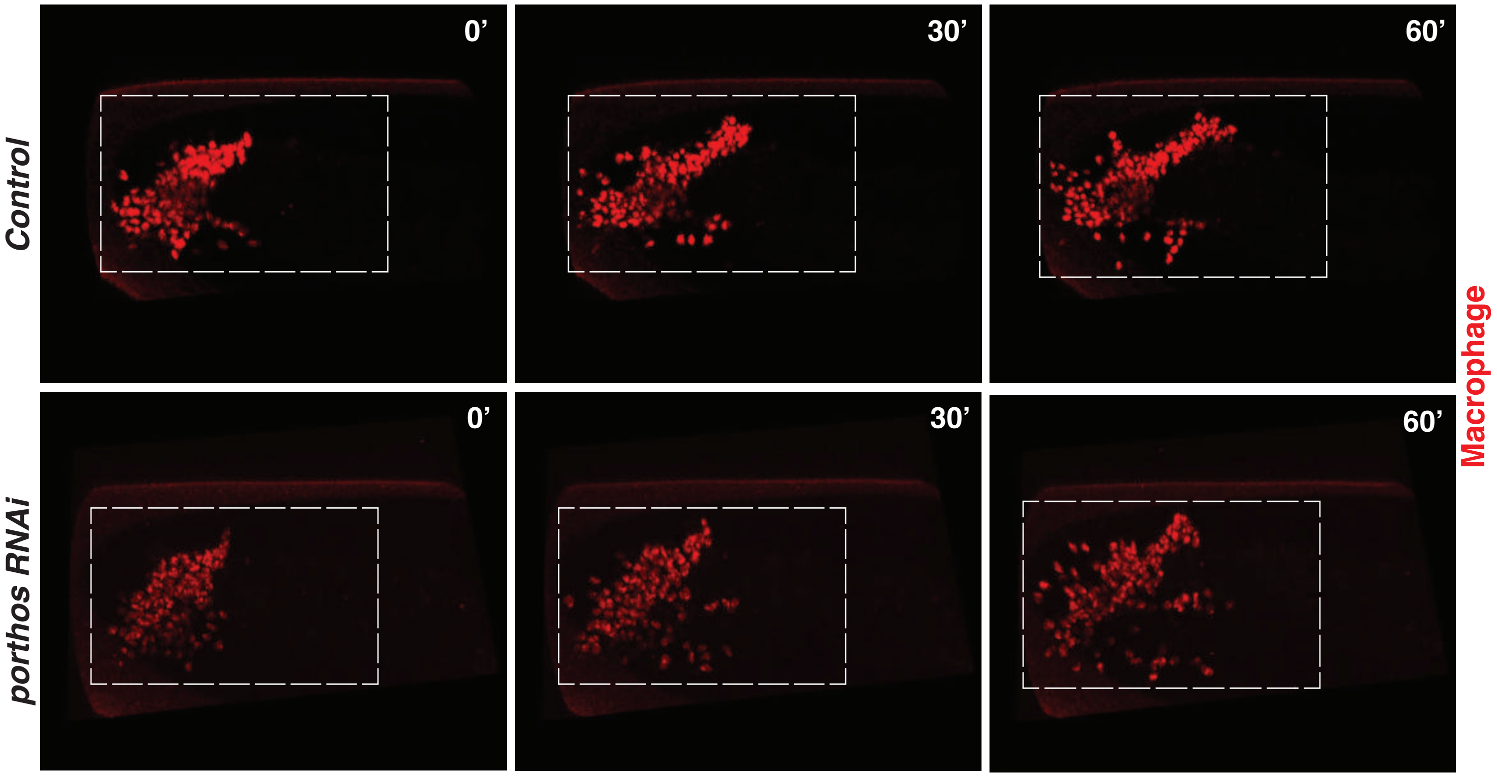

Supplement: Supplementary file 8 — Source Data for Expanded View and Appendix [file EMBJ-41-e109049-s016.zip › Appendix_and_EV_Figure_Source_Data/FigEV4_Source_Data/SourceData_2_for_Fig_EV4.pdf]
