# Supplementary material for: Macrophage mitochondrial bioenergetics and tissue invasion are boosted by an Atossa‐Porthos axis in Drosophila
Source: EMBO J. 2022 Mar 23;41(12):e109049. doi: 10.15252/embj.2021109049 (PMC9194793; doi:10.15252/embj.2021109049)

# Source Data related to Figure 1

**Figs. 1A,B,C**

Confocal images of embryos are obtained from the Z-projection of all slices of two channels. They were rotated, cropped and the signal was adjusted in Fiji.

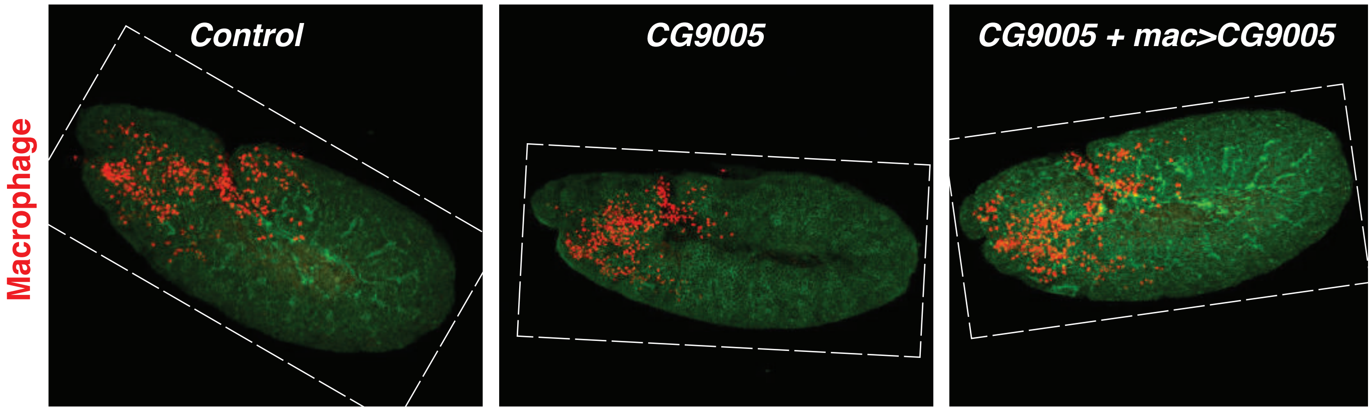


**Fig. 1F**

Still mages were obtained from the two-photon movies of Control and *CG9005 mutant* embryos in xyz and 40X magnification. Stills were then cropped and the signal was adjusted in Fiji.

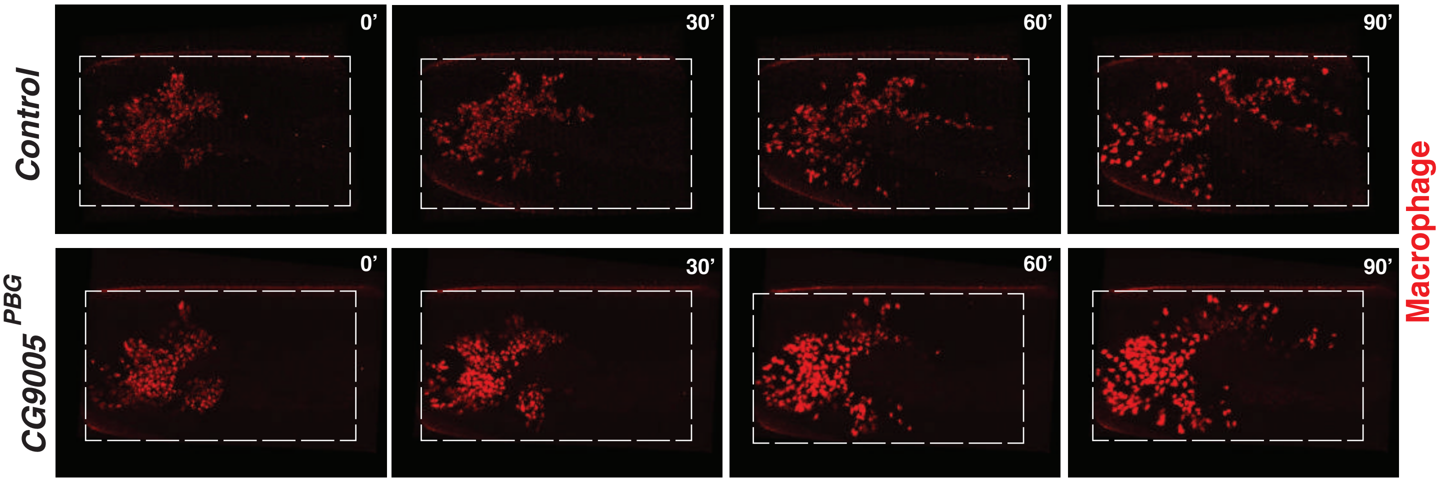

Supplement: Supplementary file 9 — Source Data for Figure 1 [file EMBJ-41-e109049-s004.zip › Fig1_Source_Data/SourceData_2_for_Fig_1.pdf]
