# Supplementary material for: Macrophage mitochondrial bioenergetics and tissue invasion are boosted by an Atossa‐Porthos axis in Drosophila
Source: EMBO J. 2022 Mar 23;41(12):e109049. doi: 10.15252/embj.2021109049 (PMC9194793; doi:10.15252/embj.2021109049)

Source Data related to Figure 2

Fig. 2B

Confocal images were obtained from the embryo expressing the Atossa-HA in macrophages.
A image plane was then rotated and cropped and the signal was adjusted in Fiji. The cropped area within the white box is shown in the paper.

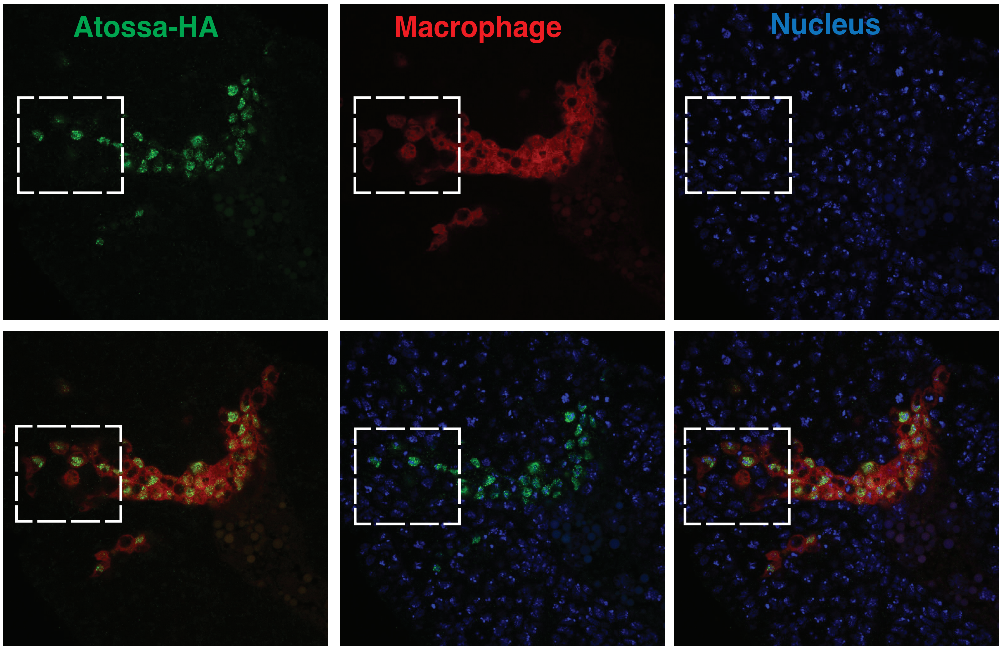

Figs. 2C, E

Confocal images are obtained from the Z-projection of all slices of two channels.
They were rotated, cropped and the signal was adjusted in Fiji.The cropped areas whitin the white box or the outlined areas were shown in the paper.

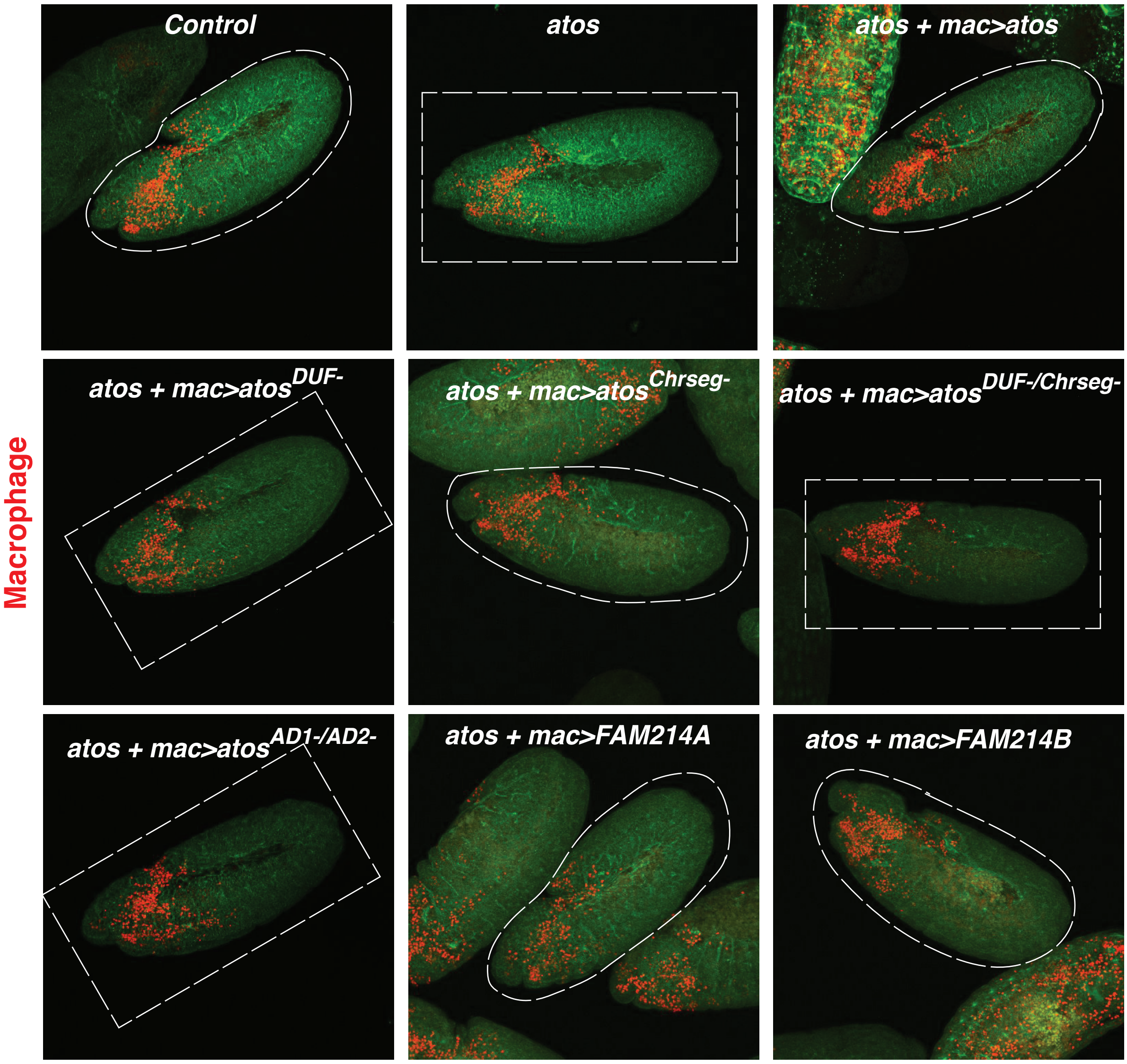

Supplement: Supplementary file 10 — Source Data for Figure 2 [file EMBJ-41-e109049-s015.zip › Fig2_Source_Data/SourceData_2_for_Fig_2.pdf]
