# Supplementary material for: Macrophage mitochondrial bioenergetics and tissue invasion are boosted by an Atossa‐Porthos axis in Drosophila
Source: EMBO J. 2022 Mar 23;41(12):e109049. doi: 10.15252/embj.2021109049 (PMC9194793; doi:10.15252/embj.2021109049)

Source Data related to Figure 4

Fig. 4A

Confocal images were obtained from the fixed embryo expressing the Porthos-HA in macrophages. A image plane was then rotated and cropped and the signal was adjusted in Fiji. The cropped area within the white box is shown in the paper.

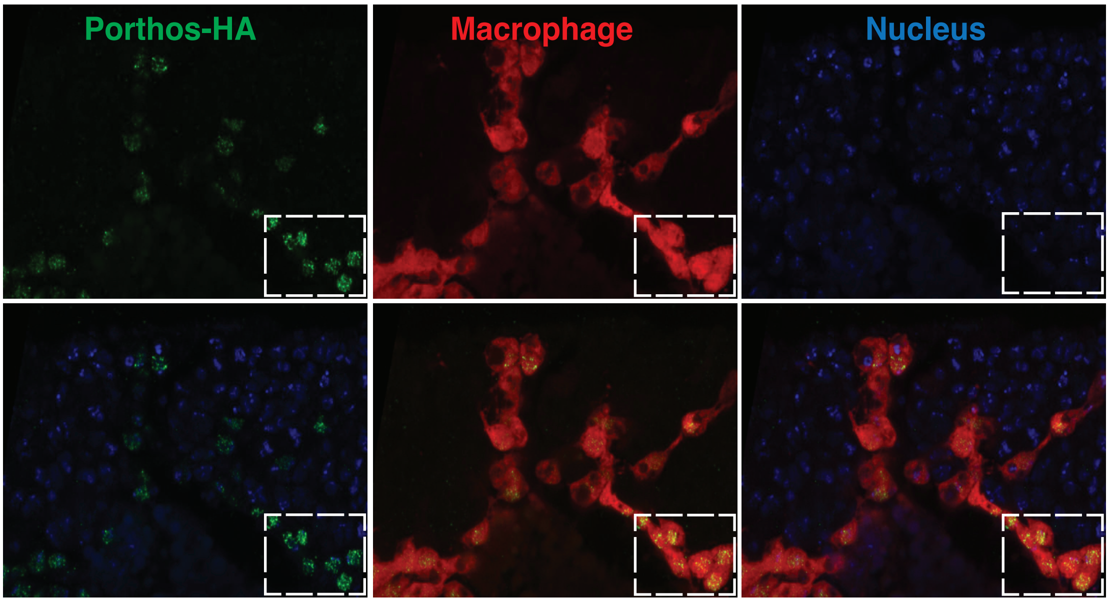

Fig. 4B

Images were obtained from the two-photon movies of Control and ***porthos RNAi*** embryos in xyz.
Stills were then cropped and the signal for the best contrast was adjusted in Fiji.
The cropped area whitin the white box was shown in the paper.

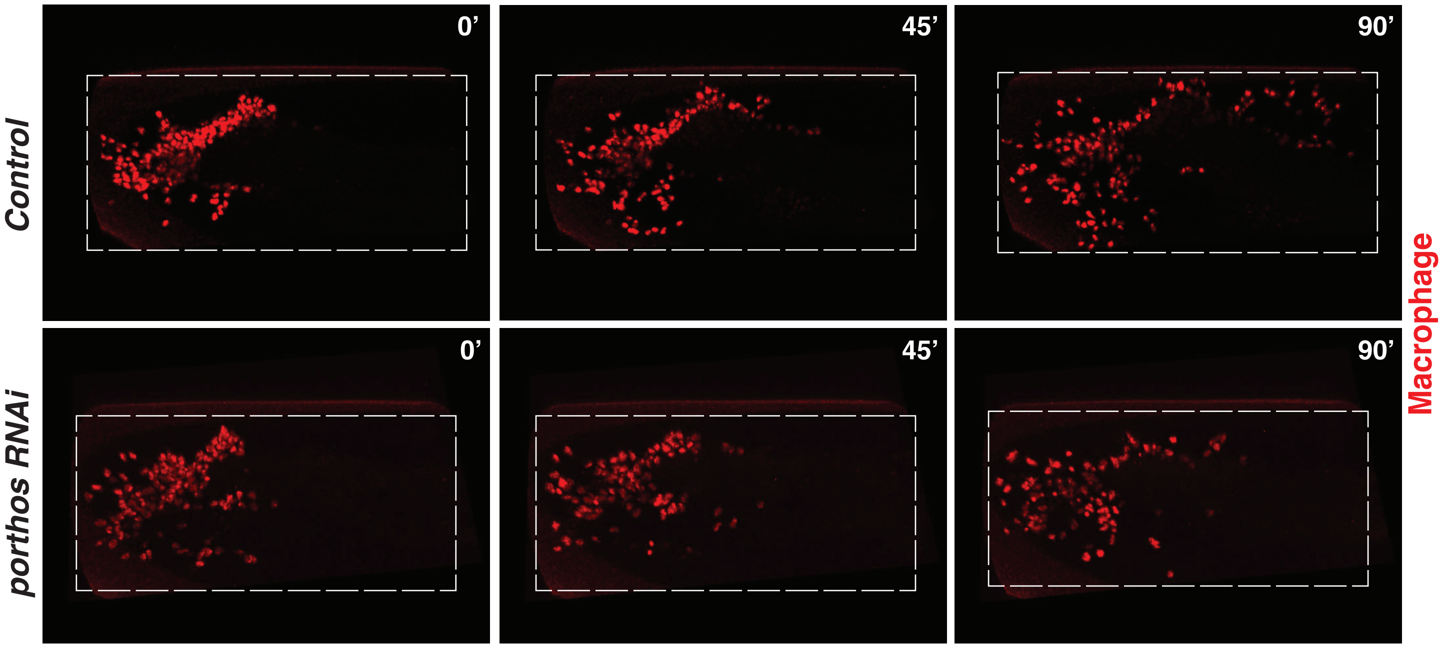

Fig. 4I

Confocal images of fixed embryos are obtained from the Z-projection of all slices of two channels.
They were rotated, cropped and the signal for the best contrast was adjusted in Fiji. The cropped areas whitin the white box or the outlined areas were shown in the paper.

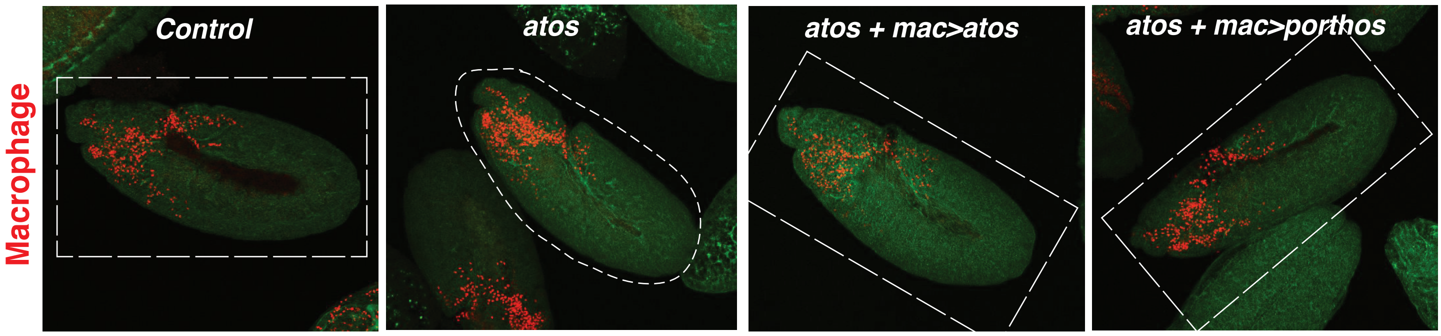

Supplement: Supplementary file 12 — Source Data for Figure 4 [file EMBJ-41-e109049-s002.zip › Fig4_Source_Data/SourceData_2_for_Fig_4.pdf]
