# Supplementary material for: Macrophage mitochondrial bioenergetics and tissue invasion are boosted by an Atossa‐Porthos axis in Drosophila
Source: EMBO J. 2022 Mar 23;41(12):e109049. doi: 10.15252/embj.2021109049 (PMC9194793; doi:10.15252/embj.2021109049)
Supplement: Supplementary file 13 — Source Data for Figure 5 [file EMBJ-41-e109049-s017.zip › SourceData_2_for_Fig_5.pdf]

Source Data related to Figure 5

Gels are the source for data shown in **Figure 5I**.
The first 6 wells were used for quantification.

p-4EBP1 (15-20 kDa)

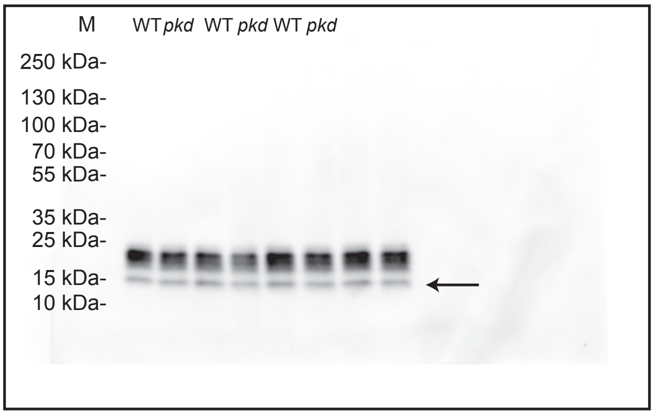

Tubulin β (55 kDa)

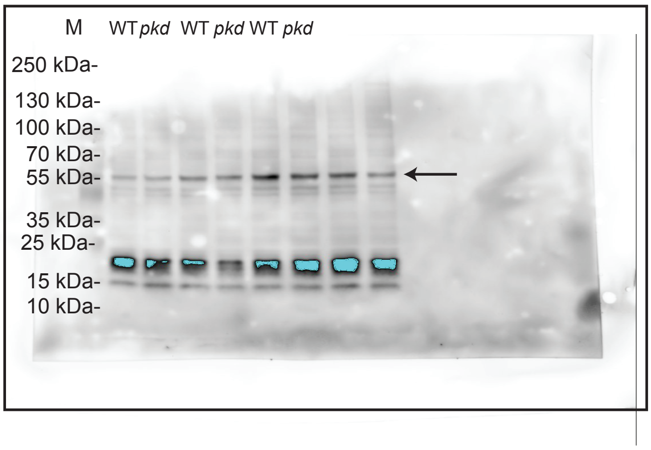

Method:
Chemilumeinescence was recorded via a ChenieDoc MP (BioRad) Molecular Imager and the related bands were densitometrically analyzed with ImaheJ.
M: Protein marker, WT: wildtype or control, pkd: porthos KD
