# Supplementary material for: Macrophage mitochondrial bioenergetics and tissue invasion are boosted by an Atossa‐Porthos axis in Drosophila
Source: EMBO J. 2022 Mar 23;41(12):e109049. doi: 10.15252/embj.2021109049 (PMC9194793; doi:10.15252/embj.2021109049)
Supplement: Supplementary file 14 — Source Data for Figure 6 [file EMBJ-41-e109049-s008.zip › Fig6_Source_Data/SourceData_2_for_Fig_6.pdf]

Source Data related to Fig 6

Fig. 6G

Gels are the source for data shown in **Figure 6G**.

MT-ND1 (CI) (~70 kDa)

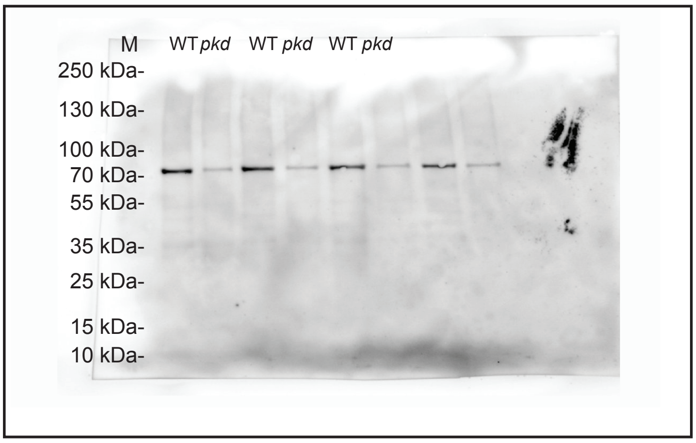

profilin (13.7 kDa)

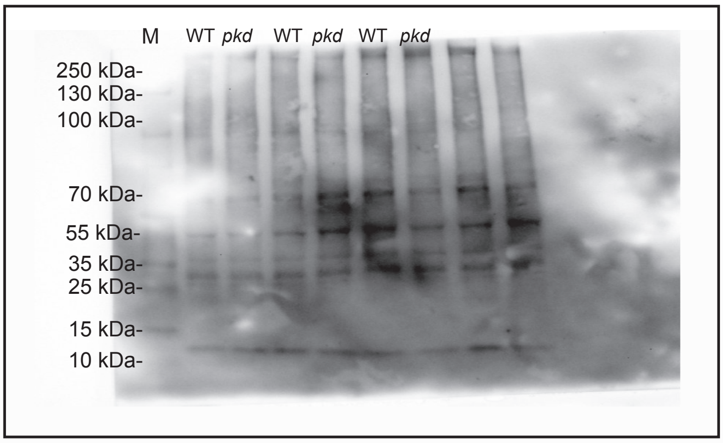

Fig. 6H

Gels are the source for data shown in **Figure 6H**.
The first 6 wells were used for quantification.

ATPsynt-β (CV) (57 kDa)

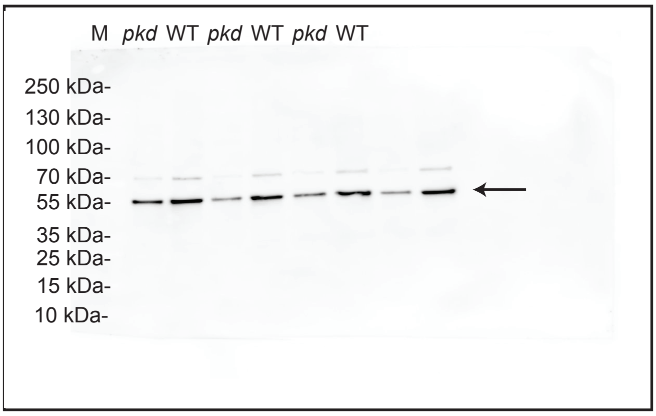

profilin (13.7 kDa)

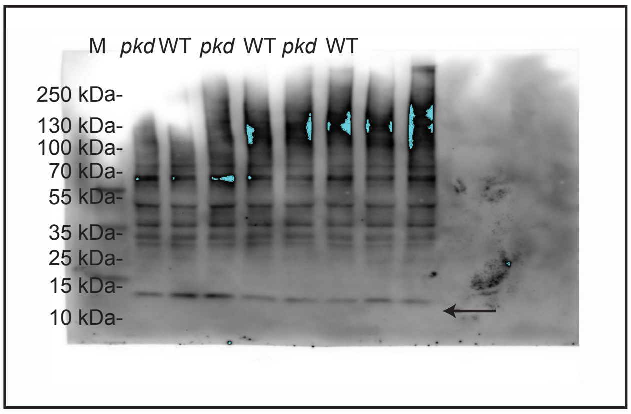

Fig. 6I

Gels are the source for data shown in **Figure 6I**.
The first 6 wells were used for quantification.

Tubulin β (~50 kDa)

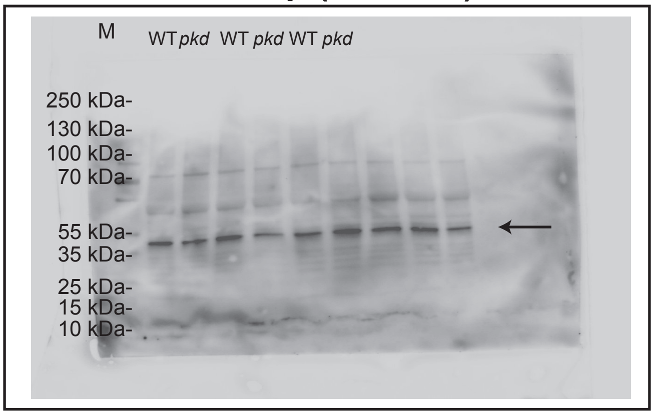

profilin (13.7 kDa)

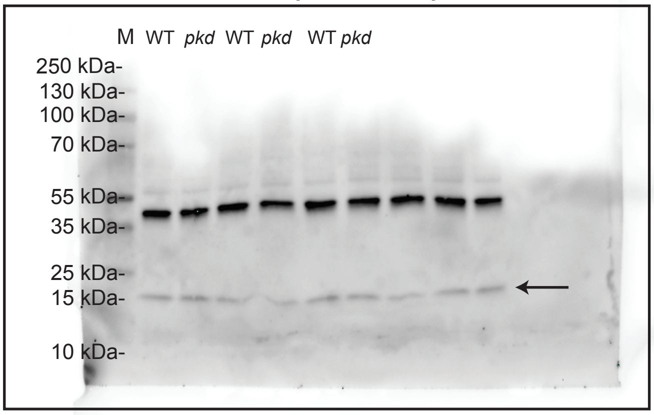

Method:
Chemilumeinescence was recorded via a ChenieDoc MP (BioRad) Molecular Imager and the related bands were densitometrically analyzed with ImaheJ.
M: Protein marker, WT: wildtype or control, pkd: porthos KD
