# Supplementary material for: Macrophage mitochondrial bioenergetics and tissue invasion are boosted by an Atossa‐Porthos axis in Drosophila
Source: EMBO J. 2022 Mar 23;41(12):e109049. doi: 10.15252/embj.2021109049 (PMC9194793; doi:10.15252/embj.2021109049)
Supplement: Supplementary file 18 [file EMBJ-41-e109049-s018.pdf]

THE EMBO JOURNAL

# Macrophage mitochondrial bioenergetics and tissue invasion are promoted by an Atossa-Porthos axis in *Drosophila*

Shamsi Emtenani, Elliot Martin, Attila György, Julia Bicher, Jakob-Wendelin Genger, Thomas Koecher, Maria Akhmanova, Mariana Guarda, Marko Roblek, Andreas Bergthaler, Thomas Hurd, Prashanth Rangan, and Daria Siekhaus
**DOI: 10.15252/embj.2021109049**

*Corresponding author: Daria Siekhaus (daria.siekhaus@ist.ac.at)*

## Review Timeline:

| | |
|---|---|
| Submission Date: | 24th Jun 21 |
| Editorial Decision: | 4th Aug 21 |
| Revision Received: | 22nd Dec 21 |
| Editorial Decision: | 1st Feb 22 |
| Revision Received: | 14th Feb 22 |
| Accepted: | 25th Feb 22 |

*Editor: Elisabetta Argenzio / Daniel Klimmeck*

## Transaction Report:

(Note: With the exception of the correction of typographical or spelling errors that could be a source of ambiguity, letters and reports are not edited. Depending on transfer agreements, referee reports obtained elsewhere may or may not be included in this compilation. Referee reports are anonymous unless the Referee chooses to sign their reports.)

Dear Dr. Siekhaus,

Thank you for submitting your manuscript entitled "A genetic program boosts mitochondrial function to power macrophage tissue invasion" [EMBOJ-2021-109049] to The EMBO Journal. Your study has now been assessed by three reviewers, whose reports are enclosed below.

As you can see, the referees concur with us on the potential interest of your findings. However, they also raise major critical points that need to be addressed before they can support publication here. In particular, referee #2 states that one of the key conclusions of the paper - i.e. Porthos regulates mRNA translation - is only based on polysome-Seq analysis. Therefore, this referee requests you to perform RT-qPCR analyses of selected (and representative) mRNAs identified as being translationally repressed across the polysome gradient. In addition, s/he asks you to show that protein levels encoded by such mRNAs are also altered. Referee #3 finds that additional mechanistic data are needed to support the main claims and asks you to investigate i) how LKR/SDH and GR/HPR regulate migration and mitochondrial bioenergetics; and ii) how Porthos/40S ribosome assembly defects affect nuclear-encoded mitochondrial gene translation. Furthermore, both referee #2 and #3 request you to test whether TORC1 activity is affected in Athos and Porthos deficient cells.

Given the overall interest of your study, I am pleased to invite submission of a manuscript revised as indicated in the reports attached herein. I would like to point it out that addressing all referees' points in a conclusive manner, as well as a strong and unanimous support from the reviewers, would be essential for publication in The EMBO Journal. I should also add that it is our policy to allow only a single round of major revision. Therefore, acceptance of your manuscript will depend on the completeness of your responses in this revised version.

We generally grant three months as standard revision time. As we are aware that many laboratories cannot function at full capacity owing to the COVID-19 pandemic, we may relax this deadline. Also, we have decided to apply our 'scooping protection policy' to the time span required for you to fully revise your manuscript and address the experimental issues highlighted herein. Nevertheless, please inform us as soon as a paper with related content is published elsewhere.

I realize that addressing all the referees' criticisms will require time and many additional experiments that might also be technically challenging. I would therefore understand if you were to choose not to undergo an extensive and risky revision here and rather pursue a submission elsewhere, in which case please inform us about your decision at your earliest convenience.

I have taken the liberty to discuss your study and the accompanying referees' reports with my colleague Deniz Senyilmaz Tiebe at our sister journal EMBO Reports. Deniz also finds your work potentially interesting and would be happy to discuss with you the requirements for publication in EMBO reports. Please contact her (d.senyilmaz@emboreports.org), if interested in this possibility or for any further questions regarding this matter.

When preparing your letter of response to the referees' comments, please bear in mind that this will form part of the Review Process File and will therefore be made available online. For more details on our Transparent Editorial Process, please visit our website: http://emboj.embopress.org/about#Transparent_Process.

When submitting your revised manuscript, please carefully review the instructions below and include the following items:

1) a .docx formatted version of the manuscript text (including legends for main figures, EV figures and tables). Please make sure that the changes are highlighted to be clearly visible.
2) individual production quality figure files as .eps, .tif, .jpg (one file per figure).
3) a .docx formatted letter INCLUDING the reviewers' reports and your detailed point-by-point response to their comments. As part of the EMBO Press transparent editorial process, the point-by-point response is part of the Review Process File (RPF), which will be published alongside your paper.
4) a complete author checklist, which you can download from our author guidelines (https://wol-prod-cdn.literatumonline.com/pb-assets/embosite/ Author Checklist%20-%20EMBO%20J-1561436015657.xlsx). Please insert information in the checklist that is also reflected in the manuscript. The completed author checklist will also be part of the RPF.
5) Please note that all corresponding authors are required to supply an ORCID ID for their name upon submission of a revised manuscript.
6) We require a 'Data Availability' section after the Materials and Methods. Before submitting your revision, primary datasets produced in this study need to be deposited in an appropriate public database, and the accession numbers and database listed under 'Data Availability'. Please remember to provide a reviewer password if the datasets are not yet public (see https://www.embopress.org/page/journal/14602075/authorguide#datadeposition). If no data deposition in external databases is needed for this paper, please then state in this section: This study includes no data deposited in external repositories. Note that the Data Availability Section is restricted to new primary data that are part of this study.*** Note - All links should resolve to a page where the data can be accessed. ***
7) When assembling figures, please refer to our figure preparation guideline in order to ensure proper formatting and readability in print as well as on screen: http://bit.ly/EMBOPressFigurePreparationGuideline. Please remember: Digital image enhancement

is acceptable practice, as long as it accurately represents the original data and conforms to community standards. If a figure has been subjected to significant electronic manipulation, this must be noted in the figure legend or in the 'Materials and Methods' section. The editors reserve the right to request original versions of figures and the original images that were used to assemble the figure.
8) For data quantification: please specify the name of the statistical test used to generate error bars and P values, the number (n) of independent experiments (specify technical or biological replicates) underlying each data point and the test used to calculate p-values in each figure legend. The figure legends should contain a basic description of n, P and the test applied. Graphs must include a description of the bars and the error bars (s.d., s.e.m.).
9) We would also encourage you to include the source data for figure panels that show essential data. Numerical data can be provided as individual .xls or .csv files (including a tab describing the data). For 'blots' or microscopy, uncropped images should be submitted (using a zip archive or a single pdf per main figure if multiple images need to be supplied for one panel). Additional information on source data and instruction on how to label the files are available at .
10) We replaced Supplementary Information with Expanded View (EV) Figures and Tables that are collapsible/expandable online (see examples in https://www.embopress.org/doi/10.15252/embj.201695874). A maximum of 5 EV Figures can be typeset. EV Figures should be cited as 'Figure EV1, Figure EV2" etc. in the text and their respective legends should be included in the main text after the legends of regular figures.
- For the figures that you do NOT wish to display as Expanded View figures, they should be bundled together with their legends in a single PDF file called *Appendix*, which should start with a short Table of Content. Appendix figures should be referred to in the main text as: "Appendix Figure S1, Appendix Figure S2" etc. See detailed instructions regarding expanded view here:
view >.
- Additional Tables/Datasets should be labelled and referred to as Table EV1, Dataset EV1, etc. Legends have to be provided in a separate tab in case of .xls files. Alternatively, the legend can be supplied as a separate text file (README) and zipped together with the Table/Dataset file.
11) Our journal encourages inclusion of *data citations in the reference list* to directly cite datasets that were re-used and obtained from public databases. Data citations in the article text are distinct from normal bibliographical citations and should directly link to the database records from which the data can be accessed. In the main text, data citations are formatted as follows: "Data ref: Smith et al,
2001" or "Data ref: NCBI Sequence Read Archive PRJNA342805, 2017". In the Reference list, data citations must be labeled with "[DATASET]". A data reference must provide the database name, accession number/identifiers and a resolvable link to the landing page from which the data can be accessed at the end of the reference. Further instructions are available at
.

I thank you again for the opportunity to consider this work for publication and look forward to your response.

Best regards,

Elisabetta

Elisabetta Argenzio, PhD
Editor
The EMBO Journal

Instructions for preparing your revised manuscript:

Please check that the title and abstract of the manuscript are brief, yet explicit, even to non-specialists.

IMPORTANT: When you send the revision we will require
- a point-by-point response to the referees' comments, with a detailed description of the changes made (as a word file).
- a word file of the manuscript text
- individual production quality figure files (one file per figure)
- a complete author checklist, which you can download from our author guidelines
(https://www.embopress.org/page/journal/14602075/authorguide).
- Expanded View files (replacing Supplementary Information)
Please see out instructions to authors
https://www.embopress.org/page/journal/14602075/authorguide#expandedview

Please remember: Digital image enhancement is acceptable practice, as long as it accurately represents the original data and conforms to community standards. If a figure has been subjected to significant electronic manipulation, this must be noted in the figure legend or in the 'Materials and Methods' section. The editors reserve the right to request original versions of figures and the original images that were used to assemble the figure.

Further information is available in our Guide For Authors: https://www.embopress.org/page/journal/14602075/authorguide

The revision must be submitted online within 90 days; please click on the link below to submit the revision online before 2nd Nov 2021.

https://emboj.msubmit.net/cgi-bin/main.plex

------------------------------------------------

Referee #1:

This is a beautiful study from Emtanani and colleagues that marries live imaging with the powerful genetics of Drosophila to dissect a novel mechanism by which immune cells are able to increase mitochondrial function to fuel invasive migration in vivo. By studying a subset of macrophages that carry out a characteristic invasive migration into the germ band of the developing embryo the authors identify the previously unstudied nuclear protein - Atossa as a key regulator of this invasive migration. They go on to tease apart the mechanism by which Atossa and a downstream target Porthos together increase the efficiency and amount of OxPhos within the macrophages to produce sufficient ATP to power tissue invasion. The manuscript is well written, the data is of extremely high quality throughout and support all the claims made in the paper. The findings are novel and high impact and the paper is highly appropriate for publication in The EMBO journal. The only question I would suggest the authors need to address is why atossa expression is maintained at a high level in all macrophages during stages 9-12 when it is only required within the first two pioneer macrophages in order to drive invasive migration into the germ band? Is this shift in metabolism required for or enhance other macrophage behaviours during development such as apoptotic cell clearance? Whilst this might be beyond the scope of this study to show experimentally, the authors should include in the discussion at least.

Referee #2:

Here the authors present a manuscript investing metabolic dynamics in the Drosophila immune system that govern how macrophages migrate and invade into the extended germband during development. They identify CG9005, which they rename Atossa, as being critical for early steps in pioneer macrophage invasion of the germband. They go on to characterize Atossa, find that it is a nuclear protein that appears to be a transcription factor. Importantly, they demonstrate that Atossa mutants lacking trans-activating domains does not rescue defects in Atossa deficient embryos, but that embryos can be rescued by expressing the murine ortholog mFAM214-A-B. RNA-Seq analysis of Atossa deficient macrophages identified a number of differentially expressed genes, with many downregulated genes being linked to mitochondrial dynamics (redox), as well as a gene coding for a predicted ATP-dependent RNA helicase (CG9253) that they name Porthos. They go on to show that Porthos localizes to both the nucleus and the nucleolus and show that knocking down Porthos also leads to a defect in macrophage migration into the germband. Based on its nucleolar localization, they carried out polysome profiling to investigate if Porthos impacts mRNA translation. A decrease in heavy polysomes and a concomitant increase in 60S subunit was observed in Porthos depleted cells. Polysome-Seq identified a number of genes that were translationally repressed, including many involved in mitochondrial respiration. Along these lines, they find that Porthos is required for mitochondrial respiration and energy production in order initiate germband invasion.

Overall, while I am not an expert in Drosophila development, I find that the paper is well-written, and the data clear and convincing for the most part. A major conclusion of the paper is that Porthos regulates mRNA translation. However, this is currently only based on polysome-Seq analysis. This should be subsequently validated by carrying out RT-qPCR of select mRNAs that they identify as being translationally repressed across the polysome gradient. This would allow the authors to see if they mRNA distribution does in fact shift from heavier fractions to lower ones. Finally, it would also be important to show that if translation is in fact being altered, that the levels of proteins encoded by these select mRNAs is also being altered. Mitochondrial dynamics are also tightly regulated by TORC1 signaling. Are they in a position to see whether TORC1 status (4E-BP protein phosphorylation) is being impacted in Porthos deficient cells? This could also potentially lead to a similar impact in mRNA translation of nuclear-encoded mitochondrial mRNAs. Finally, while a minor issue...on line 270: 'enhanced translational efficiency of a subset of proteins'. It should be of a subset of mRNAs, not proteins.

Referee #3:

Review for EMBOJ-2021-109049

Introduction

In this manuscript, Emtenani et al characterize novel regulators of cellular migration using Drosophila macrophages as a model system. The authors characterize the gene CG9005/Atossa, which is enriched in macrophages before germband entry. RNA

sequencing analyses uncovered a role of Atossa in driving gene expression of redox and stress responsive genes. Of the 5 most downregulated genes in Atossa mutants, 3 were required for germband migration including Porthos, a DEAD-box RNA helicase localized to the nucleolus and two metabolic enzymes LKR/SDH and GR/HPR. Density gradient fractionation experiments in S2R+ cells confirmed that Porthos deficiency leads to defects in ribosomal assembly and cytosolic translation. Ribosomal profiling experiments found 204 coding genes were less efficiently translated, including many mitochondrial genes, and 102 were more efficiently translated in Porthos mutants. The authors conclude that reduced translation of nuclear-encoded mitochondrial mRNAs from Atossa or Porthos deficiency causes mitochondrial dysfunction and prevents effective path invasion by leader cells.

This manuscript is interesting and so is their observation that ATP/mitochondrial metabolism is important for initial tissue invasion events by macrophages rather than general migration speed or directionality. Their overall conclusions need additional data to support the main claims, there are some mechanistic details missing from their manuscript, and at this point it seems preliminary. Namely, i) how LKR/SDH and GR/HPR regulate the migration phenotype is largely speculative and assumed to be related to mitochondrial bioenergetics and ii) how Porthos/40S ribosome assembly defects affect nuclear-encoded mitochondrial gene translation.

Major comments:

• Please determine if there are mitochondrial respiratory defects in LKR/SDH and GR/HPR mutants to solidify the connection of these genes to respiratory capacity. It is possible that these proteins do not play a role in mitochondrial respiratory capacity and ATP generation, and their role on migration is independent.
• There needs to be a better rationale of why ribosomal biogenesis defects cause specific mitochondrial defect instead of wide-spread issues with protein synthesis? Are the identified targets enriched for TISU elements that require cap-dependent translation? mRNAs with these structures usually encode mitochondrial proteins and are sensitive to mTORC1 inhibition. Is there strong overlap with your data set and others that study these mRNAs? See: 10.1016/j.cmet.2015.02.010 or 10.1101/gr.197566.115
This is an important point, and it is not clear how specific the translation is to mitochondrial proteins, as for example it has been described with the CLUH protein.
• Please determine if mTORC1 signaling (p-4EBP1) is suppressed during Atossa and Porthos LOF. This could help explain the specific defect in nuclear-encoded mitochondrial gene translation based mentioned above.
• In the introduction, there is no general principles that elevated mitochondria or PGC1s correlate with invasion, in fact in certain tumors high PGC1a and high mitochondria and oxidative phosphorylation correlate negatively with metastasis, as described by Carracedo's group in Nature Cell Biology (2016) and Cancer Research (2019).

Minor comments:

• In the context of Pths rescue in Atossa mutants, can you rescue any molecular phenotypes, e.g. translation defects, mitochondrial respiration, etc.
• Also, please verify that Pths overexpression is working in the Atossa mutants through qPCR or Western.
• As an alternative approach, can you rescue the ribosomal assembly defect in Atossa mutants to determine if this is responsible for downstream mitochondrial phenotypes? One could activate mTORC1 (pharmacological or genetic) to rescue mitochondrial defects present in Atossa mutants since mTORC1 controls ribosome assembly, mitochondrial gene translation, and PGC-1a activity. Or perhaps overexpress a ribosomal assembly factor that promotes 40S assembly.
• Can you express a mutant of Pths in Pths KD cells that fails to localize to the nucleus to assess whether its function in nucleolar ribosomal assembly is important for downstream phenotypes?
• Fig 6. b,c - For graphical representation of OCR in bar graph form, I would subtract non-mitochondrial respiration as a baseline correction. On that note, it is odd that your non-mitochondrial respiration is so different between genotypes. Perhaps this indicates a seeding/measurement issue since the cells are semi-adherent.
• Perform either SDS or BN-PAGE analyses for Atessa and Porthos LOFs to confirm nuclear-encoded translation defects.
• Please confirm efficacy of complex III and IV knockdowns by qPCR or Western.
• Is there any mRNA-mRNA or protein-protein correlation between Atossa and PGC-1a? Is there any overlap between Atossa and PGC1-a targets? This could strengthen arguments that these pathways can act in concert.

**Point by point response to reviewers comments on Atossa paper:**

**Reviewer 1)**

This is a beautiful study from Emtenani and colleagues that marries live imaging with the powerful genetics of Drosophila to dissect a novel mechanism by which immune cells are able to increase mitochondrial function to fuel invasive migration in vivo. By studying a subset of macrophages that carry out a characteristic invasive migration into the germ band of the developing embryo the authors identify the previously unstudied nuclear protein - Atossa as a key regulator of this invasive migration. They go on to tease apart the mechanism by which Atossa and a downstream target Porthos together increase the efficiency and amount of OxPhos within the macrophages to produce sufficient ATP to power tissue invasion. The manuscript is well written, the data is of extremely high quality throughout and support all the claims made in the paper. The findings are novel and high impact and the paper is highly appropriate for publication in The EMBO journal.

We greatly thank the reviewer for their compliments about our work and its impact.

1. The only question I would suggest the authors need to address is why atossa expression is maintained at a high level in all macrophages during stages 9-12 when it is only required within the first two pioneer macrophages in order to drive invasive migration into the germband?

2. Is this shift in metabolism required for or enhance other macrophage behaviours during development such as apoptotic cell clearance? Whilst this might be beyond the scope of this study to show experimentally, the authors should include in the discussion at least.

This is a very interesting question, and studying the effects of Atossa on immune responses is something we seek to tackle in the future. In this work, as the reviewer suggested, we added a section to the discussion in lines 564-570:

"At least at the RNA level Atossa is upregulated not just in the first two pioneer macrophages but in all of them. This may enable a large potential pool of macrophages to be capable of serving as the pioneers. Atossa may also support other energy intensive tasks such as apoptotic cell phagocytosis, (Borregaard and Herlin, 1982), a capacity carried out by most migrating macrophages to aid development (Tepass et al., 1994) which also primes their inflammatory responses (Weavers et al., 2016)."
…………………………………………………………………………………

**Reviewer 2)**

Here the authors present a manuscript investing metabolic dynamics in the Drosophila immune system that govern how macrophages migrate and invade into the extended germband during development. They identify CG9005, which they rename Atossa, as being critical for early steps in pioneer macrophage invasion of the germband. They go on to characterize Atossa, find that it is a nuclear protein that appears to be a transcription factor. Importantly, they demonstrate that Atossa mutants lacking trans-activating domains does not rescue defects in Atossa deficient embryos, but that embryos can be rescued by expressing the murine ortholog mFAM214-A-B. RNA-Seq analysis of Atossa deficient macrophages identified a number of differentially expressed genes, with many

downregulated genes being linked to mitochondrial dynamics (redox), as well as a gene coding for a predicted ATP-dependent RNA helicase (CG9253) that they name Porthos. They go on to show that Porthos localizes to both the nucleus and the nucleolus and show that knocking down Porthos also leads to a defect in macrophage migration into the germband. Based on its nucleolar localization, they carried out polysome profiling to investigate if Porthos impacts mRNA translation. A decrease in heavy polysomes and a concomitant increase in 60S subunit was observed in Porthos depleted cells. Polysome-Seq identified a number of genes that were translationally repressed, including many involved in mitochondrial respiration.
Along these lines, they find that Porthos is required for mitochondrial respiration and energy production in order initiate germband invasion.

Overall, while I am not an expert in *Drosophila* development, I find that the paper is well-written, and the data clear and convincing for the most part.

We very much appreciate the reviewer's positive feedback on the manuscript. We are also grateful to the reviewer for their thoughtful and critical reading of our manuscript, for the suggestions to improve it and for their questions. Addressing these points has greatly strengthened our study.

**1.** A major conclusion of the paper is that Porthos regulates mRNA translation. However, this is currently only based on polysome-Seq analysis. This should be subsequently validated by carrying out RT-qPCR of select mRNAs that they identify as being translationally repressed across the polysome gradient.

To validate our polysome profiling data we conducted RT-qPCR analysis on polysome profile fractions for a number of Porthos target transcripts in the control and *porthos-KD* S2R+ cells. We have assessed the fractions including RNP, 40S/60S, monosome, low polysome (di- and trisome), high polysome (remaining fractions) for 3 independent biological replicates. We normalized the data to the monosome fraction for each transcript.
We observed statistically significant decreases in the mRNA levels on high polysomes for mitochondrial OxPhos complexes I, III and V (Fig. 6C-E) and strong decreases in the light polysome fractions in *porthos-KD* S2R+ cells compared to the control. As our collaborator was moving his laboratory we were unable to obtain more samples to increase the significance further. We did not find a similar effect in these fractions when we tested GAPDH gene as a non-target control (Fig. 6F). Thus this RT-qPCR analysis confirms our previous polysome profiling results.

We present these results in lines 348-363 and show the data in Fig 6C-F.

**2.** Finally, it would also be important to show that if translation is in fact being altered, that the levels of proteins encoded by these select mRNAs is also being altered.

Unlike in mammalian systems, there are few available antibodies against *Drosophila* proteins. Thus we were unable to obtain antibodies corresponding to any of the direct targets we identified in the RNAseq. To get around this we utilized commercially available antibodies that had been validated for Drosophila (Teixeira et al 2015 PMID 25915123) against subunits of the OxPhos complexes whose protein levels had been shown to depend on the protein levels of our direct targets. Lower levels of the mammalian ortholog of the predicted complex I assembly factor we identified as a target in the RNAseq lead to reduced levels of other complex I proteins, including MT-ND1 (see Formosa et al., 2015

Fig 2C,D). Similarly, in humans the absence of subunit g of complex V, one of our targets, has been shown to lead to lower protein levels of multiple other subunits including ATP synt-β (see He et al., 2018 Fig 5A). In Western blots we found 73% and 31% lower levels of these CI and CV proteins respectively in *pths-KD* S2R+ cells compared to the control (CI MT-ND1, $p<0.0001$; CV ATP synt-β, $p=0.03$) (shown in Fig 6G-H). To test for a possible general deficiency in protein translation we examined the non-target proteins profilin and tubulin b and found no significant change in levels (Fig 6I) (profilin, $p=0.26$; β-tubulin, $p=0.55$). In sum, our results argue that Pths does not affect protein translation generally, but is required for the enhanced levels of a subset of proteins, many of which are involved in mitochondrial and metabolic function.

The results are presented in lines 364-382 and shown in Fig 6G-I.

**3.** Mitochondrial dynamics are also tightly regulated by TORC1 signaling. Are they in a position to see whether TORC1 status (4E-BP protein phosphorylation) is being impacted in Porthos deficient cells? This could also potentially lead to a similar impact in mRNA translation of nuclear-encoded mitochondrial mRNAs.

To address this and a related question from Reviewer #3 we assessed the activity of the dTORC1 signaling pathway examining the phosphorylation status of the TORC1 kinase target 4EBP1 in the control and *porthos-KD* S2R+ cells via Western Blot. We saw no significant change in the levels of p-4EBP1 in *porthos-KD* cells compared to the control (Fig. 5I), arguing that Atossa and Porthos affect the mRNA translation of a set of nuclear-encoded mitochondrial mRNAs through a mechanism independent of general translation controlled by dTORC1.
We present this data in lines 329-335 and Fig 5I.

**4.** Finally, while a minor issue...on line 270: 'enhanced translational efficiency of a subset of proteins'. It should be of a subset of mRNAs, not proteins.

We greatly thank the reviewer for catching our error. We have corrected the text accordingly in the abstract and changed the wording in previous line 270 to “is required for the enhanced levels of a subset of proteins” in current line 381 as we have now added more data to substantiate this conclusion.

……………………………………………………………………………

**Reviewer 3)**

**Introduction**
In this manuscript, Emtenani et al characterize novel regulators of cellular migration using Drosophila macrophages as a model system. The authors characterize the gene CG9005/Atossa, which is enriched in macrophages before germband entry. RNA sequencing analyses uncovered a role of Atossa in driving gene expression of redox and stress responsive genes. Of the 5 most downregulated genes in Atossa mutants, 3 were required for germband migration including Porthos, a DEAD-box RNA helicase localized to the nucleolus and two metabolic enzymes LKR/SDH and GR/HPR. Density gradient fractionation experiments in S2R+ cells confirmed that Porthos deficiency leads to defects in ribosomal assembly and cytosolic translation. Ribosomal profiling experiments found 204 coding genes were less efficiently translated, including many mitochondrial genes,

and 102 were more efficiently translated in Porthos mutants. The authors conclude that reduced translation of nuclear-encoded mitochondrial mRNAs from Atossa or Porthos deficiency causes mitochondrial dysfunction and prevents effective path invasion by leader cells.

This manuscript is interesting and so is their observation that ATP/mitochondrial metabolism is important for initial tissue invasion events by macrophages rather than general migration speed or directionality. Their overall conclusions need additional data to support the main claims, there are some mechanistic details missing from their manuscript, and at this point it seems preliminary.

Namely,

i) how LKR/SDH and GR/HPR regulate the migration phenotype is largely speculative and assumed to be related to mitochondrial bioenergetics?

ii) how Porthos/40S ribosome assembly defects affect nuclear-encoded mitochondrial gene translation?

We thank the reviewer for their positive comments on our study. We are also very grateful for the extremely thoughtful questions about the manuscript and the suggestions which we have implemented that have greatly improved the work.

**Major comments:**

• Please determine if there are mitochondrial respiratory defects in LKR/SDH and GR/HPR mutants to solidify the connection of these genes to respiratory capacity. It is possible that these proteins do not play a role in mitochondrial respiratory capacity and ATP generation, and their role on migration is independent.

We were unable to obtain knockout cell lines for Seahorse analysis. Thus to assess if Atossa's target metabolic enzymes GR/HPR and LKR/SDH can boost the cellular bioenergetics required for macrophage tissue invasion, we tested if overexpressing either of them in macrophages could rescue the bioenergetic phenotype seen in *atos*$^{PBG}$ macrophages, measuring the pPDH/PDH ratios as a reporter of utilization of the TCA cycle and indirect measure of TCA cycle produced ATP/ADP levels (Fig 8A-B). We simultaneously examined if Porthos, which we have shown affects mitochondrial bioenergetics could rescue the *atos*$^{PBG}$ phenotype (also to address a later query of this reviewer). Interestingly we observed significantly higher pPDH/PDH ratios in *atos*$^{PBG}$ macrophages expressing any of the three Atos targets, Porthos, GR/HPR or LKR/SDH, compared to *atos*$^{PBG}$ mutant embryos (Fig 8A-B). This result strongly supports the conclusion that Atos acts through each of its downstream metabolic proteins to elevate cellular bioenergetics in macrophages. We hypothesize that overexpressing either of these metabolic enzymes which lie upstream of the synthesis of glucose/ Acetyl CoA respectively leads to more fuel being sent into the TCA cycle, compensating for the reduced translation of OxPhos complex components found in the *atossa* mutant due to the lower levels of Porthos. If this is the case we would predict that expressing GR/HPR and LKR/SDH in macrophages could also rescue their germband invasion defect in *atos*$^{PBG}$ embryos. They each could rescue (Fig 8C-D), strongly suggesting that GR/HPR and LKR/SDH enzymes act by increasing cellular energetics and ATP generation to facilitate tissue invasion. We also tested if Atossa's murine orthologs mFAM214A and B could rescue the pPDH/PDH levels to see if the capacity to regulate this bioenergetic pathway was conserved in vertebrates and found that they could (Fig 8E-F).

These results are discussed in the manuscript in lines 476-494 and the data presented in Fig 8.

• There needs to be a better rationale of why ribosomal biogenesis defects cause specific mitochondrial defect instead of wide-spread issues with protein synthesis? Are the identified targets enriched for TISU elements that require cap-dependent translation? mRNAs with these structures usually encode mitochondrial proteins and are sensitive to mTORC1 inhibition. Is there strong overlap with your data set and others that study these mRNAs?
See: 10.1016/j.cmet.2015.02.010 or 10.1101/gr.197566.115
This is an important point, and it is not clear how specific the translation is to mitochondrial proteins, as for example it has been described with the CLUH protein.

To our knowledge TISU elements have not been identified in *Drosophila*. Through an unbiased search we did not find any significantly enriched motifs from the 5'UTRs of the identified target genes.
To determine if the target genes we identified for Porthos contain the TISU sequence we compared their orthologs to the list of TISU containing genes in Sinvani et. al. (DOI:https://doi.org/10.1016/j.cmet.2015.02.010) as suggested. We identified 11 overlapping genes, however, this degree of overlap was not statistically significant (p=0.3769) and accounts for less than 6% of identified targets (See Reviewer Figure panel A below).
We also searched for overlap with previously identified targets of CLUH and found only 5 Porthos targets that are also found in the RNA-seq and Mass Spec datasets from Schatton et al. (2017) (Reviewer Figure panel B-B') and Pla-Martin et al. (2020) (Reviewer Figure panel C-C'). Overall this argues that the target genes of Porthos are not regulated through the same mechanisms as previously identified groups of mitochondrial-related genes.

Finally, we examined the length of the 5'UTRs of Porthos target genes and found that they are significantly shorter than non-target genes (Rev Fig panel D below, shown in Fig 5D in manuscript,). Previous work indicates that in hematopoetic stem cells mRNAs with short 5'UTRs are less well translated under ribosome limiting conditions (see Fig 5A in Khajuria et al: https://www.cell.com/cell/pdf/S0092-8674(18)30213-7.pdf). We hypothesize that Porthos' ability to enhance the translation of the Porthos targets we detected may be due to their shorter 5'UTRs.

These findings are described in the paper in the results in lines 303-312, the bioinformatics analysis approach in the methods in lines 1056-1071 and the 5'UTR length data in Fig 5D.

A

**Porthos polysome targets vs TISU-containing targets (Sivani et. al.)**

| FBID | Symbol | RefSeq (Human) |
|---|---|---|
| FBgn0011227 | ox | NM_013387 |
| FBgn0037602 | SLIRP2 | NM_031210 |
| FBgn0039159 | mRpS24 | NM_032014 |
| FBgn0069354 | Porin2 | NM_003374 |
| FBgn0033550 | CG12341 | NM_001100829 |
| FBgn0031682 | CG5828 | NM_018216 |
| FBgn0036255 | Atg12 | NM_004707 |
| FBgn0033027 | TpnC4 | NM_032328 |
| FBgn0032042 | Scgbeta | NM_000232 |
| FBgn0037310 | Tim17b1 | NM_006335 |
| FBgn0044030 | mRpS14 | NM_022100 |

B **Porthos polysome targets vs RNAseq (Schatton et. al., 2017)**

| FBID | Ensembl (Mus) | Symbol |
|---|---|---|
| FBgn0040391 | ENSMUSG00000046603 | CG2854 |

B' **Porthos polysome targets vs Mass Spec**

| FBID | WikiGene.Name | Symbol |
|---|---|---|
| FBgn0010612 | Atp5l | ATPsynG |

C **Porthos polysome targets vs RNAseq (basal) (Pla-Martin et. al., 2020)**

| FBID | Ensembl (Mus) | Symbol |
|---|---|---|
| FBgn0030932 | ENSMUSG00000006344 | Ggt-1 |
| FBgn0039525 | ENSMUSG00000048856 | CG5646 |

C' **Porthos polysome targets vs RNAseq (HBSS)**

| FBID | Ensembl (Mus) | Symbol |
|---|---|---|
| FBgn0039525 | ENSMUSG00000048856 | CG5646 |

D

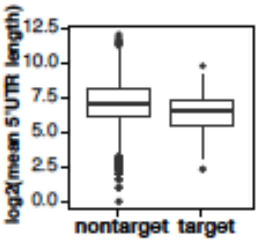


**Reviewer Figure:**

**A** Porthos polysome targets whose orthologs in humans contain TISU sequences**.**

**B-C** Shared targets between Porthos and CLUH targets shown in the two papers.

**D** Analysis of 5' UTR length in mRNAs expressed in S2R+ cells and in Porthos targets

• Please determine if mTORC1 signaling (p-4EBP1) is suppressed during Atossa and Porthos LOF. This could help explain the specific defect in nuclear-encoded mitochondrial gene translation based mentioned above.

To address this and a related request from Reviewer #2 we assessed the activity of dTORC1 signaling pathway, examining the phosphorylation status of the TORC1 kinase target 4EBP1 via Western Blot (Fig. 5I). We saw no significant alteration in the levels of p-4EBP1 in *porthos-KD* S2R+ cells compared to the control (Fig. 5I), arguing that Atossa and Porthos do not upregulate dTORC1 signaling to affect the mRNA translation of a set of nuclear-encoded mitochondrial mRNAs.
We describe this data in lines 329-335 and show it in Fig 5I.

• In the introduction, there is no general principles that elevated mitochondria or PGC1s correlate with invasion, in fact in certain tumors high PGC1a and high mitochondria and oxidative phosphorylation correlate negatively with metastasis, as described by Carracedo's group in Nature Cell Biology (2016) and Cancer Research (2019).

We have changed the introduction to reflect the greater complexity, new text in green, lines 90-93:

"Increases in OxPhos triggered by PGC-1's transcriptional upregulation of mitochondrial proteins can underlie enhanced invasion and metastasis in some cancer types and suppress it in others (LeBleu et al. 2014; Torrano et al., 2016)."

**Minor comments:**

• In the context of Pths rescue in Atossa mutants, can you rescue any molecular phenotypes, e.g. translation defects, mitochondrial respiration, etc.

We observed higher pPDH/PDH ratios in *atos*$^{PBG}$ macrophages expressing Porthos (Fig 8B,D) (described in lines 476-compared to *atos*$^{PBG}$, arguing that Porthos can restore mitochondrial respiration, and thus enhance cellular energy levels, fitting with its ability to rescue macrophage migration into the germband which we had showed in the original version of the paper in Fig 4I-J.
As we do the Pths rescue of the *atos*$^{PBG}$ phenotype in macrophages, assessing translational effects would require Westerns which are not feasible to do on sorted macrophages.

• Also, please verify that Pths overexpression is working in the Atossa mutants through qPCR or Western.

As we overexpress Pths just in macrophages in our experiments, qPCR or Western on embryos would not reveal increased levels and sorting macrophages isolated from embryos for Westerns is extremely time consuming and expensive. We therefore imaged fixed *atos*$^{BGP}$ mutant embryos expressing the HA-tagged Porthos in macrophages and stained them with an anti-HA antibody. As seen in the confocal images the HA-tagged Porthos (shown in green) is found in the nucleus (blue) of macrophages labeled with a cytoplasmic marker in red (Fig. EV4I).

• As an alternative approach, can you rescue the ribosomal assembly defect in Atossa mutants to determine if this is responsible for downstream mitochondrial phenotypes?
One could activate mTORC1 (pharmacological or genetic) to rescue mitochondrial defects present in Atossa mutants since mTORC1 controls ribosome assembly, mitochondrial

gene translation, and PGC-1a activity. Or perhaps overexpress a ribosomal assembly factor that promotes 40S assembly.

We thank the reviewer for this wonderful suggestion. We examined if we could restore the deficiency of macrophage tissue invasion in *atossa*-KD or *porthos*-KD embryos by activating the dTORC1 signaling pathway in macrophages, expressing different RNAis against TORC1 inhibitory components, including Nrpl2, Iml1, and TSC1. We knocked down these different TORC1 suppressors in (Fig 5E,G) *atos*-KD or (Fig 5F,H) *pths*-KD macrophages, and found that they could largely restore their germband invasion. This data supports the conclusion that Porthos acts to enhance ribosomal assembly to enable macrophage germband invasion.

This data is discussed in lines 322-331 in the manuscript and shown in Fig 5E-H.

• Can you express a mutant of Pths in Pths KD cells that fails to localize to the nucleus to assess whether its function in nucleolar ribosomal assembly is important for downstream phenotypes?

We made a mutant form of Porthos lacking the Nuclear Localization Signal (NLS). We observed that Porthos$^{nls-}$ is mainly in the cytoplasm of S2R+ cells (Appendix Fig S1). However, when we overexpressed the FLAG::HA-tagged Pths$^{nls-}$ in macrophages in *atos*$^{PBG}$ mutant embryos, while it showed some effect, it was unable to rescue macrophage germband invasion (Appendix Fig S1). Therefore, we conclude that Porthos nuclear localization plays a critical role to help Atossa to facilitate macrophage invasion.

This data is described in lines 313-321 in the manuscript and shown in Appendix Fig S1.

• Fig 6. b,c - For graphical representation of OCR in bar graph form, I would subtract non-mitochondrial respiration as a baseline correction.

We have changed the figure accordingly.

On that note, it is odd that your non-mitochondrial respiration is so different between genotypes. Perhaps this indicates a seeding/measurement issue since the cells are semi-adherent.

We always seeded the same number of cells. We did not observe changes in the measurements over the time of the experiment making us think it is not likely to be an issue of differential adherence to the probe between the two genotypes. One of Porthos' targets is Lysl oxidase (Appendix Fig S2B), which utilizes oxygen; this could perhaps be an explanation. See lines 417-8.

• Perform either SDS or BN-PAGE analyses for Atossa and Porthos LOFs to confirm nuclear-encoded translation defects.

Unlike in mammalian systems, there are few available antibodies against *Drosophila* proteins. Thus we were unable to obtain antibodies corresponding to any of the direct targets we identified in the RNAseq. To get around this we utilized commercially available antibodies that had been validated for Drosophila (Teixeira et al 2015 PMID 25915123) against subunits of the OxPhos complexes whose protein levels had been shown to depend on the protein levels of our direct targets. Lower levels of the mammalian ortholog of the predicted complex I assembly factor we identified as a target in the RNAseq lead to reduced levels of other complex I proteins, including MT-ND1 (see Formosa et al., 2015

Fig 2C,D). Similarly, in humans the absence of subunit g of complex V, one of our targets, has been shown to lead to lower protein levels of multiple other subunits including ATP synt-β (see He et al., 2018 Fig 5A). In Western blots we found 73% and 31% lower levels of these CI and CV proteins respectively in *pths-KD* S2R+ cells compared to the control (CI MT-ND1, $p<0.0001$; CV ATP synt-β, $p=0.03$) (shown in Fig 6G-H). To test for a possible general deficiency in protein translation we examined the non-target proteins profilin and β tubulin and found no significant change in levels (Fig 6I) (profilin, $p=0.26$; β tubulin, $p=0.55$). In sum, our results argue that Pths does not affect protein translation generally, but is required for the enhanced levels of a subset of proteins, many of which are involved in mitochondrial and metabolic function.

The above description has been added to the results in lines 364-382 and as new Figure panels as mentioned above.

• Please confirm efficacy of complex III and V knockdowns by qPCR or Western.

We have validated the RNAi-downregulation efficacy of complex III and V by performing qPCR on the respective *RNAi*-expressing embryos (Fig. EV5E) (lines 437-8).

• Is there any mRNA-mRNA or protein-protein correlation between Atossa and PGC-1a? Is there any overlap between Atossa and PGC1-a targets? This could strengthen arguments that these pathways can act in concert.

We thank the reviewer for this great suggestion.
*Drosophila* PGC-1 (called Spargel) shows a similar expression pattern in the BDGP *in situ* database to Atos and Pths during the time of macrophage invasion (https://insitu.fruitfly.org/cgi-bin/ex/report.pl?ftype=1&ftext=FBgn0037248); it is found in the midgut, salivary gland, and macrophages, which we confirmed also in our RNA seq analysis from sorted macrophages (Dataset EV2). Additionally Spargel and Porthos did display significant regulatory overlap ($p<0.036$), with 12 shared targets (Appendix Table S1). Thus Atossa and PGC-1 may synergize to stimulate mitochondrial function, with PGC-1 increasing the transcription of proteins whose translation Atossa then enhances. (see lines 391-400).

## References


Siekhaus D, Haesemeyer M, Moffitt O, Lehmann R, RhoL controls invasion and Rap1 localization during immune cell transmigration in *Drosophila*. Nat. Cell Biol. **12**, 605–610 (2010).

Ratheesh A, Biebl J, Vesela J, Smutny M, Papusheva E, Krens SFG, Kaufmann W, Gyoergy A, Casano AM, Siekhaus DE, *Drosophila* TNF modulates tissue tension in the embryo to facilitate macrophage invasive migration. Dev. Cell **3**, 331–346 (2018).

Valoskova K, Biebl J, Roblek M, Emtenani S, Gyoergy A, Misova M, Ratheesh A, Reis-Rodrigues P, Shkarina K, Larsen ISB, Vakhrushev SY, Clausen H, Siekhaus DE, A conserved major facilitator superfamily member orchestrates a subset of O-glycosylation to aid macrophage tissue invasion. eLife **8**, e41801 (2019).

Belyaeva V, Wachner S, Gridchyn I, Linder M, Emtenani S, Gyoergy A, Sibilia M, D Siekhaus, Cortical actin properties controlled by *Drosophila* Fos aid macrophage

infiltration against surrounding tissue resistance. *BioRxiv.* https://doi.org/10.1101/2020.09.18.301481(2020).

Gandin V., Masvidal L., Hulea L., Gravel S. P., Cargnello M., McLaughlan S., Cai Y., Balanathan P., Morita M., Rajakumar A., Furic L., Pollak M., Porco J. A., St-Pierre J., Pelletier J., Larsson O., Topisirovic I., nanoCAGE reveals 5′ UTR features that define specific modes of translation of functionally related MTOR-sensitive mRNAs. *Genome Res.* ***26***, 636–648 (2016). 10.1101/gr.197566.115.

RK Khajuria, M Munschauer, JC Ulirsch, *et al.* Ribosome levels selectively regulate translation and lineage commitment in human hematopoiesis. Cell., 173 (1) (2018), pp. 90-103.e19.

Sinvani H et al., Translational tolerance of mitochondrial genes to metabolic energy stress involves TISU and eIF1-eIF4GI cooperation in start codon selection. *Cell Metab* 21, 479–492 (2015).

**Figures for reviewers**
**Question from Reviewer #2**

**Figure R1**

Dear Dr Siekhaus,

Thank you for submitting your revised manuscript (EMBOJ-2021-109049R) to The EMBO Journal. Your amended study was sent back to the three referees for re-evaluation, and we have received comments from two of them, which I enclose below. Please note that we have editorially assessed your response to the concerns pointed to earlier by referee #2 and found these to be satisfactorily responded to. As you will see, the other referees stated that the issues raised have been adequately addressed and they are broadly now in favour of publication, pending a minor revision.

Thus, we are pleased to inform you that your manuscript has been accepted in principle for publication in The EMBO Journal.

Please consider the remaining points of referee #3 carefully, and address these by introducing caveats in the discussion where appropriate.

In addition, we need you to take care of a number of issues related to formatting and data representation as detailed below, which should be addressed at re-submission.

Please contact me at any time if you have additional questions related to below points.

As you might have noted on our web page, every paper at the EMBO Journal now includes a 'Synopsis', displayed on the html and freely accessible to all readers. The synopsis includes a 'model' figure as well as 2-5 one-short-sentence bullet points that summarize the article. I would appreciate if you could provide this figure and the bullet points.

Thank you for giving us the chance to consider your manuscript for The EMBO Journal. I look forward to your final revision.

Again, please contact me at any time if you need any help or have further questions.

Kind regards,

Daniel Klimmeck

Daniel Klimmeck PhD
Senior Editor
The EMBO Journal

Formatting changes required for the revised version of the manuscript:

>> Please provide up to five keywords to the manuscript.

>> Specify author contributions for M.A and M.G. .

>> Correct the corresponding author to D.E.S. in the author checklist and enter the accession numbers for your GEO data set.

>> Enter the funding information into the Acknowledgements section.

>> Please recheck callouts for Figure 4B in the manuscript text.

>> Recheck labelling for Figure EV5 versus EV7.

>> Add an http link for the GEO data set in data accessibility section, and integrate into one paragraph.

>> The reference format needs to be corrected to EMBO Journal style and 10 author names before et al. .

>>Adjust the title of the 'Conflict of Interest' section to 'Disclosure and Competing Interests Statement'.

>> Compile supplemental figures and legends as one 'Appendix' file with a ToC on its first page, save as PDF, rename figures to "Appendix Figure S1" etc.; Add three Suppl. Tables from the other doc, rename to "Appendix Table S1" etc. .

>> Move the Drosophila genetics information into the Appendix.

>> Dataset EV1 is source data and should be uploaded as source data files, one file per figure.

>> Dataset EV legends: Renumber the remaining datasets and add their legends in a separate tab. Update callouts. The four movies need their legends zipped with the corresponding files.

>> Recheck the bioRxiv reference for Martin et al. 2021 for updates.

>> Please consider additional changes and comments from our production team as indicated by the .doc file enclosed and leave changes in track mode.

Please remember: Digital image enhancement is acceptable practice, as long as it accurately represents the original data and conforms to community standards. If a figure has been subjected to significant electronic manipulation, this must be noted in the figure legend or in the 'Materials and Methods' section. The editors reserve the right to request original versions of figures and the original images that were used to assemble the figure.

Further information is available in our Guide For Authors: https://www.embopress.org/page/journal/14602075/authorguide

We realize that it is difficult to revise to a specific deadline. In the interest of protecting the conceptual advance provided by the work, we recommend a revision within 3 months (2nd May 2022). Please discuss the revision progress ahead of this time with the editor if you require more time to complete the revisions. Use the link below to submit your revision:

Link Not Available

------------------------------------------------

Referee #1:

The authors have addressed my concerns and I recommend the paper be accepted for publication. Congratulations to the authors on a very nice peice of work.

Referee #3:

The authors have addressed the previous reviewers comments. One key point is that they don't think that the mRNA targets have TISU elements, but do note that they have shorter 5' UTRs (which is a hallmark of tisu mRNAs). They don't see changes in mtor signaling as assessed by p-4ebp1 (although they don't see a phospho ladder as one might expect), but mtor hyperactivity does increase the invasion phenotype. The authors should comment on this butI don't think this should affect their publication success, but does make me wonder how correct they are on these points.

# Response to Reviewer comments

Referee #1:

*The authors have addressed my concerns and I recommend the paper be accepted for publication. Congratulations to the authors on a very nice piece of work.*

Many thanks to the reviewer for their kind words.

Referee #3:

*The authors have addressed the previous reviewers comments. One key point is that they don't think that the mRNA targets have TISU elements, but do note that they have shorter 5' UTRs (which is a hallmark of tisu mRNAs). They don't see changes in mtor signaling as assessed by p-4ebp1 (although they don't see a phospho ladder as one might expect), but mtor hyperactivity does increase the invasion phenotype. The authors should comment on this butI don't think this should affect their publication success, but does make me wonder how correct they are on these points*

We thank the reviewer for their critical examination of the manuscript and for their approval of its publication. While TISU element-containing mRNAs have very short 5'UTRs, not all mRNAs with shorter 5'UTRs have TISU elements. TISU element sequences have been found to be enriched in mRNAs with 5' UTRs shorter than 46 bases with a median length of 12 bp (https://doi.org/10.1371/journal.pone.0003094). While the 5'UTRs of the mRNAs whose polysome presence is dependent on Porthos are shorter than that of the average mRNA found in S2 cells, they are not predominantly that short (the median for Porthos targets is 88.5 bp, for all mRNAs 159 bp). We have now added a small table to Figure 5D to highlight this and added this to the Figure legend (line 2237-41).

"The corresponding table shows median/mean 5'UTR lengths in base pairs (bp) for all mRNAs expressed in S2 cells (non-targets) and for the subset whose TE is enhanced by Porthos (Porthos targets)."

A phospho ladder for 4EBP1 is not generally observed in Westerns from *Drosophila* extracts (eg Fig 2A-B, 3A in Miron, Lasko and Sonenberg (2008) MCB, https://journals.asm.org/doi/10.1128/MCB.23.24.9117-9126.2003). Based on known mTOR pathway functions we interpret its hyperactivity as generally increasing ribosomal protein and rRNA levels as well as rRNA processing. This could drive more ribosomal assembly despite the reduced levels of the Porthos helicase whose orthologs aid rRNA processing. The absence of the change in 4EBP1 phosphorylation and the much more specific effect on translation argue that Atossa does not act upstream of mTOR to stimulate its activity. To clarify our description of our interpretation we have now changed the discussion (blue text) to read (lines 615-26):

"Enhancing mitochondrial energy production by raising ribosome levels and thereby increasing the translational efficiency of already existing mRNAs is a complementary mechanism to those previously identified. In response to nutrient availability, mTORC1 stimulates all cap-dependent translation and activates the transcription of ribosomal RNAs and proteins and the processing of rRNAs; this ultimately leads to higher levels of many proteins enabling growth, including those required for mitochondrial function (Borregaard and Herlin, 1982). We find that Atossa also affects ribosomal assembly and the *atos* mutant can be rescued by TOR pathway activation. However the unchanged p-4EBP1 levels and the specific translational effect in the mutants argue that Atossa does not regulate TORC1 activity and that the observed rescue is due to higher general ribosome production. CLUH forms RNA granules, directly binding to, stabilizing and enhancing the translation of mRNAs encoding mitochondrial proteins involved in metabolism, while inhibiting translation of those involved in mitochondrial transcription and translation (Pla-Martin et al., 2020, Schatton et al., 2017)."

## 2nd Revision - Editorial Decision 25th Feb 2022

Dear Dr Siekhaus,

Thank you for submitting the revised version of your manuscript. I have now evaluated your amended manuscript and concluded that the remaining minor concerns have been sufficiently addressed.

Thus, I am pleased to inform you that your manuscript has been accepted for publication in the EMBO Journal.

Please note that it is EMBO Journal policy for the transcript of the editorial process (containing referee reports and your response letter) to be published as an online supplement to each paper. I would thus like to ask for your consent on keeping the additional reviewer figure included in this file.

Also, in case you might NOT want the transparent process file published at all, you will also need to inform us via email immediately. More information is available here:
https://www.embopress.org/page/journal/14602075/authorguide#transparentprocess

------------------------------------------------

Please note that in order to be able to start the production process, our publisher will need and contact you regarding the following forms:

- PAGE CHARGE AUTHORISATION (For Articles and Resources)
http://onlinelibrary.wiley.com/journal/10.1002/(ISSN)1460-2075/homepage/tej_apc.pdf

- LICENCE TO PUBLISH (for non-Open Access)

Your article cannot be published until the publisher has received the appropriate signed license agreement. Once your article has been received by Wiley for production you will receive an email from Wiley's Author Services system, which will ask you to log in and will present them with the appropriate license for completion.

- LICENCE TO PUBLISH for OPEN ACCESS papers

Authors of accepted peer-reviewed original research articles may choose to pay a fee in order for their published article to be made freely accessible to all online immediately upon publication. The EMBO Open fee is fixed at $5,200 (+ VAT where applicable).

We offer two licenses for Open Access papers, CC-BY and CC-BY-NC-ND.
For more information on these licenses, please visit:
http://creativecommons.org/licenses/by-nc-nd/3.0/deed.en_US

- PAYMENT FOR OPEN ACCESS papers

You also need to complete our payment system for Open Access articles. Please follow this link and select EMBO Journal from the drop down list and then complete the payment process: https://authorservices.wiley.com/bauthor/onlineopen_order.asp

Notably, please be reminded that under the DEAL agreement of European scientific institutions with our publisher Wiley, you could be eligible for free publication of your article in the open access format. Please contact either the administration at your institution or Wiley (embojournal@wiley.com) to clarify further questions.

Should you be planning a Press Release on your article, please get in contact with embojournal@wiley.com as early as possible, in order to coordinate publication and release dates.

On a different note, I would like to alert you that EMBO Press is currently developing a new format for a video-synopsis of work published with us, which essentially is a short, author-generated film explaining the core findings in hand drawings, and, as we believe, can be very useful to increase visibility of the work. This has proven to offer a nice opportunity for exposure i.p. for the first author(s) of the study. Please see the following link for representative examples and their integration into the article web page:
https://www.embopress.org/video_synopses
https://www.embopress.org/doi/full/10.15252/embj.2019103932

Please let me know, should you be interested to engage in commissioning a similar video synopsis for your work. According operation instructions are available and intuitive.

Finally, we have noted that the submitted version of your article is also posted on the preprint platform bioRxiv. We would appreciate if you could alert bioRxiv on the acceptance of this manuscript at The EMBO Journal in order to allow for an update of the entry status. Thank you in advance!

If you have any questions, please do not hesitate to call or email the Editorial Office.

Thank you for this contribution to The EMBO Journal and congratulations on a successful publication!

Please consider us again in the future for your most exciting work.

Kind regards,

Daniel Klimmeck

Daniel Klimmeck, PhD
Senior Editor
The EMBO Journal
EMBO
Postfach 1022-40
Meyerhofstrasse 1
D-69117 Heidelberg
contact@embojournal.org
Submit at: http://emboj.msubmit.net

EMBO PRESS

YOU MUST COMPLETE ALL CELLS WITH A PINK BACKGROUND 🡻

PLEASE NOTE THAT THIS CHECKLIST WILL BE PUBLISHED ALONGSIDE YOUR PAPER

Corresponding Author Name: Daria E. Siekhaus

Journal Submitted to: EMBOJ

Manuscript Number: EMBOJ-2021-109049

USEFUL LINKS FOR COMPLETING THIS FORM

http://www.antibodypedia.com
http://1degreebio.org
http://www.equator-network.org/reporting-guidelines/improving-bioscience-research-repor

http://grants.nih.gov/grants/olaw/olaw.htm
http://www.mrc.ac.uk/Ourresearch/Ethicsresearchguidance/Useofanimals/index.htm
http://ClinicalTrials.gov
http://www.consort-statement.org
http://www.consort-statement.org/checklists/view/32-consort/66-title

http://www.equator-network.org/reporting-guidelines/reporting-recommendations-for-tumo

http://datadryad.org

http://figshare.com

http://www.ncbi.nlm.nih.gov/gap

http://www.ebi.ac.uk/ega

http://biomodels.net/

http://biomodels.net/miriam/
http://jjj.biochem.sun.ac.za
https://osp.od.nih.gov/biosafety-biosecurity-and-emerging-biotechnology/
http://www.selectagents.gov/

## Reporting Checklist For Life Sciences Articles (Rev. June 2017)

This checklist is used to ensure good reporting standards and to improve the reproducibility of published results. These guidelines are consistent with the Principles and Guidelines for Reporting Preclinical Research issued by the NIH in 2014. Please follow the journal's authorship guidelines in preparing your manuscript.

### A- Figures
### 1. Data
**The data shown in figures should satisfy the following conditions:**

- the data were obtained and processed according to the field's best practice and are presented to reflect the results of the experiments in an accurate and unbiased manner.
- figure panels include only data points, measurements or observations that can be compared to each other in a scientifically meaningful way.
- graphs include clearly labeled error bars for independent experiments and sample sizes. Unless justified, error bars should not be shown for technical replicates.
- if n< 5, the individual data points from each experiment should be plotted and any statistical test employed should be justified
- Source Data should be included to report the data underlying graphs. Please follow the guidelines set out in the author ship guidelines on Data Presentation.

### 2. Captions

**Each figure caption should contain the following information, for each panel where they are relevant:**

- a specification of the experimental system investigated (eg cell line, species name).
- the assay(s) and method(s) used to carry out the reported observations and measurements
- an explicit mention of the biological and chemical entity(ies) that are being measured.
- an explicit mention of the biological and chemical entity(ies) that are altered/varied/perturbed in a controlled manner.

- the exact sample size (n) for each experimental group/condition, given as a number, not a range;
- a description of the sample collection allowing the reader to understand whether the samples represent technical or biological replicates (including how many animals, litters, cultures, etc.).
- a statement of how many times the experiment shown was independently replicated in the laboratory.
- definitions of statistical methods and measures:
  - common tests, such as t-test (please specify whether paired vs. unpaired), simple $\chi 2$ tests, Wilcoxon and Mann-Whitney tests, can be unambiguously identified by name only, but more complex techniques should be described in the methods section;
  - are tests one-sided or two-sided?
  - are there adjustments for multiple comparisons?
  - exact statistical test results, e.g., P values = x but not P values < x;
  - definition of 'center values' as median or average;
  - definition of error bars as s.d. or s.e.m.

Any descriptions too long for the figure legend should be included in the methods section and/or with the source data.

**In the pink boxes below, please ensure that the answers to the following questions are reported in the manuscript itself. Every question should be answered. If the question is not relevant to your research, please write NA (non applicable). We encourage you to include a specific subsection in the methods section for statistics, reagents, animal models and human subjects.**

### B- Statistics and general methods

| | Please fill out these boxes 🡻 (Do not worry if you cannot see all your text once you press return) |
|---|---|
| 1.a. How was the sample size chosen to ensure adequate power to detect a pre-specified effect size? | NA |
| 1.b. For animal studies, include a statement about sample size estimate even if no statistical methods were used. | For analysis of Drosophila embryos, we generally utilized at least an n of 15, which in our experience lets us detect robust phenotypes. For live imaging we utlized at least n=3. There were no ``animal`` studies. |
| 2. Describe inclusion/exclusion criteria if samples or animals were excluded from the analysis. Were the criteria pre-established? | We only excluded samples that were the wrong stage in our analysis of timed embryo collections, or entire experiments if the antibody staining didn't work even in the control. |
| 3. Were any steps taken to minimize the effects of subjective bias when allocating animals/samples to treatment (e.g. randomization procedure)? If yes, please describe. | Our analysis was conducted on Drosophila embryos collected during a certain time period from mothers of one genotype. There was no subjective bias utilized in which mothers and fathers to point into a cage. We just took all the adults from a certain genotype. |
| For animal studies, include a statement about randomization even if no randomization was used. | No animal study was conducted in this paper. If what we did above is considered randomization, then we conducted randomization. |
| 4.a. Were any steps taken to minimize the effects of subjective bias during group allocation or/and when assessing results (e.g. blinding of the investigator)? If yes please describe. | We always converted the names of all the images nto a code before we examined them, so that they could be analyzed blind. This is described in the methods section on statistics. |
| 4.b. For animal studies, include a statement about blinding even if no blinding was done | We did blinding. |
| 5. For every figure, are statistical tests justified as appropriate? | Yes |
| Do the data meet the assumptions of the tests (e.g., normal distribution)? Describe any methods used to assess it. | We always tested for a normal distribution before analysis using Prism. This is described in the methods section on statistics. |
| Is there an estimate of variation within each group of data? | Yes, we always calculated Standard Deviation and then Standard Error. This is described in the Figure legends and in Dataset EV1 |

| | |
|---|---|
| Is the variance similar between the groups that are being statistically compared? | Yes |

### C- Reagents

| | |
|---|---|
| 6. To show that antibodies were profiled for use in the system under study (assay and species), provide a citation, catalog number and/or clone number, supplementary information or reference to an antibody validation profile. e.g., Antibodypedia (see link list at top right), 1DegreeBio (see link list at top right). | We did this, including such information in Appendix Table S4 |
| 7. Identify the source of cell lines and report if they were recently authenticated (e.g., by STR profiling) and tested for mycoplasma contamination. | We utilized Drosophila S2R+ cells which were a gift from the Knoeblich lab. The cell lines were tested for mycoplasma contamination before utilization. This is described in the `methods |

* for all hyperlinks, please see the table at the top right of the document

### D- Animal Models

| | |
|---|---|
| 8. Report species, strain, gender, age of animals and genetic modification status where applicable. Please detail housing and husbandry conditions and the source of animals. | We have done so, in the methods for the more general aspects, and the stage in the Figure legends. |
| 9. For experiments involving live vertebrates, include a statement of compliance with ethical regulations and identify the committee(s) approving the experiments. | NA |
| 10. We recommend consulting the ARRIVE guidelines (see link list at top right) (PLoS Biol. 8(6), e1000412, 2010) to ensure that other relevant aspects of animal studies are adequately reported. See author guidelines, under 'Reporting Guidelines'. See also: NIH (see link list at top right) and MRC (see link list at top right) recommendations. Please confirm compliance. | NA |

### E- Human Subjects

| | |
|---|---|
| 11. Identify the committee(s) approving the study protocol. | NA |
| 12. Include a statement confirming that informed consent was obtained from all subjects and that the experiments conformed to the principles set out in the WMA Declaration of Helsinki and the Department of Health and Human Services Belmont Report. | NA |
| 13. For publication of patient photos, include a statement confirming that consent to publish was obtained. | NA |
| 14. Report any restrictions on the availability (and/or on the use) of human data or samples. | NA |
| 15. Report the clinical trial registration number (at ClinicalTrials.gov or equivalent), where applicable. | NA |
| 16. For phase II and III randomized controlled trials, please refer to the CONSORT flow diagram (see link list at top right) and submit the CONSORT checklist (see link list at top right) with your submission. See author guidelines, under 'Reporting Guidelines'. Please confirm you have submitted this list. | NA |
| 17. For tumor marker prognostic studies, we recommend that you follow the REMARK reporting guidelines (see link list at top right). See author guidelines, under 'Reporting Guidelines'. Please confirm you have followed these guidelines. | NA |

### F- Data Accessibility

| | |
|---|---|
| 18: Provide a "Data Availability" section at the end of the Materials & Methods, listing the accession codes for data generated in this study and deposited in a public database (e.g. RNA-Seq data: Gene Expression Omnibus GSE39462, Proteomics data: PRIDE PXD000208 etc.) Please refer to our author guidelines for 'Data Deposition'.<br><br>Data deposition in a public repository is mandatory for:<br>a. Protein, DNA and RNA sequences<br>b. Macromolecular structures<br>c. Crystallographic data for small molecules<br>d. Functional genomics data<br>e. Proteomics and molecular interactions | RNA seq and Polysome profiling data deposited at Gene Expression Omnibus (GEO) as GSE167134 |
| 19. Deposition is strongly recommended for any datasets that are central and integral to the study; please consider the journal's data policy. If no structured public repository exists for a given data type, we encourage the provision of datasets in the manuscript as a Supplementary Document (see author guidelines under 'Expanded View' or in unstructured repositories such as Dryad (see link list at top right) or Figshare (see link list at top right). | See above. |
| 20. Access to human clinical and genomic datasets should be provided with as few restrictions as possible while respecting ethical obligations to the patients and relevant medical and legal issues. If practically possible and compatible with the individual consent agreement used in the study, such data should be deposited in one of the major public access-controlled repositories such as dbGAP (see link list at top right) or EGA (see link list at top right). | NA |
| 21. Computational models that are central and integral to a study should be shared without restrictions and provided in a machine-readable form. The relevant accession numbers or links should be provided. When possible, standardized format (SBML, CellML) should be used instead of scripts (e.g. MATLAB). Authors are strongly encouraged to follow the MIRIAM guidelines (see link list at top right) and deposit their model in a public database such as Biomodels (see link list at top right) or JWS Online (see link list at top right). If computer source code is provided with the paper, it should be deposited in a public repository or included in supplementary information. | NA |

### G- Dual use research of concern

| | |
|---|---|
| 22. Could your study fall under dual use research restrictions? Please check biosecurity documents (see link list at top right) and list of select agents and toxins (APHIS/CDC) (see link list at top right). According to our biosecurity guidelines, provide a statement only if it could. | NA |
